# Pitavastatin and Atorvastatin Double-Blind Randomized ComPArative Study among HiGh-Risk Patients, Including ThOse with Type 2 Diabetes Mellitus, in Taiwan (PAPAGO-T Study)

**DOI:** 10.1371/journal.pone.0076298

**Published:** 2013-10-01

**Authors:** Ping-Yen Liu, Liang-Yu Lin, Hung-Ju Lin, Chien-Hsun Hsia, Yi-Ren Hung, Hung-I Yeh, Tao-Cheng Wu, Ju-Yi Chen, Kuo-Liong Chien, Jaw-Wen Chen

**Affiliations:** 1 Division of Cardiology, Internal Medicine, National Cheng Kung University Hospital, Tainan, Taiwan; 2 Institute of Clinical Medicine, National Cheng Kung University, Tainan, Taiwan; 3 Division of Endocrinology and Metabolism, Internal Medicine, National Yang-Ming University and Taipei Veterans General Hospital, Taipei, Taiwan; 4 Division of Cardiology, Internal Medicine, National Taiwan University Hospital, Taipei, Taiwan; 5 Division of Cardiology, Internal Medicine, Changhua Christian Hospital, Changhua, Taiwan; 6 Division of Endocrinology and Metabolism, Internal Medicine, Tri-Service General Hospital, Taipei, Taiwan; 7 Division of Cardiology, Internal Medicine, Mackay Memorial Hospital, Taipei, Taiwan; 8 Division of Cardiology, Internal Medicine, National Yang-Ming University and Taipei Veterans General Hospital, Taipei, Taiwan; 9 Institute of Epidemiology and Preventive Medicine, College of Public Health, National Taiwan University, Taipei, Taiwan; 10 Institute of Pharmacology, National Yang-Ming University School of Medicine and Department of Medical Research and Education, Taipei Veterans General Hospital, Taipei, Taiwan; Postgraduate Medical Institute & Hull York Medical School, University of Hull, United Kingdom

## Abstract

**Background:**

Evidence about the efficacy and safety of statin treatment in high-risk patients with hypercholesterolemia is available for some populations, but not for ethnic Chinese. To test the hypothesis that treatment with pitavastatin (2 mg/day) is not inferior to treatment with atorvastatin (10 mg/day) for reducing low-density lipoprotein cholesterol (LDL-C), a 12-week multicenter collaborative randomized parallel-group comparative study of high-risk ethnic Chinese patients with hypercholesterolemia was conducted in Taiwan. In addition, the effects on other lipid parameters, inflammatory markers, insulin-resistance-associated biomarkers and safety were evaluated.

**Methods and Results:**

Between July 2011 and April 2012, 251 patients were screened, 225 (mean age: 58.7 ± 8.6; women 38.2% [86/225]) were randomized and treated with pitavastatin (n = 112) or atorvastatin (n = 113) for 12 weeks. Baseline characteristics in both groups were similar, but after 12 weeks of treatment, LDL-C levels were significantly lower: pitavastatin group = −35.0 ± 14.1% and atorvastatin group = −38.4 ± 12.8% (both: p < 0.001). For the subgroup with diabetes mellitus (DM) (n = 125), LDL-C levels (−37.1 ± 12.9% vs. −38.0 ± 13.1%, p = 0.62) were similarly lowered after either pitavastatin (n = 63) or atorvastatin (n = 62) treatment. Triglycerides, non-high density lipoprotein cholesterol, and apoprotein B were similarly and significantly lower in both treatment groups. In non-lipid profiles, HOMA-IR and insulin levels were higher to a similar degree in both statin groups. Hemoglobin A_1_C was significantly (p = 0.001) higher in the atorvastatin group but not in the pitavastatin group. Both statins were well tolerated, and both groups had a similar low incidence of treatment-emergent adverse events.

**Conclusion:**

Both pitavastatin (2 mg/day) and atorvastatin (10 mg/day) were well tolerated, lowered LDL-C, and improved the lipid profile to a comparable degree in high-risk Taiwanese patients with hypercholesterolemia.

**Trial Registration:**

ClinicalTrials.gov NCT01386853 http://clinicaltrials.gov/ct2/show/NCT01386853?term=NCT01386853&rank=1

## Introduction

Accumulated clinical trials have shown that individuals with elevated low-density lipoprotein cholesterol (LDL-C) have an increased risk of coronary heart disease (CHD); they also report the mortality rates in patients with and without documented CHD [1,2]. Different types of statins are clinically available, but the choice of statin is usually based on its individual pharmacological properties, such as its efficacy for LDL-C reduction and its metabolic dependence on hepatic enzymes.

In addition to LDL-C, some species of triglyceride (TG)-rich lipoproteins are also known to be atherogenic; notable among these are cholesterol-enriched remnant lipoproteins. Moreover, very low-density lipoprotein cholesterol (VLDL-C) is a marker for atherogenic VLDL remnants. Non-high density lipoprotein (non-HDL) cholesterol, which is the sum of VLDL-C and LDL-C, therefore constitutes “atherogenic cholesterol”; it is proposed as a secondary target of lipid-lowering therapy to prevent CHD [3,4].

Type 2 diabetes mellitus (DM) is an important risk factor for developing cardiovascular disease. Type 2 DM has an atherogenic lipid profile consisting of elevated TGs and low HDL-C, which results in high non-HDL-C levels. Thus, a preferable characteristic for an ideal statin to treat metabolic syndrome and dyslipidemia in type 2 DM would be targeted to reduce non-HDL-C and LDL-C, and to elevate HDL-C. The Collaborative Atorvastatin Diabetes Study (CARDS) found that 10 mg daily of atorvastatin, a potent commercial LDL-C-lowering agent, reduced non-HDL-C by 36% [5] and prevented cardiovascular events in various populations at high risk of arteriosclerotic disease [5,6].

Pitavastatin (2 mg daily, a medium-strength dose) can lower LDL-C by 42% [7]. It was also reported that pitavastatin reduced TGs and remnant lipoprotein cholesterol by 29% and 31%, respectively, and increased HDL-C by 10% in Japanese patients with DM [8]. It also has unique pharmacokinetic properties: although lipophilic, unlike atorvastatin, which is metabolized by CYP3A4, its metabolism is not dependent on cytochrome P450 [9]. In the CHIBA study in Japan, pitavastatin (2 mg daily) was as effective as atorvastatin (10 mg daily) at lowering non-HDL-C by 39.0-40.3% [10]. The efficacy of these two statins was further analyzed in a subgroup of patients with metabolic syndrome. In addition, similar changes were found for total cholesterol (TC) and LDL-C in both treatment groups. HDL-C significantly increased in the pitavastatin group, but not in the atorvastatin group. However, the CHIBA study was an open-label study. It would be interesting to know if the non-HDL-C or LDL-C effect by standard-dose statin treatment may vary on another high-risk Asian population, either with or without type 2 DM.

We tested, in a double-blind study, the hypothesis that pitavastatin (2 mg/day) is not inferior to atorvastatin (10 mg/day) for reducing LDL-C. This collaborative multicenter study ran for 12 weeks with high-risk Taiwanese patients with hypercholesterolemia. In addition, safety, the effects on other lipid parameters, inflammatory markers, and insulin resistance-associated biomarkers, as well as the relationship between lipid-lowering efficacy and body mass index, were evaluated.

## Methods

The protocol for this trial and supporting CONSORT checklist are available as supporting information; see Checklist S1 and Protocol S1.

### Ethical Approval

All research involving human participants was approved by the authors’ institutional review boards (IRBs) and by the Taiwan Department of Health before the trial began. Informed consent was obtained from all participants, and all clinical investigations were conducted according to the principles expressed in the Declaration of Helsinki.

### Study Design

This Pitavastatin and Atorvastatin randomized comPArative study among hiGh-risk patients including thOse with Type 2 diabetes mellitus in Taiwan (PAPAGO-T study) was a randomized, multicenter, double-blinded, non-inferiority study comparing the efficacy and safety of pitavastatin with that of atorvastatin in high-risk Taiwanese patients with hypercholesterolemia and with or without DM. This trial (NCT01386853) was registered at http://clinicaltrials.gov/ct2/show/NCT01386853?term=NCT01386853&rank=1 [11]. The information for this trial registration is available as supporting information; see Registration Protocol S2.

Patients were recruited from 6 medical centers in Taiwan. After a 4-week dietary lead-in, eligible patients were randomized into 2 equal 12-week treatment groups: Pitavastatin (2 mg/day) (PTV) or Atorvastatin (10 mg/day) (ATV). Patient registration and randomization were done using an interactive voice response system (Virginia Contract Research Organization Co., Ltd., Taipei, Taiwan). The random codes were generated based on permuted block randomization and stratified by type 2 DM status. Statins and fibrates were discontinued during the lead-in period to eliminate any effects of premedication. During the study period, fibrates, other statins, probucol, and cyclosporine [12] were prohibited. No changes were made in types or doses of permitted lipid-lowering drugs (i.e., eicosapentaenoic acid) or in types or doses of medications used to treat hypertension or diabetes. No changes were made in lifestyle guidance, including exercise and diet, throughout the lead-in and dosing periods.

### Patients

Eligible patients were men and women aged 20 or older with fasting LDL-C higher than 100 mg/dl. In addition, to be considered “high-risk”, a patient had to meet at least one of the following criteria (NCEP ATP III guideline): 1) documented CHD; 2) type 2 DM; 3) if the patient had fewer than 2 risk factors (other than LDL) present in the following items without CHD or a CHD risk equivalent, a 10-year (short-term) CHD risk had to be assessed with a Framingham score > 20%: [A] female: ≥ 55 years old, or male: ≥ 45 years old; [B] fasting high-density lipoprotein cholesterol (HDL-C) < 40mg/dL; [C] a family history of premature CHD (CHD in first-degree male relative < 55 years; CHD in first-degree female relative < 65 years); [D] hypertension (BP ≥ 140/90 mmHg or treated with anti-hypertensive agents); [E] HDL-C ≥ 60 mg/dL counted as a “negative” risk factor; its presence removed one risk factor from the total count.

Major exclusion criteria, besides the medication prohibitions stated above, were: 1) a history of hypersensitivity to statins; 2) hepatic dysfunction [aspartate aminotransferase (AST) or alanine aminotransferase (ALT) > 100 IU/L], suspected hepatic metabolism disorders or biliary obstruction (acute hepatitis, acute exacerbation of chronic hepatitis, liver cirrhosis, liver cancer and jaundice), or renal dysfunction (serum creatinine > 1.5 mg/dL); 3) pregnancy, possible pregnancy, or breastfeeding; 4) poorly controlled diabetes (HbA_1_C > 9.0%).

### Assessment

The following information was obtained during the dietary lead-in period before randomization: gender, age, height, body weight, menopause (women only), familial hypercholesterolemia, concomitant diseases, past medical history, family history of CHD. Blood samples were collected after overnight fasting. In the follow-up period, blood samples were checked at 4 weeks and 12 weeks. We evaluated the primary efficacy variable in the LDL-C lowering effect as well as the secondary efficacy variable including lipid (both at 4 and 12 weeks) and non-lipid (at 4 weeks) efficacies, such as HDL-C, TG, non-HDL-C, Apo A1, and Apo B levels, and the fasting plasma glucose, fasting insulin, HbA1c, free fatty acid, ADMA, and HOMA-IR parameters.

The primary endpoint was percent change from baseline in LDL-C level after 12 weeks of treatment. Secondary endpoints included percent changes from baseline in TC, TG, and HDL-C. Serum TC, TG, non-HDL-C, and HDL-C levels were measured at one central laboratory.

Patients were diagnosed with type 2 DM if they had fasting plasma glucose ≥ 126 mg/dL or random plasma glucose ≥ 200 mg/dL with classic symptoms, or if they were taking diabetes medication [13]. Safety was assessed from the incidence of adverse events and abnormal laboratory data.

### Statistical Analysis

The primary endpoint in the present study was the percentage change in LDL-C. We determined the sample size on the basis of the percentage change in the LDL-C, which is the most commonly used endpoint for assessing the efficacy of statins. For a difference in the mean percentage change in the LDL-C at week 12 between pitavastatin and atorvastatin, standard deviation (SD) of the difference in the mean percentage change of the LDL-C of 14% [14,15], non-inferiority margin of 6%, significance level of 5% in a two-tailed test, and a power level of 80%, the number of patients per group was determined to be 87 (total: 174). Taking into account dropouts, we conducted the present study with 100 patients per group, that is, 200 patients in total. Statistical analysis was done using JMP (SAS Institute, Cary, NC, USA). Numerical data are expressed as mean ± SD and a 95% confidence interval (CI). The ANCOVA was first used to test the difference of LDL-C by incorporating treatment, diabetes mellitus, CHD, and baseline LDL-C. Then, a Wilcoxon rank sum test or a 2-sample *t* test was used to compare continuous variables between the pitavastatin and atorvastatin groups as the ancillary analyses. A χ^2^ test was used to analyze categorical data. A 1-sample *t* test was used to assess the efficacy of each drug at week 12. In all cases, 2-sided tests were done. Efficacy was evaluated based on the intention to treat a general population. For the primary endpoint, there was no significant difference between intention to treat and per protocol analyses.

## Results

### Patient characteristics

**Figure 1:**
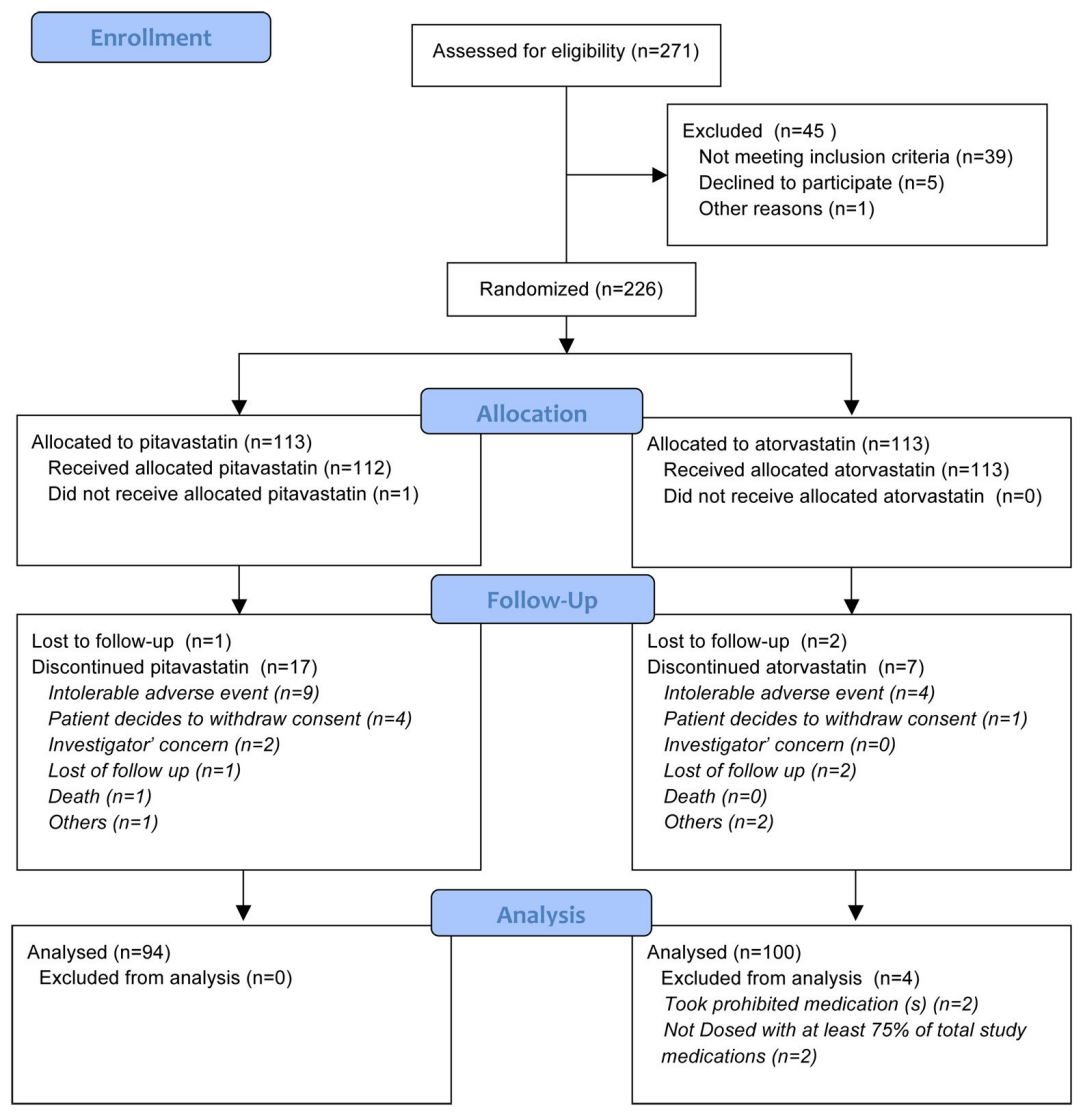
Enrollment and consort of study participants treated with pitavastatin or atorvastatin.

Of the 251 patients screened between July 2011 and April 2012, finally, 225 (mean age 58.7 ± 8.6; women 38.2% (86/225) were randomly assigned to the PTV (n = 112) or ATV (n = 113) group (Figure **1**). The baseline characteristics and parameters for the patients in both groups are shown in Table **1**. Subgroup analysis was done for the 125 patients with type 2 DM (PTV group: 63; ATV group: 62).

**Table 1:** Baseline characteristics for patients in pitavastatin (PTV) and atorvastatin (ATV) groups.

	Total	Type 2 DM	PTV	ATV	PTV vs. ATV
Characteristic	(n = 225)	(n = 125)	(n = 112)	(n = 113)	p-value*
Age (years)	58.7 ± 8.6	58.8 ± 8.4	58.7 ± 9.3	58.7 ± 7.9	0.98
Male, n (%)	139 (61.8%)	67 (53.6%)	69 (61.6%)	70 (61.9%)	0.95
BMI (kg/m^2^)	26.4 ± 3.5	26.5 ± 3.8	26.6 ± 3.6	26.2 ± 3.4	0.51
Body weight (Kg)	69.7 ± 12.2	69.1 ± 13.5	70.8 ± 13.0	68.8 ± 11.4	0.22
Systolic pressure (mmHg)	128 ± 15	128 ± 15	127 ± 14	129 ± 16	0.29
Diastolic pressure (mmHg)	78 ± 9	77 ± 10	76 ± 9	79 ± 10	0.10
Medical history (n (%))					
Coronary artery disease	78 (34.7%)	16 (12.8%)	38 (33.9%)	40 (35.4%)	0.82
Hypertension	17 (75.6%)	88 (70.4%)	85 (75.9%)	85 (75.2%)	0.91
Type 2 diabetes mellitus	125 (55.6%)	125 (100%)	62 (55.4%)	63 (55.8%)	0.95
Non smoker	184 (81.8%)	104 (83.2%)	94 (83.9%)	90 (79.7%)	0.66
Lipid profile					
Total cholesterol (mg/dL)	213.7 ± 32.3	209.8 ± 31.1	213.0 ± 31.5	214.0 ± 33.1	0.90
TG (mg/dL)	154.5 ± 65.6	151.1 ± 67.2	156.0 ± 67	153.0 ± 64.4	0.72
LDL-C (mg/dL)	150.4 ± 28.2	146.3 ± 25.6	149.6 ± 26.4	151.2 ± 30	0.66
HDL-C (mg/dL)	48.6 ± 10.8	48.7 ± 11.0	48.7 ± 10.8	48.5 ± 10.9	0.89
Apo A1 (mg/dL)	136.4 ± 18.8	136.9 ± 19.0	137.0 ± 16.8	136.0 ± 20.7	0.83
Apo B (mg/dL)	120.4 ± 20.7	118.0 ± 20.5	120.0 ± 18.7	121.0 ± 22.5	0.83
Non-lipid profile					
Hemoglobin (g/dL)	14.6 ± 1.4	14.3 ± 1.5	14.6 ± 1.5	14.5 ± 1.4	0.74
AST (U/L)	27.1 ± 9.1	28.1 ± 10.5	27.5 ± 10.7	26.8 ± 7.1	0.59
ALT (U/L)	31.1 ± 16.6	32.8 ± 18.9	31.5 ± 17.9	30.6 ± 15.1	0.68
Gamma GT (U/L)	37.2 ± 32.5	37.4 ± 37.1	37.6 ± 37.0	36.8 ± 27.5	0.87
Total bilirubin (mg/dL)	0.8 ± 0.3	0.8 ± 0.3	0.8 ± 0.3	0.8 ± 0.3	0.36
BUN (mg/dL)	15.7 ± 3.7	16.0 ± 3.9	15.7 ± 3.6	15.7 ± 3.9	1.00
Creatinine (mg/dL)	0.84 ± 0.22	0.8 ± 0.20	0.84 ± 0.24	0.83 ± 0.19	0.74
hsCRP (mg/dL)	0.25 ± 0.80	0.33 ± 1.05	0.17 ± 0.23	0.33 ± 1.1	0.13
CPK (U/L)	128.0 ± 73	125.0 ± 71	125.0 ± 65	131.0 ± 81	0.58
LDH (U/L)	193.0 ± 35	195.0 ± 37	192.0 ± 32	194.0 ± 38	0.70
Fatty acid (mmol/L)	1.0 ± 0.5	1.1 ± 0.5	1.0 ± 0.5	1.0 ± 0.6	0.85
ADMA (μmol/L)	0.58 ± 0.13	0.59 ± 0.13	0.58 ± 0.13	0.59 ± 0.13	0.55
Fasting glucose (mg/dL)	115.3 ± 25	129.6 ± 24.6	117.0 ± 26.8	114.0 ± 23.1	0.14
HbA_1_C (%)	6.5 ± 0.8	7.0 ± 0.8	6.5 ± 0.9	6.5 ± 0.8	0.41
Insulin (μU/ml)	12.6 ± 7.8	12.9 ± 8.2	13.1 ± 8.2	12.1 ± 7.5	0.35
HOMA-IR	3.7 ± 2.8	4.2 ± 3.3	3.9 ± 3.3	3.4 ± 2.3	0.15

Continuous variables are represented by mean ± SD. ADMA = asymmetric dimethylarginine; ALT = alanine aminotransferase; Apo A1 = apolipoprotein A1; Apo B = apolipoprotein B; AST = aspartate transaminase; BMI = body mass index; BUN = blood urea nitrogen; CPK = creatine phosphokinase; DM = diabetes mellitus; Gamma GT = gamma-glutamyl transpeptidase; HDL-C = high-density lipoprotein cholesterol; HOMA-IR = homeostatic model assessment-insulin resistance; hsCRP = high-sensitive C-reactive protein; LDH = lactate dehydrogenase; LDL-C = low-density lipoprotein cholesterol; TG = triglyceride. * All p-values were > 0.05 between PTV and ATV groups.

### Efficacy

**Figure 2:**
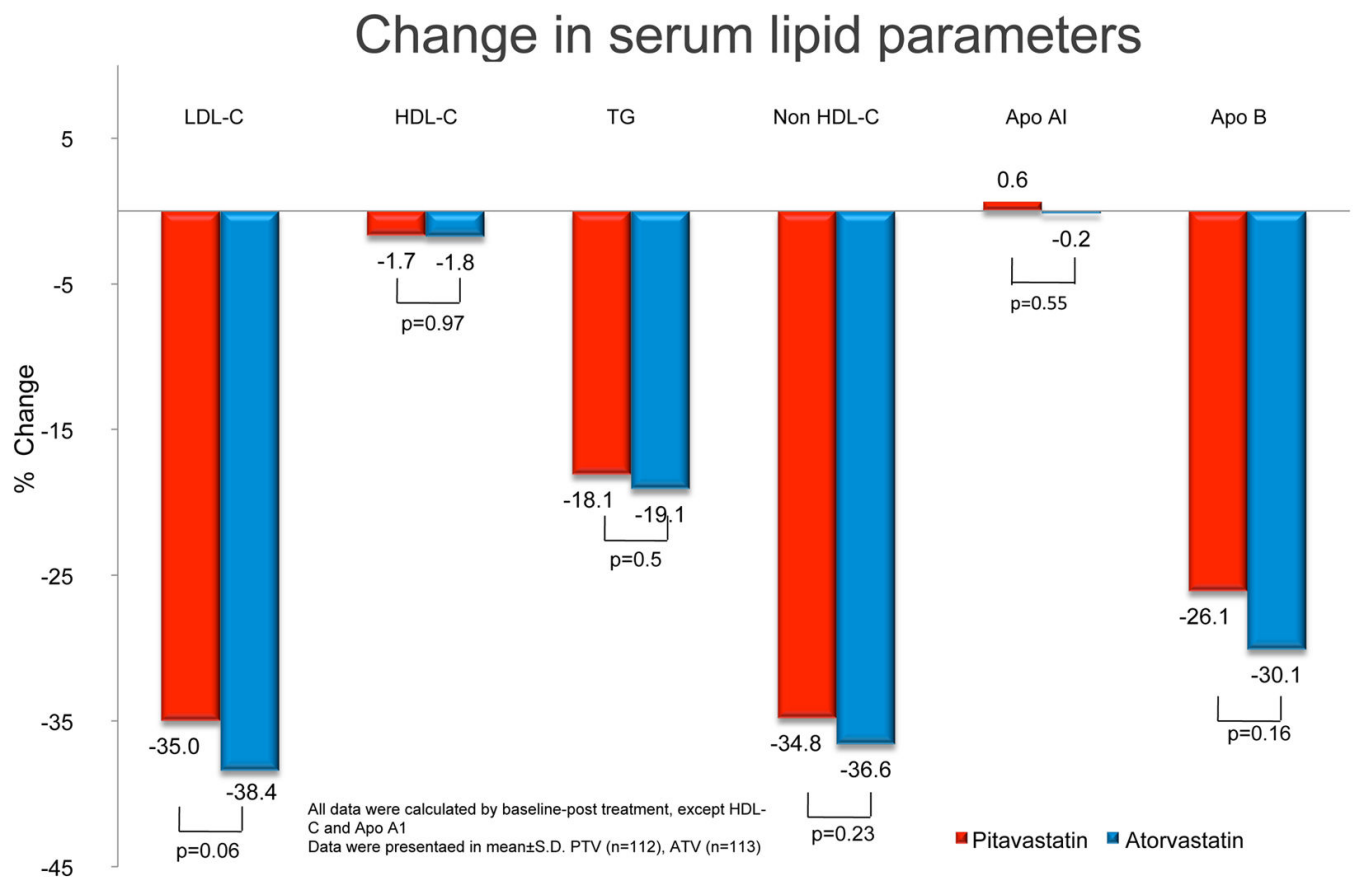
Changes in lipid profiles before and after 12 weeks of treatment with pitavastatin (PTV) (2 mg) or atorvastatin (ATV) (10 mg). There is no significant difference in the percentage change of low-density lipoprotein-cholesterol (LDL-C), high-density lipoprotein-cholesterol (HDL-C), triglyceride (TG), non-HDL-C, apolipoprotein A1 (Apo A1), or apolipoprotein B (Apo B) levels from baseline, between the PTV and ATV groups. Both statins similarly but significantly reduced LDL-C levels after 12 weeks of treatment. PTV and ATV significantly reduced TG, non-HDL-C, and Apo B; the percentage changes were not significantly different.

There was no significant difference in the primary end point: percentage change in the LDL-C level from baseline between the PTV and ATV groups. LDL-C levels were significantly lower in both groups after 12 weeks of treatment: PTV by −35.0 ± 14.1% and ATV by −38.4 ± 12.8% (both p < 0.001) (Table **2** and Figure **2**). The values and percentage changes from baseline in HDL-C, TG, non-HDL-C, Apo A1, and Apo B (secondary endpoints) are shown in Table **2** and Figure **2**; the results of intergroup comparison were comparable. The available lipid profiles for LDL-C, HDL-C, TG, and non-HDL-C were similar between weeks 4 and 12, which indicated no time-effect between week 4 and the end of the study. Furthermore, both pitavastatin and atorvastatin significantly reduced TG (−18.1 ± 32.9% vs. −19.1 ± 26.4%, both p < 0.0001), non-HDL-C (−34.8 ± 11.9% vs. −36.6 ± 10.8%, both p < 0.0001), and Apo B (−26.1 ± 11.9% vs. −30.1 ± 14.0%, both p < 0.0001). Neither had any significant effect on HDL-C levels (−1.7 ± 11.9% vs. −1.8 ± 11.5%, both p > 0.05) or Apo A1 levels (+0.6 ± 14.3% vs. −0.2 ± 9.4%, both p > 0.05).

**Table 2:** Lipid profiles of values and percentage changes from baseline after 4 weeks and 12 weeks of treatment in both pitavastatin (PTV) and atorvastatin (ATV) groups.

	Baseline	4 weeks	12 weeks	Percentage change (%)#	Baseline vs. week 12 p-value
Total cholesterol (mg/dL)					
PTV	213.0 ± 31.5	155.7 ± 27.1	154.9 ± 28.4	−27.3 ± 10.0	< 0.001
ATV	214.0 ± 33.1	155.0 ± 29.0	152.0 ± 28.3	−28.7 ± 9.1	< 0.001
TG (mg/dL)					
PTV	156.0 ± 67	118.9 ± 51.4	118.4 ± 51.3	−18.1 ± 32.9	< 0.001
ATV	153.0 ± 64.4	117.9 ± 50.3	116.4 ± 49.7	−19.1 ± 26.4	< 0.001
LDL-C (mg/dL)					
PTV	149.6 ± 26.4	96.5 ± 26.1	97.2 ± 26.9	−35.0 ± 14.1	< 0.001
ATV	151.2 ± 30	95.0 ± 25.8	92.8 ± 26.0	−38.4 ± 12.8	< 0.001
HDL-C (mg/dL)					
PTV	48.7 ± 10.8	47.8 ± 10.6	47.6 ± 10.4	−1.7 ± 11.9	0.13
ATV	48.5 ± 10.9	48.0 ± 10.3	47.4 ± 10.7	−1.8 ± 11.5	0.10
Apo A1 (mg/dL)					
PTV	137.0 ± 16.8	NA	136.9 ± 23.7	0.6 ± 14.3	0.45
ATV	136.0 ± 20.7	NA	134.9 ± 17.7	−0.2 ± 9.4	0.79
Apo B (mg/dL)					
PTV	120.0 ± 18.7	NA	88.9 ± 26.0	−26.1 ± 11.9	< 0.001
ATV	121.0 ± 22.5	NA	83.8 ± 22.1	−30.1 ± 14.0	< 0.001

Continuous variables are means ± SD. Apo A1 = apolipoprotein A1; Apo B = apolipoprotein B; NA: not available; for other abbreviations and units, see Table 1. # Values are ([value at 12 weeks − value at baseline]/[value at baseline]).

To compare the lipid-lowering effects of these 2 statins on the DM (n = 125) and non-DM (n = 100) subgroups, we calculated the percentage of patients who achieved target LDL-C levels (< 100 mg/dL) at the end of treatment. The effects were similar for both statins (both p > 0.05) for patients with and without type 2 DM (**Table 3**).

**Table 3:** Percentage of patients who achieved LDL-C < 100 mg/dL after 12 weeks of treatment (stratified by type 2 DM status).

	Type 2 DM^−^			Type 2 DM^+^		
	PTV	ATV		PTV	ATV	p-value
	(n = 50)	(n = 50)	p-value	(n = 62)	(n = 63)	
Achieved LDL-C goal, n (%)	25 (50%)	33 (66%)	0.11	42 (67.8%)	44 (69.8%)	0.8

Type 2 DM^−^ = negative for diabetes mellitus; Type 2 DM^+^ = positive for diabetes mellitus.

Continuous variables are mean ± SD. For other abbreviations and units, see Tables 1 & 2.

Table **4** shows the non-lipid profiles of values and percentage changes from baseline in glucose, insulin, HOMA-IR, fatty acid, HbA _1_C, and ADMA levels (secondary endpoints), and the results of intergroup comparison. Fasting glucose (PTV: +4.8 ± 18.1, p = 0.006; ATV: +4.8 ± 17.0%, p = 0.003), insulin (PTV: +24.8 ± 83.9%, p = 0.002; ATV: +17.9 ± 48.7%, p = 0.002), HOMA-IR (PTV: +33.8 ± 97.4%, p = 0.0004; ATV: +26.5 ± 63.5%, p < 0.0001), ADMA (PTV: +11.2 ± 37.1%, p = 0.002; ATV: +15.1 ± 72.2%, p = 0.03), and fatty acids (PTV: +18.9 ± 99.1%, p = 0.05; ATV: +15.5 ± 80.5%, p = 0.04) were all similarly and significantly higher in both groups. Interestingly, although glucose and insulin levels were higher in both groups, the HbA _1_C level was significantly higher (+1.6 ± 5.2%, p = 0.001) in the ATV group but not in the PTV group (+0.8 ± 7.8%, p = 0.27).

**Table 4:** Non-lipid profiles of values and percentage changes from baseline after 12 weeks of treatment with pitavastatin (PTV) or atorvastatin (ATV).

	Baseline	12 weeks	Percentage change (%)#	Baseline vs. week 12 p-value
Glucose [mg/dL]				
PTV	117.1 ± 26.8	123.0 ± 38.2	4.8 ± 18.1	0.006
ATV	113.5 ± 23.1	119.1 ± 33.3	4.8 ± 17.0	0.003
Insulin [mU/L]				
PTV	13.1 ± 8.2	15.0 ± 11.0	24.8 ± 83.9	0.002
ATV	12.1 ± 7.5	12.7 ± 7.3	17.9 ± 48.7	0.0002
HOMA-IR				
PTV	3.9 ± 3.3	4.9 ± 5.1	33.8 ± 97.4	0.0004
ATV	3.4 ± 2.3	3.8 ± 2.6	26.5 ± 63.5	< 0.0001
Fatty acid [mmol/L]				
PTV	1.02 ± 0.53	1.01 ± 0.7	−18.9 ± 99.1	0.05
ATV	1.04 ± 0.57	0.99 ± 0.59	−15.5 ± 80.5	0.04
HbA_1_C [%]				
PTV	6.5 ± 0.9	6.5 ± 1.1	0.8 ± 7.8	0.27
ATV	6.5 ± 0.8	6.6 ± 1.0	1.6 ± 5.2	0.001
ADMA [μmol/L]				
PTV	0.58 ± 0.13	0.62 ± 0.17	11.2 ± 37.1	0.002
ATV	0.59 ± 0.13	0.65 ± 0.40	15.1 ± 72.2	0.03

Data are means ± standard deviation.

For other abbreviations and units, see Table 1. # Values are expressed as ([value at 12 weeks − value at baseline]/[value at baseline]).

### Safety and Tolerability

Both pitavastatin and atorvastatin were well tolerated, and patients in both groups experienced a similar incidence (PVT: 17 events; AVT: 21 events) of treatment-emergent adverse events (Table **5**). The frequency difference between treatment groups was minor and most of the treatment-related adverse events were moderate or minor. However, there were 7 serious adverse events (SAE) reported in this study. One patient accidentally choked to death on his breakfast at the end of week 7; this event was not related to the medication. One patient had diverticulitis, a moderately severe SAE; this was related to the study medication treatment. The patient was withdrawn from the study and recovered without sequelae. In addition, two patients failed to take 75% of their total study medications: one patient forgot to take 16 capsules on visits 2-5, and another patient forgot to take 8 capsules on visits 3-5.

**Table 5:** Number (%) of patients with adverse events during the 12-week treatment.

	PTV	ATV
	(n = 112)	(n = 113)
	n (%)	n (%)
Adverse drug reaction		
Myalgia	1 (0.9)	2 (1.8)
Back pain	2 (1.8)	3 (2.7)
Skin rash	1 (0.9)	0 (0)
Sleep disorder	0 (0)	0 (0)
Anxiety	1 (0.9)	0 (0)
Upper airway infection	4 (3.5)	6 (5.3)
Nasopharyngitis	6 (5.3)	6 (5.3)
Cough	2 (1.8)	5 (4.4)

Data are n (%). PTV vs. ATV: all p-values were > 0.05. Abbreviations: (see Tables 1 & 2).

Most of the clinical and laboratory safety results analyzed at baseline and after treatment and most of the evaluation results showed minor changes (Table **6**). The hsCRP levels were lower in the PTV group (−28.6 ± 157.4%, p = 0.08) than in the ATV group (−10.8 ± 124.9%, p = 0.38). Although the levels of the parameters were similar before and after treatment between the two treatment groups, the creatine phosphokinase (CPK) (+17.8 ± 45.8%, p = 0.0001), ALT (+10.5 ± 39.9%, p = 0.008), and gamma glutamyl transpeptidase (GT) values (+6.2 ± 32.3%, p = 0.05) were significantly higher in the ATV group, but the change in the PTV group was not significant. More interestingly, the total bilirubin level was unchanged in the PTV group (−0.9 ± 31.8%, p = 0.77) but significantly higher in the ATV group (+14.0 ± 43.0% p = 0.001), which indicated completely different effects on total bilirubin level between the statin treatments (changes by pitavastatin vs. changes by atorvastatin treatment, p < 0.001).

**Table 6:** Safety profiles of values and percentage changes from baseline after 12 weeks of treatment with pitavastatin or atorvastatin.

	Baseline	Week 12	Percentage change (%)#	Baseline vs. week 12 p-value
hsCRP [mg/dL]				
PTV	0.17 ± 0.2	0.14 ± 0.21	−28.6 ± 157.4	0.08
ATV	0.33 ± 1.1	0.19 ± 0.38	−10.8 ± 124.9	0.38
CPK [U/L]				
PTV	125.4 ± 64.6	153.2 ± 252.7	22.3 ± 140.6	0.13
ATV	130.9 ± 81.1	142.7 ± 90.8	17.8 ± 45.8	0.0001
AST [U/L]				
PTV	27.5 ± 10.7	28.3 ± 13.6	5.9 ± 29.3	0.05
ATV	26.8 ± 7.1	27.4 ± 8.5	4.5 ± 24.9	0.07
ALT [U/L]				
PTV	31.5 ± 17.9	32.1 ± 19.5	6.7 ± 33.8	0.06
ATV	30.6 ± 15.1	31.1 ± 14.3	10.5 ± 39.9	0.008
Gamma GT [U/L]				
PTV	37.6 ± 37	34.8 ± 25.6	−5.6 ± 29.9	0.07
ATV	36.8 ± 27.5	37. ± 27.1	6.2 ± 32.3	0.05
Total bilirubin [mg/dL]**				
PTV	0.8 ± 0.3	0.7 ± 0.3	−0.9 ± 31.8	0.77
ATV	0.8 ± 0.3	0.9 ± 0.4	14.0 ± 43.0	0.001
BUN [mg/dL]				
PTV	15.7 ± 3.6	16.4 ± 4.0	5.2 ± 22.0	0.02
ATV	15.7 ± 3.9	16.1 ± 4.5	4.2 ± 19.8	0.03
Serum creatinine [mg/dL]				
PTV	0.8 ± 0.2	0.8 ± 0.2	−1.2 ± 9.9	0.24
ATV	0.8 ± 0.2	0.8 ± 0.2	−0.7 ± 10.7	0.54
LDH [U/L]				
PTV	192.0 ± 31.7	189.7 ± 33.8	−0.3 ± 14.6	0.84
ATV	193.8 ± 38.4	195.3 ± 47.7	1.3 ± 17.9	0.45

Data are means ± standard deviation. Abbreviations and units: (see Tables 1 & 2). # Values are ([value at 12 weeks − value at baseline]/[value at baseline]). ** Values were significantly different between the PTV and ATV groups.

In summary, the differences in clinical and laboratory results between the PTV and ATV groups were mostly nonsignificant. The safety evaluation difference between pitavastatin treatment and atorvastatin treatment was minor.

### Subgroup analysis in DM patients

**Figure 3:**
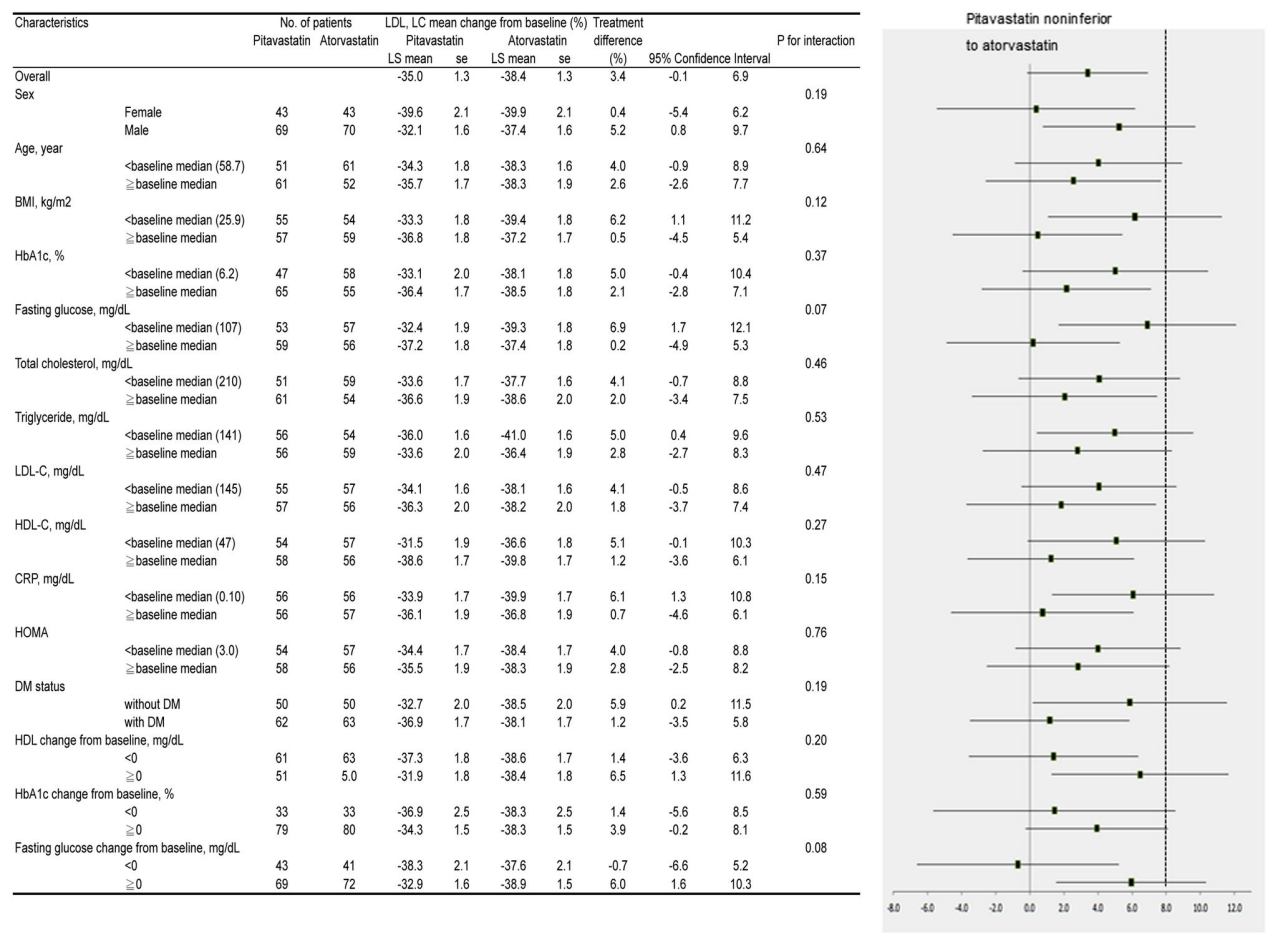
Subgroup analysis comparing the percentage of participants with final LDL-C level < 100 mg/dL after 12 weeks of either pitavastatin (2 mg) or atorvastatin (10 mg) treatment. Continuous variables are means ± SD. ADMA = asymmetric dimethylarginine; ALT = alanine aminotransferase; Apo A1 = apolipoprotein A1; Apo B = apolipoprotein B; AST = aspartate transaminase; BMI = body mass index; BUN = blood urea nitrogen; CPK = creatine phosphokinase; CRP = C-reactive protein; DM = Diabetes mellitus; Gamma GT = gamma-glutamyl transpeptidase; HDL-C = high-density lipoprotein cholesterol; HOMA-IR = homeostatic model assessment-insulin resistance; LDH = lactate dehydrogenase; LDL-C = low-density lipoprotein cholesterol; TG = triglyceride.

Our original aim was to test the comparative effect between the 2 statins in high-risk patients. However, considering the clinical importance and potential impact of type 2 DM on cardiovascular risk, we distributed patients with and without type 2 DM equally during randomization. There was no significant difference in the lipid lowering effect in the groups stratified by type 2 DM status. Finally, a subgroup analysis showed no significant post-treatment differences in LDL-C levels (Figure **3**).

## Discussion

This is the first prospective clinical study that directly compared the efficacy of low-dose pitavastatin and atorvastatin for a high-risk population in Taiwan. Twelve weeks of treatment with pitavastatin (2 mg) or atorvastatin (10 mg) significantly lowered LDL-C, TG, non-HDL-C, and Apo B to a similar extent without serious drug-treatment-emergent adverse effects. Our findings support the clinical utility and universal efficacy of pitavastatin in high-risk patients with hypercholesterolemia, including those with type 2 DM.

### LDL-C lowering effect

The CHIBA [10] study was the first on Japanese patients with metabolic syndrome and a significantly higher-than-normal BMI (mean value: 31.1). In the present study, the average reduction in LDL-C was approximately 35-38% for both the PTV and ATV groups, in patient cohorts at high risk for cardiovascular events, which is comparable to the value reported in the CHIBA study. Furthermore, in a recent meta-analysis [16], there was a trend of LDL-C reduction similar to that shown in our study cohort. The meta-analysis included clinical trials comparing the lipid-profile-lowering effect of standard doses of pitavastatin and atorvastatin. Interestingly, a very similar LDL-C lowering effect was observed (−38 to −44% with pitavastatin (2 mg) and −38 to −45% with atorvastatin (10 mg)). It seems that the LDL-C lowering effects may be similar with either standard dose pitavastatin or atorvastatin universally across different patient cohorts.

### HDL-C modifying effect

One *in vitro* study [17] suggested that, compared with atorvastatin, pitavastatin might increase the production of Apo A1, an essential component of the HDL-C particle, in HepG2 cells at lower concentrations. Moreover, pitavastatin stimulates lipoprotein lipase (LPL) activity more potently than does atorvastatin, which may facilitate an increase in HDL-C through the efficient metabolism of TG-rich lipoproteins [18]. However, in the CIRCLE study [19], there was a significantly different outcome only in patients with lower baseline HDL-C (< 45 mg/dL) levels. The high-risk cohort in the present study had a relatively higher level of baseline HDL-C (48.5 to 48.7 mg/dL), which might partially explain the insignificant difference in the change in the HDL-C level. However, the HDL-C level tended to increase with treatment when the baseline level was < 45 mg/dL. Taken together, it seems that these statins might more effectively raise HDL-C levels in patients with a lower rather than a higher baseline HDL-C level. The above might have potential clinical impact, because the average level of HDL-C is usually < 45 mg/dL in general, and even < 40 mg/dL with type 2 DM in patients in Taiwan with stroke or myocardial infarction [20, 21].

### Glucose metabolic profile

In this high-risk cohort, the glucose metabolism change was particularly intriguing. We found that both statins nonsignificantly increased glucose, insulin, HOMA-IR, and ADMA levels. Interestingly, while unchanged in the PTV group, the level of HbA1c was significantly higher in the ATV group. Our results thus support the notion that these two statins have different therapeutic effects on glucose metabolism. Additional studies are still required to elucidate the mechanisms for the discrepancy in effects on insulin, HOMA-IR, and HbA _1_C. In this regard, some issues may be further mentioned. Firstly, patients with and without type 2 DM were enrolled in the present study. In those with type 2 DM, it could be controversial to evaluate the insulin sensitivity by HOMA-IR and insulin level in the presence of hypoglycemic agents. Secondly, in previous cohort studies [22,23], it took years to evaluate the potential effects of statin treatment on new-onset type 2 DM and deteriorated glucose control. The observation period may be not long enough for consistent changes in glucose metabolism, including both HOMA-IR and HbA _1_C in the current study. Interestingly, pitavastatin increases LPL expression in 3T3-L1 pre-adipocytes in vitro [18]. LPL activity is crucial in VLDL and remnant lipoprotein metabolism, and is often suppressed in the presence of insulin resistance [24]. It was suggested in the CHIBA study that pitavastatin may facilitate remnant lipoprotein catabolism by upregulating LPL activity in Japanese patients with metabolic syndrome [10]. In the current study, we did not investigate the LPL activity. Future long-term effects of pitavastatin on glucose metabolism should be investigated to show their potential clinical impact on this high-risk cohort.

### Safety and tolerability

Throughout this study, both pitavastatin and atorvastatin treatments were well tolerated, and neither of the drugs induced severe adverse reactions, such as rhabdomyolysis, which reflected previous findings that serious adverse events caused by statin treatment are rare. The hsCRP, AST, and gamma GT levels were not significantly different before and after treatment. However, the post-treatment CPK, ALT, and total bilirubin values were all significantly higher in the ATV group but not in the PTV group. During this clinical trial, compared with baseline, the total bilirubin level nonsignificantly decreased in the PTV group but significantly increased in the ATV group. These changes in liver function profiles are partially explained by their metabolic pathways. Pitavastatin and atorvastatin are known to be metabolized via different pathways in the intestine and the liver. In contrast to atorvastatin, which is metabolized primarily by CYP3A4, only a small fraction of pitavastatin is metabolized by CYP2C9 [9]. One study showed that the maximum concentrations in plasma as well as the area under the plasma-concentration curve of atorvastatin were affected by drinking grapefruit juice (a CYP3A4 inhibitor), whereas these parameters for pitavastatin were mostly unaffected [25]. Moreover, when atorvastatin or pitavastatin were added to HepG2 cells at doses that inhibit cholesterol synthesis to comparable levels, the extent of increase in LDL receptor mRNA was significantly larger in the PTV group than in the ATV group [26]. These results suggest that pitavastatin may eliminate cholesterol from blood circulation without excessively hindering cholesterol synthesis in the liver [27].

Limitations of this study are that the treatment period was relatively short (12 weeks) and the sample size was small; therefore, the long-term safety as well as the protective effect of the two statins against cardiovascular diseases could not be evaluated. Trials with more patients and a longer follow-up period are needed to prove the clinical usefulness of pitavastatin.

In conclusion, both pitavastatin (2 mg) and atorvastatin (10 mg) were well tolerated, lowered LDL-C, and improved the lipid profile to a comparable degree in high-risk Taiwanese patients with hypercholesterolemia with and without comorbid type 2 DM.

## Supporting Information

Checklist S1 **CONSORT Checklist.**
(DOC)

Protocol S1 **Trial Protocol.**
(PDF)

Protocol S2 **Trial Registration.**
(PDF)
