# Correction: Pitavastatin and Atorvastatin Double-Blind Randomized ComPArative Study among HiGh-Risk Patients, Including ThOse with Type 2 Diabetes Mellitus, in Taiwan (PAPAGO-T Study)

**DOI:** 10.1371/journal.pone.0114175

**Published:** 2014-11-18

**Authors:** The PLOS ONE Staff

There is an error in affiliation 3 for author Liang-Yu Lin. Affiliation 3 should be: Institute of Pharmacology, National Yang-Ming University and Division of Endocrinology and Metabolism, Internal Medicine, Taipei Veterans General Hospital, Taipei, Taiwan.
