# Supplementary material for: Pitavastatin and Atorvastatin Double-Blind Randomized ComPArative Study among HiGh-Risk Patients, Including ThOse with Type 2 Diabetes Mellitus, in Taiwan (PAPAGO-T Study)
Source: PLoS One. 2013 Oct 1;8(10):e76298. doi: 10.1371/journal.pone.0076298 (PMC3788128; doi:10.1371/journal.pone.0076298)
Supplement: Protocol S1 — Trial Protocol. (PDF) [file pone.0076298.s002.pdf]

Cover Page

Study Title:

A phase IIIb study to compare the efficacy and safety of pitavastatin and atorvastatin: a 12-week, randomized, multicenter, double-blind, active-controlled, non-inferiority study in high risk hypercholesterolemic patients

Study Drug:

Product name: Pitavastatin (Livalo®) vs. Atorvastatin (Lipitor®)
Unit dose: Pitavastatin (Livalo®) 2 mg; Atorvastatin (Lipitor®) 10 mg
Dosage form: Tablet encapsulated for oral administration

Protocol No.: TATPITA20101005

Emergency Contact:

Ms. Yulun Liu, Clinical Research Associate and Ms. Amanda Wu, Project Manager
Virginia Contract Research Organization Co., Ltd.
3F, No. 2, Lane 258, Ruiguang Rd., 114 Taipei, Taiwan
Tel: +886-2-2657-5399 Fax: +886-2-2657-9678

Sponsor Information:

Tai Tien Pharmaceuticals Co., Ltd.
11F, No. 108, Sec. 5, Nanking E. Rd., 105 Taipei, Taiwan
Tel: +886-2-2756-8555 Fax: +886-2-2753-4789

Contract Research Organization (CRO) Information:

Virginia Contract Research Organization Co., Ltd.,
3F, No. 2, Lane 258, Ruiguang Rd., 114 Taipei, Taiwan
Tel: +886-2-2657-5399 Fax: +886-2-2657-9678

Protocol Author(s):

Dr. Jenny Chang, Medical Writer
Virginia Contract Research Organization Co., Ltd.

Compliance of Guidance and/or Rules and Regulations:

This protocol is designed as according to the International conference on harmonization (ICH) guidance and the current Taiwan health authorities' rules and regulations. The study will be conducted in accordance with the design and specific provisions of this Department of Health (DOH) and institutional review boards (IRBs) approved protocol, in accordance with the ethical principles that have their origin in the Declaration of Helsinki, and that are consistent with Good Clinical Practice (GCP) and the applicable regulatory requirement(s).

Confidentiality:

This document contains confidential information. The receiver(s) of this document understood and agreed that this document is not to be disclosed to any unauthorized third party without written agreement issued by the sponsor. The need to disclose certain information contained in this document for the purpose of obtaining informed consent from potential subjects or his/her legally acceptable representative(s) of this study will be considered as exceptions.

Investigator's Signature Page – 1

I have understood the obligations as a clinical trial investigator and agreed to perform and report the study in compliance to the protocol and good clinical practice (GCP) and the current rules and regulations set forth by the applicable health authorities and international conference on harmonization (ICH).

Affiliation	Name of investigator	Position	Signature	Date
Taipei Veterans General Hospital, Taipei, Taiwan	Jaw-Wen Chen	Attending Physician, Department of Medicine, Cardiology	[illegible]	2012-2-17

Investigator's Signature Page – 2

I have understood the obligations as a clinical trial investigator and agreed to perform and report the study in compliance to the protocol and good clinical practice (GCP) and the current rules and regulations set forth by the applicable health authorities and international conference on harmonization (ICH).

Affiliation	Name of investigator	Position	Signature	Date
Taipei Veterans General Hospital, Taipei, Taiwan	Tao-Cheng Wu	Attending Physician, Department of Medicine, Cardiology	Tao-Cheng Wu	Feb. 15. 2012

Investigator's Signature Page – 3

I have understood the obligations as a clinical trial investigator and agreed to perform and report the study in compliance to the protocol and good clinical practice (GCP) and the current rules and regulations set forth by the applicable health authorities and international conference on harmonization (ICH).

Affiliation	Name of investigator	Position	Signature	Date
Taipei Veterans General Hospital, Taipei, Taiwan	Liang-Yu Lin	Attending Physician, Department of Medicine, Endocrinology & Metabolism	Liang-Yu Lin	2012/2/17

Investigator's Signature Page – 4

I have understood the obligations as a clinical trial investigator and agreed to perform and report the study in compliance to the protocol and good clinical practice (GCP) and the current rules and regulations set forth by the applicable health authorities and international conference on harmonization (ICH).

Affiliation	Name of investigator	Position	Signature	Date
Taipei Veterans General Hospital, Taipei, Taiwan	Chin-Chou Huang	Attending Physician, Department of Medicine, Cardiology	Chin-Chou Huang	2012-2-15

Investigator's Signature Page – 5

I have understood the obligations as a clinical trial investigator and agreed to perform and report the study in compliance to the protocol and good clinical practice (GCP) and the current rules and regulations set forth by the applicable health authorities and international conference on harmonization (ICH).

Affiliation	Name of investigator	Position	Signature	Date
Taipei Veterans General Hospital, Taipei, Taiwan	Wen-Chung Yu	Attending Physician, Department of Medicine, Cardiology	Wen-Chung Yu	16-Feb-2012

Investigator's Signature Page – 6

I have understood the obligations as a clinical trial investigator and agreed to perform and report the study in compliance to the protocol and good clinical practice (GCP) and the current rules and regulations set forth by the applicable health authorities and international conference on harmonization (ICH).

Affiliation	Name of investigator	Position	Signature	Date
National Taiwan University Hospital Taipei, Taiwan	Kuo-Liong Chien	Attending Physician, Department of Medicine, Cardiology	[signature]	2012-2-15

Investigator's Signature Page – 7

I have understood the obligations as a clinical trial investigator and agreed to perform and report the study in compliance to the protocol and good clinical practice (GCP) and the current rules and regulations set forth by the applicable health authorities and international conference on harmonization (ICH).

Affiliation	Name of investigator	Position	Signature	Date
National Taiwan University Hospital Taipei, Taiwan	Chia-Lun Chao	Attending Physician, Department of Medicine, Cardiology	Chia-Lun Chao	Feb 15, 2012

Investigator's Signature Page – 8

I have understood the obligations as a clinical trial investigator and agreed to perform and report the study in compliance to the protocol and good clinical practice (GCP) and the current rules and regulations set forth by the applicable health authorities and international conference on harmonization (ICH).

Affiliation	Name of investigator	Position	Signature	Date
National Taiwan University Hospital Taipei, Taiwan	Ta-Chen Su	Attending Physician, Department of Medicine, General Medicine	Ta-Chen Su	2-15-2012

Investigator's Signature Page – 9

I have understood the obligations as a clinical trial investigator and agreed to perform and report the study in compliance to the protocol and good clinical practice (GCP) and the current rules and regulations set forth by the applicable health authorities and international conference on harmonization (ICH).

Affiliation	Name of investigator	Position	Signature	Date
National Taiwan University Hospital Taipei, Taiwan	Hung-Ju Lin	Attending Physician, Department of Medicine, Cardiology	Hung-Ju Lin	2012.2.16

Investigator's Signature Page – 10

I have understood the obligations as a clinical trial investigator and agreed to perform and report the study in compliance to the protocol and good clinical practice (GCP) and the current rules and regulations set forth by the applicable health authorities and international conference on harmonization (ICH).

Affiliation	Name of investigator	Position	Signature	Date
Tri-Service General Hospital, Taipei, Taiwan	Yi-Ren Hung	Attending Physician, Department of Medicine, Endocrinology & Metabolism	[signature]	2012.3.5

Investigator's Signature Page – 11

I have understood the obligations as a clinical trial investigator and agreed to perform and report the study in compliance to the protocol and good clinical practice (GCP) and the current rules and regulations set forth by the applicable health authorities and international conference on harmonization (ICH).

Affiliation	Name of investigator	Position	Signature	Date
Tri-Service General Hospital, Taipei, Taiwan	Chang-Hsun Hsieh	Attending Physician, Department of Medicine, Endocrinology & Metabolism	Chang Hsun Hsieh	2012/03/08

Investigator's Signature Page – 12

I have understood the obligations as a clinical trial investigator and agreed to perform and report the study in compliance to the protocol and good clinical practice (GCP) and the current rules and regulations set forth by the applicable health authorities and international conference on harmonization (ICH).

Affiliation	Name of investigator	Position	Signature	Date
Tri-Service General Hospital, Taipei, Taiwan	Chih-Tsueng He	Attending Physician, Department of Medicine, Endocrinology & Metabolism	Chih-Tsueng He	08 Mar-2012

Investigator's Signature Page – 13

I have understood the obligations as a clinical trial investigator and agreed to perform and report the study in compliance to the protocol and good clinical practice (GCP) and the current rules and regulations set forth by the applicable health authorities and international conference on harmonization (ICH).

Affiliation	Name of investigator	Position	Signature	Date
Tri-Service General Hospital, Taipei, Taiwan	Ling-Yi Wu	Attending Physician, Department of Medicine, Endocrinology & Metabolism	Ling Yi Wu	3/6/2012

Investigator's Signature Page – 14

I have understood the obligations as a clinical trial investigator and agreed to perform and report the study in compliance to the protocol and good clinical practice (GCP) and the current rules and regulations set forth by the applicable health authorities and international conference on harmonization (ICH).

Affiliation	Name of investigator	Position	Signature	Date
Tri-Service General Hospital, Taipei, Taiwan	Fone-Ching Hsiao	Attending Physician, Department of Medicine, Endocrinology & Metabolism	[illegible]	Mar 8. 2012

Investigator's Signature Page – 15

I have understood the obligations as a clinical trial investigator and agreed to perform and report the study in compliance to the protocol and good clinical practice (GCP) and the current rules and regulations set forth by the applicable health authorities and international conference on harmonization (ICH).

Affiliation	Name of investigator	Position	Signature	Date
National Cheng Kung University Hospital, Tainan, Taiwan	Ping-Yen Liu	Attending Physician, Department of Medicine, Cardiology	Ping-Yen Liu	2012-3-5

Investigator's Signature Page – 16

I have understood the obligations as a clinical trial investigator and agreed to perform and report the study in compliance to the protocol and good clinical practice (GCP) and the current rules and regulations set forth by the applicable health authorities and international conference on harmonization (ICH).

Affiliation	Name of investigator	Position	Signature	Date
National Cheng Kung University Hospital, Tainan, Taiwan	Yi-Heng Li	Attending Physician, Department of Medicine, Cardiology	[illegible]	2012. 3 8

Investigator's Signature Page – 17

I have understood the obligations as a clinical trial investigator and agreed to perform and report the study in compliance to the protocol and good clinical practice (GCP) and the current rules and regulations set forth by the applicable health authorities and international conference on harmonization (ICH).

Affiliation	Name of investigator	Position	Signature	Date
National Cheng Kung University Hospital, Tainan, Taiwan	Wei-Chuan Tsai	Attending Physician, Department of Medicine, Cardiology	Wei-Chuan Tsai	15 Mar, 2012

Investigator's Signature Page – 18

I have understood the obligations as a clinical trial investigator and agreed to perform and report the study in compliance to the protocol and good clinical practice (GCP) and the current rules and regulations set forth by the applicable health authorities and international conference on harmonization (ICH).

Affiliation	Name of investigator	Position	Signature	Date
National Cheng Kung University Hospital, Tainan, Taiwan	Ting-Hsing Chao	Attending Physician, Department of Medicine, Cardiology	[signature]	Mar 14. 2012

Investigator's Signature Page – 19

I have understood the obligations as a clinical trial investigator and agreed to perform and report the study in compliance to the protocol and good clinical practice (GCP) and the current rules and regulations set forth by the applicable health authorities and international conference on harmonization (ICH).

Affiliation	Name of investigator	Position	Signature	Date
National Cheng Kung University Hospital, Tainan, Taiwan	Ju-Yi Chen	Attending Physician, Department of Medicine, Cardiology	Ju-Yi Chen	2012/03/05

Investigator's Signature Page – 20

I have understood the obligations as a clinical trial investigator and agreed to perform and report the study in compliance to the protocol and good clinical practice (GCP) and the current rules and regulations set forth by the applicable health authorities and international conference on harmonization (ICH).

Affiliation	Name of investigator	Position	Signature	Date
National Cheng Kung University Hospital, Tainan, Taiwan	Cheng-Han Lee	Attending Physician, Department of Medicine, Cardiology	Cheng-Han Lee	2012-3-8

Investigator's Signature Page – 21

I have understood the obligations as a clinical trial investigator and agreed to perform and report the study in compliance to the protocol and good clinical practice (GCP) and the current rules and regulations set forth by the applicable health authorities and international conference on harmonization (ICH).

Affiliation	Name of investigator	Position	Signature	Date
National Cheng Kung University Hospital, Tainan, Taiwan	Chih-Chan Lin	Attending Physician, Department of Medicine, Cardiology	Chih-Chan Lin	2012/March/07

Investigator's Signature Page – 22

I have understood the obligations as a clinical trial investigator and agreed to perform and report the study in compliance to the protocol and good clinical practice (GCP) and the current rules and regulations set forth by the applicable health authorities and international conference on harmonization (ICH).

Affiliation	Name of investigator	Position	Signature	Date
National Cheng Kung University Hospital, Tainan, Taiwan	Shih-Hung Chan	Attending Physician, Department of Medicine, Cardiology	Shih-Hung Chan	20120309

Investigator's Signature Page – 23

I have understood the obligations as a clinical trial investigator and agreed to perform and report the study in compliance to the protocol and good clinical practice (GCP) and the current rules and regulations set forth by the applicable health authorities and international conference on harmonization (ICH).

Affiliation	Name of investigator	Position	Signature	Date
National Cheng Kung University Hospital, Tainan, Taiwan	Po-Sheng Chen	Attending Physician, Department of Medicine, Cardiology	Po-Sheng Chen	Mar. 9. 2012

Investigator's Signature Page – 24

I have understood the obligations as a clinical trial investigator and agreed to perform and report the study in compliance to the protocol and good clinical practice (GCP) and the current rules and regulations set forth by the applicable health authorities and international conference on harmonization (ICH).

Affiliation	Name of investigator	Position	Signature	Date
National Cheng Kung University Hospital, Tainan, Taiwan	Yen-Wen Liu	Attending Physician, Department of Medicine, Cardiology	Yen-Wen Liu	March 12, 2012

Investigator's Signature Page – 25

I have understood the obligations as a clinical trial investigator and agreed to perform and report the study in compliance to the protocol and good clinical practice (GCP) and the current rules and regulations set forth by the applicable health authorities and international conference on harmonization (ICH).

Affiliation	Name of investigator	Position	Signature	Date
National Cheng Kung University Hospital, Tainan, Taiwan	Wei-Ting Li	Attending Physician, Department of Medicine, Cardiology	Wei-Ting Li	Mar. 8th. 2012

Investigator's Signature Page – 26

I have understood the obligations as a clinical trial investigator and agreed to perform and report the study in compliance to the protocol and good clinical practice (GCP) and the current rules and regulations set forth by the applicable health authorities and international conference on harmonization (ICH).

Affiliation	Name of investigator	Position	Signature	Date
Changhua Christian Hospital, Chang-hua, Taiwan	Chien-Hsun, Hsia	Attending Physician, Department of Medicine, Cardiology	Chien-Hsun Hsia	20120307

Investigator's Signature Page – 27

I have understood the obligations as a clinical trial investigator and agreed to perform and report the study in compliance to the protocol and good clinical practice (GCP) and the current rules and regulations set forth by the applicable health authorities and international conference on harmonization (ICH).

Affiliation	Name of investigator	Position	Signature	Date
Changhua Christian Hospital, Chang-hua, Taiwan	Lee-Shin, Lin	Attending Physician, Department of Medicine, Cardiology	Lee-Shin, Lin	Mar.-14/2012

Investigator's Signature Page – 28

I have understood the obligations as a clinical trial investigator and agreed to perform and report the study in compliance to the protocol and good clinical practice (GCP) and the current rules and regulations set forth by the applicable health authorities and international conference on harmonization (ICH).

Affiliation	Name of investigator	Position	Signature	Date
Mackay Memorial Hospital, Taipei, Taiwan	Hung-I Yeh	Attending Physician, Department of Medicine, Cardiology	Hung-I Yeh	2012, 03, 05

Investigator's Signature Page – 29

I have understood the obligations as a clinical trial investigator and agreed to perform and report the study in compliance to the protocol and good clinical practice (GCP) and the current rules and regulations set forth by the applicable health authorities and international conference on harmonization (ICH).

Affiliation	Name of investigator	Position	Signature	Date
Mackay Memorial Hospital, Taipei, Taiwan	Ta-Chuan Hung	Attending Physician, Department of Medicine, Cardiology	Hung, Ta-Chuan	2012-03-03

Investigator's Signature Page – 30

I have understood the obligations as a clinical trial investigator and agreed to perform and report the study in compliance to the protocol and good clinical practice (GCP) and the current rules and regulations set forth by the applicable health authorities and international conference on harmonization (ICH).

Affiliation	Name of investigator	Position	Signature	Date
Mackay Memorial Hospital, Taipei, Taiwan	Jen-Yuan Kuo	Attending Physician, Department of Medicine, Cardiology	Jen-Yuan Kuo	2012-03-03

Investigator's Signature Page – 31

I have understood the obligations as a clinical trial investigator and agreed to perform and report the study in compliance to the protocol and good clinical practice (GCP) and the current rules and regulations set forth by the applicable health authorities and international conference on harmonization (ICH).

Affiliation	Name of investigator	Position	Signature	Date
Mackay Memorial Hospital, Taipei, Taiwan	Cheng-Huang Su	Attending Physician, Department of Medicine, Cardiology	Cheng-Huang Su	March 3, 2012

Investigator's Signature Page – 32

I have understood the obligations as a clinical trial investigator and agreed to perform and report the study in compliance to the protocol and good clinical practice (GCP) and the current rules and regulations set forth by the applicable health authorities and international conference on harmonization (ICH).

Affiliation	Name of investigator	Position	Signature	Date
Mackay Memorial Hospital, Taipei, Taiwan	Po-Ching Chi	Attending Physician, Department of Medicine, Cardiology	Po-Ching Chi	2012-03-03

Abbreviations and Definition of Terms

AE	Adverse Event
ADA	American Diabetes Association
ADMA	Asymmetric Dimethylarginine
ADR	Adverse Drug Reaction
ALT	Alanine Aminotransferase
AST	Aspartate Aminotransferase
BMI	Body Mass Index
BUN	Blood Urea Nitrogen
CHD	Coronary Heart Disease
CI	Confidence Interval
CIOMS	Council for International Organizations of Medical Sciences
CMH	Cochran-Mantel-Haenszel
CK	Creatinine Kinase
CRA	Clinical Research Associate
CRC	Contract Research Coordinator
CRF	Case Report Form
DHA	Docosahexaenoic Acid
DM	Diabetes Mellitus
EAS	Experimental and Applied Sciences
EC	Ethics Committee
EKG	Electrocardiogram
EPA	Eicosapentaenoic Acid
FDA	Food and Drug Administration
FPG	Fasting Plasma Glucose
GCP	Good Clinical Practice
Hb	Hemoglobin
HbA1c	Glycosylated Hemoglobin
Hct	Hemotocrit
HDL	High-Density Lipoprotein
HMG-CoA	Hydroxy-Methylglutaryl Coenzyme-A

IB	Investigator Brochure
IP	Investigational Product
IRB	Institutional Review Board
IVR	Interactive Voice Response
LDL	Low-Density Lipoprotein
LOCF	Last Observation Carried Forward
MedDRA	Medical Dictionary for Regulatory Activities
MHRA	Medicines and Healthcare products Regulatory Agency
NCEP ATP III	National Cholesterol Education Program Adult Treatment Panel III
NPC1L1	Niemann-Pick disease, type C1, gene Like 1
NYHA	New York Heart Association
OHA	Oral Hypoglycemic Agents
OPD	Out Patient Department/clinic
QD (q.d)	Quaque die (every day)
RBC	Red Blood Cell
RDI	Recommended Daily Intake
SAP	Statistical Analysis Plan
SSRIs	Selective Serotonin Reuptake Inhibitors
SUSAR	Suspected Unexpected Serious Adverse Reaction
TC	Total Cholesterol
TCAs	Tricyclic Antidepressants
TG	Triglyceride
UCL	Union Clinical Laboratory
UK	United Kingdom
ULN	Upper Limit of Normal
ULRR	Upper Limit of the Reference Range
VCRO	Virginia Contract Research Organization Co., Ltd.
VCROIVRS	VRCO Interactive Voice Response System
WBC	White Blood Cell

Table of Contents

COVER PAGE 1
INTERNAL SIGNATURE PAGE 2
INVESTIGATOR'S SIGNATURE PAGE – 1 1
INVESTIGATOR'S SIGNATURE PAGE – 2 2
INVESTIGATOR'S SIGNATURE PAGE – 3 3
INVESTIGATOR'S SIGNATURE PAGE – 4 4
INVESTIGATOR'S SIGNATURE PAGE – 5 5
INVESTIGATOR'S SIGNATURE PAGE – 6 6
INVESTIGATOR'S SIGNATURE PAGE – 7 7
INVESTIGATOR'S SIGNATURE PAGE – 8 8
INVESTIGATOR'S SIGNATURE PAGE – 9 9
INVESTIGATOR'S SIGNATURE PAGE – 10 10
INVESTIGATOR'S SIGNATURE PAGE – 11 11
INVESTIGATOR'S SIGNATURE PAGE – 12 12
INVESTIGATOR'S SIGNATURE PAGE – 13 13
INVESTIGATOR'S SIGNATURE PAGE – 14 14
INVESTIGATOR'S SIGNATURE PAGE – 15 15
INVESTIGATOR'S SIGNATURE PAGE – 16 16
INVESTIGATOR'S SIGNATURE PAGE – 17 17
INVESTIGATOR'S SIGNATURE PAGE – 18 18
INVESTIGATOR'S SIGNATURE PAGE – 19 19
INVESTIGATOR'S SIGNATURE PAGE – 20 20
INVESTIGATOR'S SIGNATURE PAGE – 21 21
INVESTIGATOR'S SIGNATURE PAGE – 22 22
INVESTIGATOR'S SIGNATURE PAGE – 23 23
INVESTIGATOR'S SIGNATURE PAGE – 24 24
INVESTIGATOR'S SIGNATURE PAGE – 25 25
INVESTIGATOR'S SIGNATURE PAGE – 26 26
INVESTIGATOR'S SIGNATURE PAGE – 27 27
INVESTIGATOR'S SIGNATURE PAGE – 28 28
INVESTIGATOR'S SIGNATURE PAGE – 29 29
INVESTIGATOR'S SIGNATURE PAGE – 30 30
INVESTIGATOR'S SIGNATURE PAGE – 31 31
INVESTIGATOR'S SIGNATURE PAGE – 32 32
ABBREVIATIONS AND DEFINITION OF TERMS 4
TABLE OF CONTENTS 6
1. PROTOCOL SYNOPSIS 9

1.1 SYNOPSIS ... 9
SAMPLE SIZE DETERMINATION: ... 18
1.2 FLOW CHART ... 20
1.2.1 Clinical Flow Chart ... 20

2. INTRODUCTION ... 23

2.1 DISEASE BACKGROUND ... 23
2.2 INVESTIGATIONAL PRODUCT ... 24
2.3 STUDY RATIONALE ... 25

3. STUDY OBJECTIVES AND ENDPOINTS ... 26

3.1 STUDY OBJECTIVES ... 26
3.1.1 Primary Study Objective ... 26
3.1.2 Secondary Study Objectives ... 26
3.2 ENDPOINTS ... 26
3.2.1 Primary Endpoints ... 26
3.2.2 Secondary Endpoints ... 26

4. STUDY DESIGN ... 29

4.1 TYPE AND DESIGN ... 29
4.1.1 Sample Size ... 29
4.1.2 Choice of Control ... 29
4.2 AVOID/MINIMIZE BIAS ... 30
4.3 DOSE RATIONALE ... 30
4.4 RATIONALE OF FOOD INTAKE RELATED TO IP ADMINISTRATION ... 30

5. STUDY POPULATION ... 32

5.1 NUMBER OF PATIENTS ... 32
5.2 ELIGIBILITY CRITERIA ... 32
5.2.1 Inclusion Criteria ... 32
5.2.2 Exclusion Criteria ... 32
5.2.3 Withdrawal Criteria ... 33

6. STUDY ASSESSMENT AND PROCEDURES ... 35

6.1 STUDY ASSESSMENT ... 35
6.1.1 Informed Consent ... 35
6.1.2 Eligibility ... 35
6.1.3 Height ... 35
6.1.4 Demographics ... 35
6.1.5 Medical History ... 35
6.1.6 Concurrent disease/status ... 35
6.1.7 Urine Pregnancy Test ... 35
6.1.8 Vital Signs and body weight ... 36
6.1.9 Framingham projection ... 36
6.1.10 Physical Examinations ... 36
6.1.11 Laboratory Tests ... 36
6.1.12 HOMA-IR ... 37
6.1.13 Primary efficacy endpoint ... 37
6.1.14 Secondary efficacy endpoint ... 37
6.1.15 Patient Identifier ... 38
6.1.16 Previous and Concomitant Medications ... 38
6.1.17 Investigational Drug ... 38
6.1.18 Adverse Event (AE) ... 38
6.1.19 EKG ... 39
6.1.20 Exit of Study ... 39
6.2 RANDOMIZATION & BLINDING PROCESS MANAGEMENT ... 39
6.2.1 Visit 1 ... 39
6.2.2 Visit 2 ... 42

6.2.3 Visit 3 42
6.2.4 Visit 4 43
6.2.5 Visit 5 44

7. INVESTIGATIONAL PRODUCT 46

7.1 DESCRIPTION OF INVESTIGATIONAL PRODUCT AND THE COMPARATOR DRUG 46
7.2 DOSAGE AND ADMINISTRATION 46
7.3 DOSE RATIONALE 47
7.4 TREATMENT ASSIGNMENT 47
7.5 PACKAGING AND LABELING 47
7.6 HANDLING AND STORAGE 48
7.7 PRODUCT ACCOUNTABILITY 48
7.8 ASSESSMENT OF COMPLIANCE 48
7.9 TREATMENT FOR INVESTIGATIONAL PRODUCT OVERDOSE 49

8. CONCOMITANT TREATMENTS 50

8.1 PERMITTED TREATMENTS 50
8.2 PROHIBITED TREATMENTS 50

9. ADVERSE EVENTS (AE) AND SERIOUS ADVERSE EVENTS (SAE) 52

9.1 DEFINITION OF AN AE 52
9.2 DEFINITION OF AN SAE 52
9.3 DISEASE-RELATED EVENTS 52
9.4 LACK OF EFFICACY 53
9.5 CLINICAL LABORATORY ABNORMALITIES 53
9.6 REPORTING AND RECORDING OF AE AND SAE 53

10. STATISTICAL CONSIDERATIONS AND STATISTICAL ANALYSIS 56

10.1 SAMPLE SIZE CALCULATION 56
10.2 POPULATION TO BE ANALYZED 57
10.3 BACKGROUND AND DEMOGRAPHIC CHARACTERISTICS 57
10.4 EFFICACY PARAMETERS 57
10.4.1 Primary Endpoint 57
10.4.2 Secondary Endpoints 58
10.5 CONCOMITANT THERAPY AND MEDICAL HISTORY 60
10.5.1 Compliance/Extend of Exposure 60
10.5.2 Multi-Center Study 60
10.5.3 Missing Values 60
10.5.4 Interim Analysis 60

11 STUDY ADMINISTRATIVE STRUCTURE 61

11.1 INFORMED CONSENT AND INSTITUTIONAL REVIEW 61
11.2 CONFIDENTIALITY/PUBLICATION OF THE STUDY 61
11.3 RECORD KEEPING 61
11.3.1 Data Collection 61
11.3.2 Drug Accountability 62
11.3.3 Record Retention 62

12 REFERENCES 63

13 APPENDICES 65

13.1 DECLARATION OF HELSINKI 65
13.2 ATP III GUIDELINES 70
13.3 MEDICATIONS MAY INTERFERE WITH IP 76
13.4 NATURAL HEALTH FOOD PRODUCTS MAY INTERFERE WITH IP 78

1. Protocol Synopsis

1.1 Synopsis

**Study Title**	A phase IIIb study to compare the efficacy and safety of pitavastatin and atorvastatin: a 12-week, randomized, multicenter, double-blind, active-controlled, non-inferiority study in high risk hypercholesterolemic patients
**Study Phase**	IIIb
**Investigational Product (IP)**	Generic name: Pitavastatin<br>Product name: Livalo®<br>Unit dose: Pitavastatin (Livalo®) 2 mg<br>Atorvastatin (Lipitor®) 10 mg<br>Dosage form: Tablet encapsulated for oral administration
**Active Ingredient**	Pitavastatin calcium
**Route of Administration**	Oral administration
**Study Objective(s)**	The primary study objective is to compare pitavastatin 2 mg versus atorvastatin 10 mg given once daily in the low-density lipoprotein cholesterol (LDL-C) lowering effect in patients with hypercholesterolemia.
**Diagnosis and Main Patient Inclusion Criteria**	1. Patient aged ≥ 20 years old and < 75 years old.<br>2. Patient who was eligible and able to participate in the study and accepts to enter the study by signing written informed consent.<br>3. Patient with fasting LDL-C > 100 mg/dL. The concentration of LDL-C is obtained from laboratory examination.<br>4. Patient with at least one of the following description (NCEP ATP III guideline):<br>➢ Documented coronary heart disease (CHD)<br>➢ Type 2 diabetes mellitus (DM)<br>➢ If below 2+ risk factors (other than LDL) are present without CHD or CHD risk equivalent, assess 10-year (short-term) CHD risk (see Framingham score) > 20%.<br>✓ Female age ≥ 55 years old and male age ≥ 45 years old.<br>✓ Fasting high-density lipoprotein cholesterol (HDL-C) < 40mg/dL.<br>✓ Patient who has family history of premature CHD (CHD in male first degree relative < 55 years; CHD in female first degree relative < 65 years).

	✓ Hypertension (BP ≥ 140/90 mmHg or treated with anti-hypertensive agents).<br>✓ HDL-C ≥ 60 mg/dL count as "negative" risk factor; its presence removes one risk factor from the total count.<br>5. Female patient with child-bearing potential must take reliable contraception method(s) during the participation of the study.
**Patient Exclusion Criteria**	1. Patient who has participated in other investigational studies within 3 months before Visit 2.<br>2. Patient took medication and natural health foods (see **Appendix 13.3** and **13.4**) known to alter blood lipid profiles within 4 weeks of Visit 2.<br>3. Patient is taking any medication or food (see **Section 8.2**) that is prohibited by the study.<br>4. Patient taking Amiodarone will be excluded from this study (due to long half life of this medication).<br>5. Patient is diagnosed with type 1 DM or has been using insulin/insulin analog medication.<br>6. Patient with a history of multiple drug allergies or with a known allergy to hydroxy-methylglutaryl coenzyme-A (HMG-CoA) reductase inhibitors.<br>7. Patient with triglyceride (TG) > 400 mg/dL.<br>8. Excessive obesity defined as body mass index (BMI) above 35 kg/m^2.<br>9. Cerebral vascular disease (including cerebrovascular hemorrhage or ischemia, transient ischemic attack) diagnosed within 3 months before Visit 2.<br>10. Myocardial infarction, heart failure (NYHA class III or IV), gross cardiac enlargement (cardiothoracic ratio > 0.5), significant heart block or cardiac arrhythmias within 3 months before Visit 2; history of uncontrolled complex ventricular arrhythmias, uncontrolled artrial fibrillation/flutter or uncontrolled supraventricular tachycardia, pacemaker or implantable cardiac device were not eligible for this study.<br>11. Patient with advanced renal disorder (Serum creatinine levels ≥ 2 mg/dL and blood urea nitrogen (BUN) ≥ 25 mg/dL) at Visit 1.<br>12. Patient with advanced hepatic disorder (aspartate aminotransferase (AST) or alanine aminotransferase (ALT) level ≥ 100 IU/L).<br>13. Patient with creatine kinase (CK) level > 5 × ULRR at

any time point between Visit 1 and Visit 2.

14. Patient with poorly controlled diabetes mellitus (HbA1c > 9.0%) or patient with severe hypertension (> 180 mmHg for systolic or > 120 mmHg in diastolic blood pressure).
15. Patient with hypothyroidism, hereditary muscular disorders, family history of the above or history of drug-induced myopathy.
16. Patient has significant alcohol consumption (> 65 mL pure alcohol) within 48 hours before Visit 2.
17. Any major surgery within 3 months prior to Visit 2.
18. Female patient who is lactating, being pregnant or plans to become pregnant.
19. Patient with conditions judged by the investigator as unsuitable for the study.

**Study Structure**	**Study design and plan:**<br>This is a randomized, double-blind, active-controlled, non-inferiority study carrying in a multi-center setting. The study is designed for evaluating lipid lowering effects of Pitavastatin as a new HMG-CoA reductase inhibitor drug.<br><br>Patients will be recruited for at least a total of 144 evaluable cases. The total randomized patient number in this study is estimate of about 226 cases.<br><br>At Visit 1, patients will require completing designed assessments and examinations, including physical examinations, demographics, medical history, medication history, vital signs, EKG, and clinical laboratory tests to evaluate and recruit the possible candidates. The Visit 1 may involve up to 14 days before Visit 2. Patients are base on Visit 1 examinations to justify their eligibility to enroll in the study. All the laboratory test results obtained from Visit 1 will be used for baseline calculation. On Visit 2, recruited patients will be asked to complete required assessments. No additional performance for laboratory tests is needed in Visit 2. However, when investigator has any concern about the patient's condition, an extra performance to withdraw blood for required laboratory tests is allowed. Additional examinations obtained from Visit 2 will be used as safety purposes. This is an optional re-check point for investigators to assure patient's eligibility and safety for this study. Furthermore, if the patient has undergone an additional laboratory test on Visit 2, the test results obtained from the Visit 2 will be used as the baseline calculation, combining with other laboratory tests obtained from Visit 1. Study medications will be dispensed to the randomized patients at Visit 2, Visit 3 and Visit 4 by a 4-weekly basis. Visit 3 and Visit 4 will be carried on week 4 (28±5 days) and week 8 (56±5 days). If patients have an additional laboratory tests at Visit 2, study medications will be dispensed after rechecking the laboratory test result is acceptable for eligibility (approximate 1 week after Visit 2). The laboratory tests for safety and efficacy evaluation will be performed on Visit 3 (week 4, 28±5 days). There is no necessary to carry another laboratory tests on Visit 4 (week 8, 56±5 days), unless an additional concern has been made by the investigator. The Visit 5 will be on week 12 (84±5 days). The concomitant medications, dispense study drug, laboratory exams, the collection of unused treatment medications, and adverse event will be performed/recorded according to clinical flow chart (See **1.2.1 Clinical Flow Chart** for detailed information).
**Study Structure (continuous)**	There will be no change in study drug doses across the entire study duration. The study drug is dispensed according to the treatment schedule in the study protocol, by a 4-weekly basis. Patients are dosed once daily in the evening after dinner.

**Duration of study**	Patients will undergo screening procedures at Visit 1 for determining eligibility within 14 days prior to randomizing the patients (Visit 2). Patients will be dispensed of study medications from out patient department/clinic (OPD) by the investigators starting from Visit 2 (Day 0) until Visit 5. Patients are required to come back for Visit 3 and Visit 4 on week-4 and week-8, and trial closes on the Visit 5, which is on week-12. During this 12 weeks study, patients may be withdrawn from the study when meeting at least one of the withdrawal criteria.

**Center**	**North Taiwan:**<br>National Taiwan University Hospital, Taipei, Taiwan<br>Taipei Veterans General Hospital, Taipei, Taiwan<br>Tri-Service General Hospital, Taipei, Taiwan<br>Mackay Memorial Hospital, Taipei, Taiwan<br>**Central Taiwan:**<br>Changhua Christian Hospital, Changhua, Taiwan<br>**Southern Taiwan:**<br>National Cheng Kung University Hospital, Tainan, Taiwan
**Blinding**	Double
**Study Arm**	Pitavastatin 2 mg, tablet encapsulated<br>Atorvastatin 10 mg, tablet encapsulated
**Stratification**	A marked deterioration of glycemic control has been reported in some patients treated with atorvastatin. Therefore, this stratification of DM and non-DM groups is use to compare the effects of pitavastatin with atrorvastatin on glycemic control.<br>Type 2 DM with at least one of the following description: fasting glucose > 126 mg/dL, taking OHA, HbA1c ≥ 6.5%, and random plasma glucose ≥ 200 mg/dL twice in a row according to ADA criteria.<br>Group that did not include any mentioned-above criteria will be stratified to non-DM group.
**Randomization**	Yes
**Patient Sample Size**	Approximate 226 patients will be randomized to achieve at least 144 evaluable patients
**Study Assignment**	Patients who meet all eligible requirements for entry into the study will be randomized into 1 of the 2 treatment groups in 1:1 ratio as shown below:<br>1. Pitavastatin 2 mg / capsule, given once daily after dinner in the evening<br>2. Atorvastatin 10 mg / capsule, given once daily after dinner in the evening
**Primary Endpoints**	The mean percentage change of the week-12 (Visit 5) evaluation compared with the baseline on the LDL-C blood levels of the patients between pitavastatin and atorvastatin.
**Secondary Endpoints**	**Efficacy**<br>**Lipid efficacy**<br>1. To estimate the proportion of patients achieving LDL-C blood

level to < 100 mg/dL at the week-12 (Visit 5).

2. The mean percentage change of LDL-C blood level comparing baseline to week-4 (Visit 3).
3. The mean percentage changes of HDL-C blood level comparing baseline to week-4 (Visit 3) and week-12 (Visit 5).
4. The mean percentage changes of TG blood level comparing baseline to week-4 (Visit 3) and week-12 (Visit 5).
5. The mean percentage changes of non-HDL-C blood level comparing baseline to week-4 (Visit 3) and week-12 (Visit 5) (non-HDL-C = Total Cholesterol – HDL-C).
6. To evaluate the net changes of Apolipoprotein A1 (Apo A1) and Apolipoprotein B (Apo B) comparing baseline to the week-12 (Visit 5).

Non-lipid efficacy

1. To evaluate the net changes of fasting plasma glucose from the baseline to the week-4 (Visit 3) and week-12 (Visit 5).
2. To evaluate the net changes of fasting insulin level from the baseline to the week-12 (Visit 5).
3. To evaluate the baseline and week-12 (Visit 5) insulin resistance by the homeostasis model assessment method (HOMA-IR), in equation of:

$$\text{HOMA-IR} = \frac{\text{fasting insulin level } (\mu\text{U/ml}) \times \text{fasting plasma glucose (mg/dL)}}{405 \text{ (mmol/L)}}$$

4. To evaluate the net change of HbA1c comparing the baseline to the week-12 (Visit 5).
5. To evaluate the net change of free fatty acid comparing the baseline to the week-12 (Visit 5).
6. To evaluate the net change of asymmetric dimethylarginine (ADMA) from the baseline to the week-12 (Visit 5).

Note: all efficacy endpoints, including primary endpoint, will be statistically analyzed on DM and non-DM groups, respectively, as well as pool data.

Safety

Clinical safety

1. To evaluate the changes in physical examination results.
2. To evaluate the changes in vital signs (pulse, systolic and diastolic blood pressures).
3. To evaluate the changes in urinalysis, including pH, erythrocyte, leukocyte, glucose, and protein (qualitatively).
4. To evaluate the change of EKG results (including PR, QRS, QT,

	QTc, RR intervals).<br>**Laboratory safety**<br>To evaluate the net change of safety profile from baseline to each visit according to the flow chart (See **1.2.1 Clinical Flow Chart**) by the follow laboratory tests:<br>- White blood cell (WBC) with differential counts<br>- Red blood cell (RBC)<br>- Hemoglobin (Hb)<br>- Hematocrit (Hct)<br>- Platelet (PLT)<br>- C-reactive protein (CRP)<br>- Creatinine kinase (CK)<br>- Aspartate aminotransferase (AST)<br>- Alanine aminotransferase (ALT)<br>- γ-Glutamyl transpeptidase (GGT)<br>- Total bilirubin<br>- BUN<br>- Serum creatinine<br>- Lactate dehydrogenase (LDH)<br>**Adverse event (AE)**<br>To evaluate AEs incidences.
**Schedule of Visit**	A total of 5 visits will be performed for this study, including Visit 1 (≤ 14 days prior to Visit 2), Visit 2 on day 0, Visit 3 on day 28±5, Visit 4 on day 56±5, and Visit 5 on day 84±5.
**Statistical Analysis**	**Populations for analysis:**<br>Patients will be categorized into the following populations to meet various study purposes.<br>Intention-to-treat (ITT) population: the ITT population will comprise all patients who ever received the trial medication.<br>Per-protocol (PP) population: the PP population is a subset of ITT who satisfy the following conditions.<br>1. Fulfilling all inclusion and exclusion criteria<br>2. Dosed with at least 75% of total study medications (= 63 capsules)<br>3. With primary efficacy measurement<br>4. Not taking any prohibited medications

Safety evaluations will be performed on ITT population while efficacy analysis will be performed on ITT and PP populations. The conclusion of efficacy of the study will be made according to the results of ITT analysis.

Statistical analysis:

Summary statistics will be provided for all efficacy, safety, and baseline/demographic variables by treatment, center and DM status (2 categories only: diabetic and non-diabetic). For categorical variables, frequency tables including percentages will be presented. For continuous variables, descriptive statistics such as number of available observations, mean, median, standard deviation (SD), inter-quartile range (IQR), minimum, and maximum will be tabulated.

All statistical tests will be two-tailed with α=0.05.

Mean percentage change of LDL-C, HDL-C, TG and non-HDL-C will be analyzed by using ANCOVA incorporating treatment, DM status and risk factors defined in inclusion criteria 4 (if appropriate, including condition of existing CHD and other risk factors to be determined in statistical analysis plan (SAP)) as factors and the baseline value as covariate. The proportion of patients achieving a target of lowering LDL-C will be analyzed by using CMH (Cochran-Mantel-Haenszel) test incorporating DM status as stratifications.

The hypothesis on primary endpoint will be

H_0: $\mu_1 - \mu_2 \leq \delta$

H_1: $\mu_1 - \mu_2 > \delta$, δ= -8.0%

μ_1 and μ_2 denotes mean % change of Pitavastatin 2 mg QD and Atorvastatin 10 mg QD, respectively, from baseline to week 12 (Visit 5). Treatment group will be declared non-inferiority if the lower limit of the 95% two-sided confidence interval of the adjust means between treatment difference is greater than -8.0%.

Changes in Apo A1, Apo B, free plasma glucose, insulin level, HOMA-IR, HbA1c, free fatty acid, ADMA will be analyzed by using ANCOVA incorporating treatment and DM status and risk factors defined in inclusion criteria 4 (if appropriate, to be determined in SAP) as factors and the baseline value as covariate.

Subgroup analysis of all efficacy endpoints will be also performed on DM and non-DM groups, respectively.

Changes in vital signs, EKG (including PR, QRS, QT, QTc, RR intervals), CBC tests, biochemistry tests will be analyzed by using ANCOVA incorporating treatment and DM status as factors and the baseline value as covariate. Urinalysis will be presented with descriptive statistics to demonstrate the trend of change.

Number of patients with physical exam abnormality at each scheduled visit will be tabulated by body system. EKG examination results will be displayed in descriptive statistics with number of patients with abnormal findings tabulated for each schedule visit.

Adverse event incidents will be summarized descriptively by system organ class and preferred term using the MedDRA and by treatment group. The severity grade, relationship, action taken and outcome will also be analyzed.

Demography and baseline characteristics including gender, age, height, weight and BMI will be listed by treatment group and analyzed between treatments to ensure comparability by using appropriate statistical methods of either ANOVA or other non-parametric tests incorporating treatment and DM status as factors for continuous variables or using appropriate statistical methods of CMH (Cochran-Mantel-Haenszel) test incorporating DM status as stratifications for categorical variables.

Sample Size Determination:

Statistical justification for sample size is primarily based on the study results obtained from Phase III NK-104-301 study (see Reference 18, Study of pitavastatin 2 mg vs. atorvastatin 10 mg and pitavastatin 4 mg vs. atorvastatin 20 mg (following up-titration) in patients with primary hypercholesterolemia or combined dyslipidemia (protocol No.: NK-104-301). The primary endpoint utilized for this cited study is the same as the one used in this proposed study, namely, the mean percentage change of the 12th week evaluation compared to the baseline on the LDL-C blood levels of the patient. The following contents are the main statistical basis for estimating sample size for this proposed study:

The mean percentage change of the 12th week evaluation compared to the baseline on the LDL-C blood levels of the patients.

1. Mean percentage change of the 12th week evaluation compared to the baseline on the LDL-C blood levels of following 2 arms based on NK-104-301 study:
   - ✓ Pitavastatin 2 mg QD
   - ✓ Atorvastatin 10 mg QD
2. Null and alternate hypotheses:

   H_0: $\mu_1 - \mu_2 \leq \delta$

   H_1: $\mu_1 - \mu_2 > \delta$, δ= -8.0%

   μ_1 and μ_2 denotes mean % change of Pitavastatin 2 mg QD and Atorvastatin 10 mg QD, respectively, from baseline to week 12, estimated as 37.9% (SD=13.97%) and 37.8% (SD=15.60%) in the NK-104-301 study.

3. Type I error rate: 0.05
4. Statistical power: 90%
5. Population for primary analysis in NK-104-301 study: FAS (full analysis population) population, defined as all randomized patients who receive at least one dose of study drug and who had at least one on-treatment lipid assessment. The FAS population is the same as ITT defined in this study for the primary endpoint.
7. Statistical method used to calculate the sample size: two group Satterthwaite t-test of equal means (unequal variances) by using nQuery Advisor 7.0.
8. Interim analysis: None
9. Treatment group ratio: 1 to 1 for pitavastatin 2 mg QD versus atorvastatin 10 mg QD

Sample size derived from the above mentioned statistical conditions is 72 for each group. The evaluable patients will be determined as ITT patients with at least 1 post-treatment LDL-C measurement.

1.2 Flow Chart

1.2.1 Clinical Flow Chart

**Visit**	**1**	**2**	**3**	**4**	**5**	
**Week**	≤ -2	0	4	8	12	
**Day**	≤ -14	0	28±5	56±5	84±5	
Informed consent signed and given	√					6.1.1
Inclusion and exclusion criteria	√	√[4]				6.1.2
Height	√					6.1.3
Demographic information	√					6.1.4
Medical History (including smoking history)	√					6.1.5
Urine pregnancy test for applicable patients[1]	√	√	√	√	√	6.1.7
Vital signs and body weight	√	√	√	√	√	6.1.8
Concurrent diseases/ status	√	√	√	√	√	6.1.6
Framingham risk score	√					6.1.9
Physical examinations	√	√			√	6.1.10
EKG examinations	√[2]				√	6.1.19
Randomization number assigned by IVRS		√				6.1.15
Hematology<br>a. White blood cells with differential counts<br>b. Red blood cells<br>c. Hemoglobin<br>d. Hematocrit<br>e. Platelet	√[3]				√	6.1.11
Safety evaluations<br>a. CRP<br>b. CK<br>c. AST<br>d. ALT<br>e. GGT	√[3]	√[4]	√	√[4]	√	6.1.11
Safety evaluations<br>a. Total bilirubin<br>b. BUN<br>c. Serum creatinine<br>d. LDH	√[3]				√	6.1.11
Urinalysis<br>a. pH<br>b. RBC<br>c. WBC<br>d. Glucose<br>e. protein	√[3]	√[4]			√	6.1.11
Fasting insulin level	√[3]				√	6.1.11
Fasting plasma glucose	√[3]	√[4]	√	√[4]	√	6.1.11
HOMA-IR	√[3]				√	6.1.12
HbA_{1c}	√[3]				√	6.1.11
Fasting plasma lipids<br>a. Total cholesterol (TC)<br>b. Triglycerides (TG)<br>c. LDL-cholesterol (LDL-C)<br>d. HDL-cholesterol (HDL-C)	√[3]	√[4]	√	√[4]	√	6.1.11

Apo A1 and Apo B[2]	√[3]				√	6.1.11
Fatty acid<br>ADMA	√[3]				√	6.1.11
Dispense study drug		√[5]	√	√		6.1.17
Unused treatment medication collection			√	√	√	6.1.17
Record concomitant medications	√	√	√	√	√	6.1.16
Record adverse event (AE)		√	√	√	√	6.1.18
Complete exit form					√	6.1.20
Dismiss patient					√	6.1.20

1. The applicable patients for urine pregnancy test stand for female patient with childbearing potential.
2. Test results within 30 days before Visit 1 are acceptable.
3. All the laboratory test results obtained from Visit 1 will be used for baseline calculation. If the patient has undergone an additional laboratory test on Visit 2, the test results obtained from Visit 2 will be used as the baseline calculation, combining with other laboratory tests obtained from Visit 1.
4. When investigator has any concern about the patient's condition, an optional performance to withdraw blood for required laboratory tests is allowed.
5. If patients have an additional laboratory tests at Visit 2, study medications will be dispensed after rechecking the laboratory test result is acceptable for eligibility (approximate 1 week after Visit 2).

Clinical Schematic Diagram

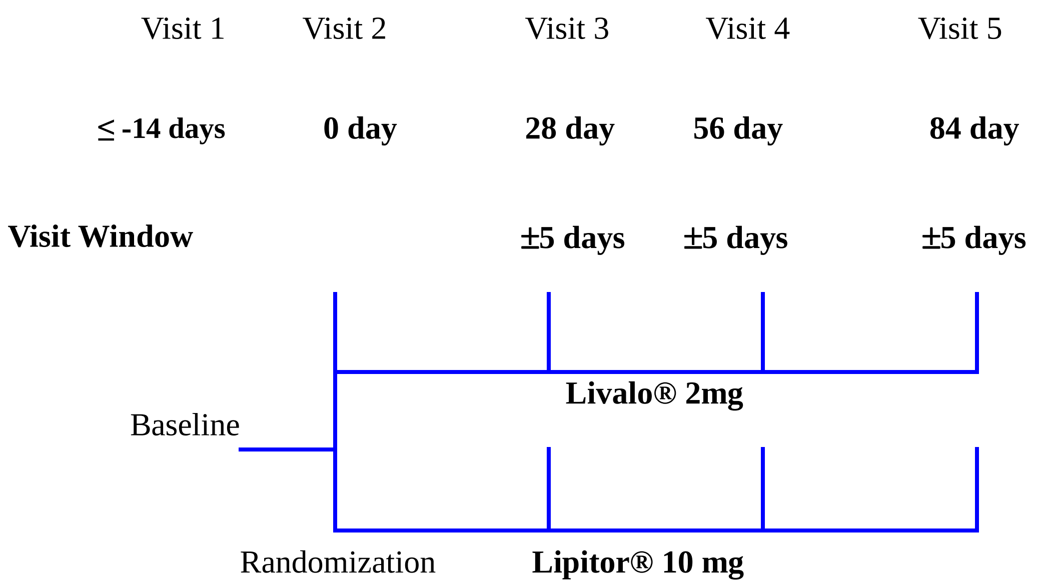

2. Introduction

2.1 Disease Background

Atherosclerosis and its complications are the leading cause of mortality amongst men and women worldwide. The prevalence in developing countries is increasing as they adopt the eating, smoking and more sedentary habits in modern life styles in Northern American, European and Asia societies [1].

The pathogenesis of atherosclerosis is a chronic state of dyslipoproteinaemia and inflammation, which encourages cholesterol plaque formation within arterial vessel walls, exacerbated by hypertension, tobacco use and diabetes. The process is mediated through a variety of lipoprotein subclasses including low density lipoprotein (LDL) and very low density lipoprotein (VLDL), which transport cholesterol and triglycerides to peripheral tissues and are responsible for the formation of the lipid-rich, unstable atheromatous plaques that are the basis of atherosclerosis. High density lipoprotein (HDL) is involved in transporting cholesterol from cholesterol replete peripheral tissues back to the liver and is inversely linked to cardiovascular risk through its anti-inflammatory, antioxidative and antithrombotic effect on vasculature [2]. Studies have confirmed that lowering LDL, VLDL and VLDL remnants significantly improves the risks for cardiovascular disease and have demonstrated the protective benefits of HDL.

The National Cholesterol Education Program Adult Treatment Panel III (NCEP ATP III) recommendations provide guidance on the initiation of treatment aimed at lowering lipid levels based on individual patient characteristics [3]. Three levels of risk have been established, with the highest risk individuals being those with coronary heart disease (CHD), diabetes, clinical atherosclerotic disease in other vascular beds, or multiple risk factors, resulting in a 10-year risk of developing CHD of more than 20 percent [2, 4]. LDL-cholesterol (LDL-C) levels are indications for the initiation of treatment and represent therapeutic targets, but these targets are achieved by only one-third of all patients, and even fewer of those with established CHD. LDL-C levels are the primary target of treatment, with LDL-C and triglyceride levels forming secondary goals in these guidelines [5]. For individuals with elevated triglycerides, the primary goal remains achieving the appropriate LDL-C target. The ATP III recommendations do not specify a target for LDL-C increment due to insufficient evidence regarding the proper level.

Medications available for lipid-lowering therapy have various mechanisms of action and pharmacokinetic properties. The most widely prescribed are the 3-hydroxy-3-methylglutaryl coenzyme A (HMG-CoA) reductase inhibitors, known as statins [6]. These agents reduce the production of cholesterol in the liver by binding with the enzyme responsible for its production. In contrast, fibrates do not influence lipid synthesis but rather reduce the levels of fatty acids in the blood. Ezetimibe is an agent that inhibits intestinal absorption by acting on the sterol transporter NPC1L1 [7]. Niacin (nicotinic acid) reduces LDL-C and increases HDL-cholesterol (HDL-C) via a mechanism yet to be fully elucidated, although it is suspected to be involved in the synthesis and metabolism of apolipoproteins. Bile acid sequestrants (BAS) bind bile

acids in the bowel, thereby preventing reabsorption of bile from the intestine. Omega-3 fatty acids have been postulated to lower postprandial triglycerides and have antithrombotic and blood-pressure-reducing effects [5].

2.2 Investigational Product

After diet and life-style changes, statins (HMG-CoA reductase inhibitors), introduced in the late 1980s, are widely used as first-line agents for lipid lowering in individuals with or are 'at risk' of cardiovascular disease including those with familial hypercholesterolaemia. Statins inhibit the rate-limiting enzyme in cholesterol production and, hence, up-regulate hepatic LDL receptors increasing the removal of apolipoprotein B-containing lipoproteins from plasma [8]. HMG-CoA reductase inhibitors are often administered for the treatment of hypercholesterolemia in patients with type 2 diabetes. Subgroup analysis of the 4S, CARE, LIPID, and HPS studies has indicated that administration of pravastatin or simvastatin decreases the development of coronary artery disease, the mortality from heart disease, and the overall mortality in patients with type 2 diabetes mellitus [9]. More recently, revisions to the guidelines have established a new and lower target for LDL cholesterol than was previously considered necessary, with the aim of reducing the cardiovascular disease risk.

Atorvastatin, a lipophilic HMG-CoA reductase inhibitor, has potent LDL-C-lowering activity and is frequently administered for the treatment of hypercholesterolemia associated with type 2 diabetes mellitus [3, 9]. In the recent CARDS study, atorvastatin was shown to inhibit the development of cardiovascular events in DM patients. However, ASPEN study did not find any significant beneficial effect of atorvastatin in such patients. Thus, disagreement persists as to which statins should be used for the treatment of dyslipidemia in diabetics. It has been a matter of recent concern whether or not atorvastatin causes a deterioration of glycemic control. Sasaki *et al*. have comprehensively reviewed the adverse effect of statins on glucose metabolism. There are only a few relevant clinical trials. High-dose atorvastatin therapy was associated with worsening of glycemic control in one trial. In Japan, there have been a few case reports and some clinical trials that have indicated a potential adverse effect of atorvastatin, although these studies were small in size and had a short follow-up period. In safety issue, most common treatment-associated adverse event was gastrointestinal symptom, mainly to a greater incidence in diarrhea. Serious adverse events were rare and seldom led to withdrawal. Persistent elevations in hepatic transaminases to > 3 times the upper limit of normal (ULN) were experienced by 0.5% of atorvastatin-treated patients [10]. A persistent elevation in creatine phosphokinase (>10 x ULN) was observed in even less percentage of population. The incidence of treatment-associated myalgia was low (1.9%) in atorvastatin treatment population in general. Rhabdomyolysis incidence in Graham *et al.* study [11] showed 0.44% for monotherapy with atorvastatin. In overall incidence of treatment-associated adverse events observed with atorvastatin did not increase in the 10- to 80-mg dose range, and was similar to that observed with placebo and in patients treated with other statins. Musculoskeletal and hepatic adverse events showed that these occurred infrequently and rarely resulted in treatment discontinuation [7, 10].

Pitavastatin is an HMG-CoA reductae inhibitor that is commonly used in Japan and has a powerful lipid-lowering effect [12]. It was discovered in Japan by Nissan Chemical

Industries, Ltd, and jointly developed further with Kowa Company, Ltd, Tokyo, and has been available in Japan since 2003. Pitavastatin was recently approved for use in the United States by the FDA on March 8th 2009 under the trade name Livalo. Pitavastatin has been also approved by the Medicines and Healthcare products Regulatory Agency (MHRA) in UK on August 17th 2010. It is known to metabolize minimally by human cytochrome P450 (CYP), which may lead to the possibility of less drug interactions. The reduction of LDL-C by pitavastatin is comparable to that achieved with atorvastatin, while pitavastatin also raises the level of HDL-C [13, 14]. From previous clinical studies, such as NK-104-301, NK-104-305, pitavastatin was compared with atorvastatin in primary hyperlipidemia or dyslipidemia/ diabetes mellitus patients, pitavastatin had shown non-inferior to atorvastatin in lowering LDL-C, total cholesterol (TC), TG and apolipoprotein B (Apo B) as well increasing HDL-C plasma level in 12-week study trial [15, 16]. The safety and tolerability on the other hand is well tolerated in hypercholesterolemic patients according to the post-marketing LIVES study [17]. Most common adverse events were increased in blood CK, AST, ALT and GGT. No clinical significant abnormalities were associated with pitavastatin in laboratory abnormalities. From LIVES study, myalgia was uncommon (1.08 %) and 0.005 % incidence developed rhabdomyolysis with creatine phosphokinase at least ten-times the upper limit of normal. In addition, pitavastatin was relatively safe to use in elderly and no need to adjust the dosage [17].

2.3 Study Rationale

Pitavastatin (Livalo®) is a new highly effective statin observed in nowadays. The ideal statin can introduce maximum up-regulation in LDL receptors, has a prolonged period of action, has low potential for drug interaction, and is minimally exposed to peripheral tissue. Further comparative studies of pitavastatin and other HMG-CoA reductase in different patient/race group is necessary. For such reasons, we plan to conduct this phase IIIb trial of pitavastatin to determine its non-inferiority to atorvastatin and to evaluate the efficacy and safety in high risk hypercholesterolemic patients in Taiwanese population. The investigational product will be orally administered 2 mg QD for a study period of 12 weeks. This trial is prepared for future pre-license marketing applications in Taiwan.

3. Study Objectives and Endpoints

3.1 Study Objectives

3.1.1 Primary Study Objective

The primary study objective is to compare pitavastatin 2 mg versus atorvastatin 10 mg given once daily in the LDL-C lowering effect in patients with hypercholesterolemia.

3.1.2 Secondary Study Objectives

The secondary objectives of this study are as follows:

1. To compare the other lipid and lipoprotein efficacy of pitavastatin 2 mg versus atorvastatin 10 mg given once daily in LDL-C, HDL-C, TG, non-HDL-C, Apo A1, and Apo B lowering effects in patients with hypercholesterolemia.

2. To compare the non-lipid efficacy of pitavastatin 2 mg versus atorvastatin 10 mg given once daily in fasting plasma glucose, fasting insulin level, HbA1c, free fatty acid, ADMA, and HOMA-IR lowering effects in patients with hypercholesterolemia.

3. To compare the safety and tolerability of pitavastatin 2 mg versus atorvastatin 10 mg given once daily by clinical and laboratorial results and adverse events.

   Note: a marked deterioration of glycemic control has been reported in some patients treated with atorvastatin. Therefore, this stratification of DM and non-DM groups is use to compare the effects of pitavastatin with atrorvastatin on glycemic control. All efficacy endpoints, including primary endpoint, will be statistically analyzed on DM and non-DM groups, respectively, as well as pooled data.

3.2 Endpoints

3.2.1 Primary Endpoints

The mean percentage change of the week-12 (Visit 5) evaluation compared with the baseline on the LDL-C blood levels of the patients between pitavastatin and atorvastatin

3.2.2 Secondary Endpoints

Efficacy

Lipid efficacy

1. To estimate the proportion of patients achieving LDL-C blood level to < 100 mg/dL at the week-12 (Visit 5).
2. The mean percentage change of LDL-C blood level comparing baseline to week-4 (Visit 3).

3. The mean percentage changes of HDL-C blood level comparing baseline to week-4 (Visit 3) and week-12 (Visit 5).
4. The mean percentage changes of TG blood level comparing baseline to week-4 (Visit 3) and week-12 (Visit 5).
5. The mean percentage changes of non-HDL-C blood level comparing baseline to week-4 (Visit 3) and week-12 (Visit 5) (non-HDL-C = TC – HDL-C).
6. To evaluate the net changes of Apo A1 and Apo B comparing baseline to the week-12 (Visit 5).

Non-lipid efficacy

1. To evaluate the net changes of fasting plasma glucose from the baseline to the week-4 (Visit 3) and week-12 (Visit 5).
2. To evaluate the net changes of fasting insulin level from the baseline to the week-12 (Visit 5).
3. To evaluate the baseline and week-12 (Visit 5) insulin resistance by the homeostasis model assessment method (HOMA-IR), in equation of:

$$\text{HOMA-IR} = \frac{\text{fasting insulin level } (\mu\text{U/ml}) \times \text{fasting plasma glucose (mg/dL)}}{405 \text{ (mmol/L)}}$$

4. To evaluate the net change of HbA1c comparing the baseline to the week-12 (Visit 5).
5. To evaluate the net change of free fatty acid comparing the baseline to the week-12 (Visit 5).
6. To evaluate the net change of asymmetric dimethylarginine (ADMA) from the baseline to the week-12 (Visit 5).

Note: all efficacy endpoints, including primary endpoint, will be statistically analyzed on DM and non-DM groups, respectively, as well as pooled data.

Safety

Clinical safety

5. To evaluate the changes in physical examination results.
6. To evaluate the changes in vital signs (pulse, systolic and diastolic blood pressures).
7. To evaluate the changes in urinalysis, including pH, erythrocyte, leukocyte, glucose, and protein (qualitatively).
8. To evaluate the change of EKG results (including PR, QRS, QT, QTc, RR intervals).

Laboratory safety

To evaluate the net change of safety profile from baseline to each visit according to the flow chart (See **1.2.1 Clinical Flow Chart**) by the follow laboratory tests:

- White blood cell (WBC) with differential counts
- Red blood cell (RBC)
- Hemoglobin (Hb)
- Hematocrit (Hct)
- Platelet (PLT)
- C-reactive protein (CRP)
- Creatinine kinase (CK)
- Aspartate aminotransferase (AST)
- Alanine aminotransferase (ALT)
- γ-Glutamyl transpeptidase (GGT)
- Total bilirubin
- BUN
- Serum creatinine
- Lactate dehydrogenase (LDH)

Adverse event (AE)

To evaluate AEs incidences

4. Study Design

4.1 Type and Design

This is a randomized, double-blind, active-controlled, non-inferiority study carrying in a multi-center setting. The study is designed for evaluating lipid lowering effects of Pitavastatin as a new HMG-CoA reductase inhibitor drug.

The study will be a 12-week treatment period study. Study medication dose will be given in Pitavastatin (Livalo®) 2 mg QD and Atorvastatin (Lipitor®) 10 mg QD.

At Visit 1, patients will require completing designed assessments and examinations, including physical examinations, demographics, medical history, medication history, vital signs, EKG, and clinical laboratory tests to evaluate and recruit the possible candidates. The Visit 1 may involve up to 14 days before Visit 2. Patients are base on Visit 1 examinations to justify their eligibility to enroll in the study. All the laboratory test results obtained from Visit 1 will be used for baseline calculation. On Visit 2, recruited patients will be asked to complete required assessments. No additional performance for laboratory tests is needed in Visit 2. However, when investigator has any concern about the patient's condition, an extra performance to withdraw blood for required laboratory tests is allowed. Additional examinations obtained from Visit 2 will be used as safety purposes. This is an optional re-check point for investigators to assure patient's eligibility and safety for this study. Furthermore, if the patient has undergone an additional laboratory test on Visit 2, the test results obtained from the Visit 2 will be used as the baseline calculation, combining with other laboratory tests obtained from Visit 1. Study medications will be dispensed to the randomized patients at Visit 2, Visit 3 and Visit 4 by a 4-weekly basis. If patients have an additional laboratory tests at Visit 2, study medications will be dispensed after rechecking the laboratory test result is acceptable for eligibility (approximate 1 week after Visit 2). Visit 3 and Visit 4 will be carried on week 4 (28±5 days) and week 8 (56±5 days). The laboratory tests for safety and efficacy evaluation will be performed on Visit 3 (week 4, 28±5 days). There is no necessary to carry another laboratory tests on Visit 4 (week 8, 56±5 days), unless an additional concern has been made by the investigator. The Visit 5 will be on week 12 (84±5 days). The concomitant medications, dispense study drug, laboratory exams, the collection of unused treatment medications, and adverse event will be performed/recorded according to clinical flow chart (See **1.2.1 Clinical Flow Chart** for detailed information).

There will be no change in study drug doses across the entire study duration. The study drug is dispensed according to the treatment schedule in the study protocol, by a 4-weekly basis. Patients are dosed once daily in the evening after dinner.

4.1.1 Sample Size

Patients will be recruited for at least a total of 144 evaluable cases. The total randomized patient number in this study is estimate of about 226 cases.

4.1.2 Choice of Control

Atorvastatin was chosen as the comparator because of its most commonly used and well-studied statin in clinical use among Europe and USA. Atorvastatin doses of 10 to 80 mg once daily was commonly used and given plasma lipid reduction in satisfy response. The initial EMEA and FDA approval for the use of atorvastatin was granted to treat hypercholesterolemia and mixed dyslipidemia. In additional, we would like to demonstrate the efficacy and safety of 2 mg of pitavastatin versus 10 mg of atorvastatin.

4.2 Avoid/Minimize Bias

This is a two-arm study, randomized, double-blind evaluation at 6 hospital centers to examine the effect of 12-week treatment with pitavastatin versus atorvastatin in hypercholesterolemic patients. A total of 226 randomized patients were planned, with ratio of 1:1, giving 100 patients in either pitavastatin 2 mg QD or atorvastatin 10 mg QD treatment groups.

Both study medications are manufactured in tablet. Both of them will be encapsulated (with the same color, texture, odor and size of hard gelatin capsule) for masking to minimize any possible bias from appearance judgments. The study procedures are well-specified such that any anticipated irregularities in trial conduct that might impair a satisfactory analysis, including protocol violations, withdrawals and missing values can be minimized. The protocol also considers ways to reduce the frequency of irregularities, and also to handle the problems that might occur in data analysis.

4.3 Dose Rationale

The majority clinical efficacy of pitavastatin in human study is based in Japan. Phase II and phase III trials had also been studied in the US and Europe. Studies on patients with primary hypercholesterolaemia and combined hyperlipidemia at doses of 1, 2, 4, and 8 mg of pitavastatin were well tolerated, with satisfying reduction in TC, LDL-C, TG and increase of HDL-C [11]. Two mg pitavastatin was shown to safely lower lipid profile and was well tolerated at this dose, and a favorable risk benefit ratio was expected in this study. The dose rationale chose in 2 mg pitavastatin and 10 mg atorvastatin design was based on previous clinical trials (NK-104 study), most of the comparison dose was set at oral dose of 2 to 4 mg pitavastatin once daily for effective treatment result.

4.4 Rationale of Food Intake Related to IP Administration

In general, the ingestion of food, concurrent with study drug administration, has a variable effect on the degree of absorption of statins. Plasma levels of atorvastatin are decreased when the drug is taken with food. However, food does not affect the plasma LDL-C lowering efficacy of atorvastatin. Neither the absorption nor the bioavailability of pitavastatin is affected by food. In this study trial, both pitavastatin and atorvastatin are set to be administered in the evening after dinner for patients. Some natural health foods have been discovered of interfere/modifying with cholesterol and lipid profile. For example, red yeast rice, eicosapentaenoic acid (EPA), docosahexaenoic acid (DHA), allicin, oatmeal and lycopene. These natural health foods listed in **Appendix 13.4** are recommended to avoid for at least 4 weeks as well

under recommended daily intake (RDI).

5. Study Population

5.1 Number of Patients

A total of 226 randomized patients were planned, with ratio of 1:1, giving 100 patients in either pitavastatin 2 mg QD or atorvastatin 10 mg QD treatment groups. The evaluable cases are estimated in around of 144 patients.

5.2 Eligibility Criteria

5.2.1 Inclusion Criteria

To be eligible for inclusion, each patient must fulfill all of the following criteria:

1. Patient aged ≥ 20 years old and < 75 years old.
2. Patient who was eligible and able to participate in the study and accepts to enter the study by signing written informed consent.
3. Patient with fasting LDL-C > 100 mg/dL. The concentration of LDL-C is obtained from laboratory examination.
4. Patient with at least one of the following description (NCEP ATP III guideline):
   - ➢ Documented coronary heart disease (CHD)
   - ➢ Type 2 diabetes mellitus (DM)
   - ➢ If below 2+ risk factors (other than LDL) are present without CHD or CHD risk equivalent, assess 10-year (short-term) CHD risk (see Framingham score) > 20%.
     - ✓ Female age ≥ 55 years old and male age ≥ 45 years old.
     - ✓ Fasting high-density lipoprotein cholesterol (HDL-C) < 40mg/dL.
     - ✓ Patient who has family history of premature CHD (CHD in male first degree relative < 55 years; CHD in female first degree relative < 65 years).
     - ✓ Hypertension (BP ≥ 140/90 mmHg or treated with anti-hypertensive agents).
     - ✓ HDL-C ≥ 60 mg/dL count as “negative” risk factor; its presence removes one risk factor from the total count.
5. Female patient with child-bearing potential must take reliable contraception method(s) during the participation of the study.

5.2.2 Exclusion Criteria

1. Patient who has participated in other investigational studies within 3 months before Visit 2.
2. Patient took medication and natural health foods (see **Appendix 13.3** and **13.4**) known to alter blood lipid profiles within 4 weeks of Visit 2.
3. Patient is taking any medication or food (see **Section 8.2**) that is prohibited

by the study.

4. Patient taking Amiodarone will be excluded from this study (due to long half life of this medication).
5. Patient is diagnosed with type 1 DM or has been using insulin/insulin analog medication.
6. Patient with a history of multiple drug allergies or with a known allergy to hydroxy-methylglutaryl coenzyme-A (HMG-CoA) reductase inhibitors.
7. Patient with triglyceride (TG) > 400 mg/dL.
8. Excessive obesity defined as body mass index (BMI) above 35 kg/m^2.
9. Cerebral vascular disease (including cerebrovascular hemorrhage or ischemia, transient ischemic attack) diagnosed within 3 months before Visit 2.
10. Cerebral vascular disease (including cerebrovascular hemorrhage or ischemia, transient ischemic attack) diagnosed within 3 months before Visit 2.
11. Patient with advanced renal disorder (Serum creatinine levels ≥ 2 mg/dL and blood urea nitrogen (BUN) ≥ 25 mg/dL) at Visit 1.
12. Patient with advanced hepatic disorder (aspartate aminotransferase (AST) or alanine aminotransferase (ALT) level ≥ 100 IU/L).
13. Patient with creatine kinase (CK) level > 5 × ULRR at any time point between Visit 1 and Visit 2.
14. Patient with poorly controlled diabetes mellitus (HbA1c > 9.0%) or patient with severe hypertension (> 180 mmHg for systolic or > 120 mmHg in diastolic blood pressure).
15. Patient with hypothyroidism, hereditary muscular disorders, family history of the above or history of drug-induced myopathy.
16. Patient has significant alcohol consumption (> 65 mL pure alcohol) within 48 hours before Visit 2.
17. Any major surgery within 3 months prior to Visit 2.
18. Female patient who is lactating, being pregnant or plans to become pregnant.
19. Patient with conditions judged by the investigator as unsuitable for the study.

5.2.3 Withdrawal Criteria

The patients should be withdrawn from the study if any of the conditions set below has occurred:

1. Patient decides to withdraw his/her informed consent form.

2. Patient lost follow-up.
3. Investigator considers that the patient is no longer physically and/or psychologically feasible to remain in the study.
4. Patient has developed the condition that prohibited drug(s)/treatment(s) become inevitable to be taken by the patient.
5. Patient has developed any suspected clinical conditions (e.g., muscle pain, muscle soreness, and fatigue, etc) judged by investigator as well as CK level > 10 × ULRR or AST or ALT > 3 × ULRR at any time point during study.
6. Patient develops adverse effects that the investigator considers a permanent cessation of the study treatment is necessary.

Patients who, after Visit 2, discontinued prematurely from the study were not replaced. All patients who discontinued early were encouraged to complete all efficacy and safety evaluations corresponding to week 12 (Visit 5) as soon as possible after discontinuation from study treatment.

The study completion CRF page must have been completed for all patients who entered the active treatment period of the study, even if the patient refused to return for a final visit (Visit 5).

6. Study Assessment and Procedures

6.1 Study Assessment

6.1.1 Informed Consent

The investigator must explain to each patient the nature of the study, its purpose, the procedures involved, the expected duration, the potential risks and benefits involved and any predicted discomforts. Each patient was informed that participation in the study was voluntary and that he/she can withdraw their participation at any time.

All patients must provide signed and dated informed consent at screen visit prior to any study-related procedures. Only the institutional review board (IRB) and the applicable health authorities approved informed consent form can be used.

6.1.2 Eligibility

Eligibility should be thoroughly checked by the investigators at Visit 1 and Visit 2. However, when the investigator has any concern about the patient's condition, an extra performance to withdraw blood for required laboratory tests is allowed. Additional examinations obtained from Visit 2 will be used as safety purposes. This is an optional re-check point for investigators to assure patient's eligibility and safety for this study. See **Section 5.2 Eligibility Criteria** for detailed eligibility criteria.

6.1.3 Height

Height is to be measured for the patient not wearing shoes at Visit 1. The measure of height will be rounded to the nearest centimeter.

6.1.4 Demographics

Demographic data including age, date of birth, gender, BMI, primary diagnosis and duration of current disease information will be obtained at Visit 1.

6.1.5 Medical History

The general medical history up to 3 months of study entry (Visit 1) should be recorded at Visit 1. The medical history should include procedural and surgical history within 1 year time.

6.1.6 Concurrent disease/status

Full details of concurrent disease/status should be recorded at each visit.

6.1.7 Urine Pregnancy Test

Patient with childbearing potential must be confirmed of not being pregnant at each visit and being well-informed that effective contraceptive method must be used from Visit 1 to Visit 5 and also during 6 months after exiting from the study.

6.1.8 Vital Signs and body weight

Patient will be measured of vital signs including blood pressures, pulse rate, respiratory rate and body temperature at all visits. Weight will be measured at each visit.

6.1.9 Framingham projection

Patients will be estimated his/her cardiovascular risk at Visit 1 by assessing Framingham risk score, including age, sex, TC, HDL-C, smoking status and blood pressure (See **Appendix 13.2**). The 10-year risk of a cardiovascular event can be calculated with approximately 75% accuracy.

6.1.10 Physical Examinations

Patient will be examined at Visit 1, Visit 2 and Visit 5 by standard physical examination items including general appearance, skin, eyes, ear/nose/throat (ENT), head and neck, heart, chest and lungs, abdomen, extremities, lymph nodes, musculoskeletal, neurological, and other body systems if applicable for describing the status of the patient’s health.

6.1.11 Laboratory Tests

The blood laboratory tests are collected at individual study site and analyzed in the qualified central laboratory, Union Clinical Laboratory (UCL) in Taipei, Taiwan. Examination tests including RBC, WBC with differential counts, platelet count, hemoglobin, hematocrit, LDL-C, TG, HDL-C, Apo A1, Apo B, fasting glucose, fasting insulin level, HbA1c, fatty acid, CRP, CK, AST, ALT, GGT, total bilirubin, BUN, serum creatinine, LDH and ADMA will be taken by scheduled visits (Refer to Clinical flow chart on **Section 1.2.1**). The urinalysis including pH, erythrocyte, leukocyte, glucose and protein will be conducted at Visit 1 and Visit 5.

Asymmetric dimethylarginine (ADMA) is involved in the pathogenesis of hypertension and atherosclerosis through its inhibition of the formation of the endogenous vasculoprotective molecule, nitric oxide (NO). NO has also been described as an endogenous anti-atherosclerotic molecule. Determination of ADMA can help to predict both the likelihood of developing cardiovascular disease and its prognosis. It has been suggest that even small changes in ADMA concentration can significantly alter vascular NO production, vascular tone and systemic vascular resistance. The ADMA marker will be evaluated by investigators for exploratory purposes. The evaluation will be made at Visit 1 and Visit 5.

No additional performance for laboratory tests is needed in Visit 2. However, when investigator has any concern about the patient’s condition, an extra performance to withdraw blood for required laboratory tests is allowed. After the test results are revealed, the investigators should judge at their discretion whether patients are still eligible to stay at the study, especially for those patients whose test results slightly deviate from entry criteria. Such allowance to patients to continue study should not be considered as wrong entry. Hence then the laboratory tests perform in Visit 2 is an

optional re-check point for the investigators to assure patient's eligibility and safety for this study. Furthermore, if the patient has undergone an additional laboratory test on Visit 2, the test results obtained from Visit 2 will be used as the baseline calculation, combining with other laboratory tests obtained from Visit 1.

In Visit 3 and Visit 4, the laboratory tests for safety and efficacy evaluation will be performed on week 4 (28±5 days). There is no necessary to carry another laboratory evaluations on week 8 (56±5 days), unless an additional concern has been made by investigator.

6.1.12 HOMA-IR

Subjeccts will record fasting insulin and glucose level to obtain Homeostasis model assessment method (HOMA-IR) at Visit 1 and Visit 5, in equation of:

$$\text{HOMA-IR} = \frac{\text{fasting insulin level } (\mu\text{U/ml}) \text{ x fasting plasma glucose (mg/dL)}}{405 \text{ (mmol/L)}}$$

This is a method used to quantify insulin resistance and beta-cell function.

6.1.13 Primary efficacy endpoint

The mean percentage change of LDL-C blood level from baseline to endpoint (Visit 5, week-12) are evaluate of pitavastatin and atorvastatin treatment groups. Blood samples must have been obtained fasting (at least 12 hours without food). If the patient had not fasted, the laboratory sampling should have been re-scheduled within the following 2- to 3-days and explanatory comment must been provided.

Investigators, their staff, patients and the sponsor (except some designated people involved in data validation and programming) will keep in blind to the lipid level results obtained at visits until the study is complete and the database locked and unblended. Only the results from the UCL are consider valid for qualification.

6.1.14 Secondary efficacy endpoint

The secondary efficacy endpoints including the proportion of patients achieving LDL-C blood level, the percentage change from baseline in LDL-C, HDL-C, TG, non-HDL-C are collect at all scheduled visits. Apo A1 and Apo B are obtaining at Visit 1 and Visit 5.

Baseline for LDL-C, HDL-C, TG and non-HDL-C are calculated as the mean of the lipid measurements from Visit 1 (week ≤ -2) or Visit 2 (if the patient has re-checking test results from Visit 2). Baseline for Apo A1 and Apo B are base on the result at Visit 1 (week ≤ -2) as well.

The NCEP Adult Treatment Panel III Guidelines are used to scoring each patient based on their cardiovascular risk factors. Details are outlined in the **Appendix 13.2**.

Non-lipid efficacy endpoints, including the net change of fasting plasma glucose from baseline to week-4 (Visit 3) and week-12 (Visit 5), fasting insulin level, HbA1c, free fatty acid and ADMA are evaluate the net change between baseline and the week-12

(Visit 5). HOMA-IR evaluation is comparing the baseline and Visit 5 (week-12).

6.1.15 Patient Identifier

Patient enrolled at Visit 1 will be assigned an identification number, namely, patient identifier. The patient identifier contains the first 1 digit of “center number”, the middle 3 digits of screening number and the last 3 digits of randomization number which assigned when the eligibility is confirmed at both visit 1 and visit 2 before the administration of study treatment. The screening number and randomization number of eligible subjects are centralized assigned sequentially from 001 via an Interactive Voice Response (IVR) system (Example: for subject of study site 1, the second screened subject and the first eligible subject of all study sites will be assigned patient identifier as 1-002-001). Patient screened but not eligible will be identified by “X” and “sequential number (non-centralized, starts from X01 for each site)” for the last 3 digits of the patient identifier (Example: first screened but not eligible: 1-001-X01).

After screening, if patient is eligible to participate in this trial, the investigator will receive a randomization number and a corresponding package number via IVRS at Visit 2. The clinical trial pharmacist/investigator designated personnel prepares and administers the blindly packaged investigational product, which is indistinguishable in all aspects.

6.1.16 Previous and Concomitant Medications

Previous medications should be recorded up to 6 weeks before study entry at Visit 1.

All concomitant medications starting from enrollment of patients should be recorded.

6.1.17 Investigational Drug

Eligible patients will be dispensed IP at Visit 2, Visit 3 and Visit 4.

At Visit 2, IP will be dispensed in encapsulated form to maintain the blind in this study. The dosage of pitavastatin (Livalo®) is 2 mg versus atorvastatin (Lipitor®) 10 mg. After clinical and criteria evaluations, investigator will give the IP to patients for home to use.

During Visit 3 (week 4) and Visit 4 (week 8), patients will be asked to return all unused IP prescribed from previous visit until Visit 5 (week 12).

Drug accountability and inventory will be recorded in the CRFs and the relevant study log.

6.1.18 Adverse Event (AE)

Patients will be asked to report AE voluntarily and the investigators will also examine the patients to identify AE for all visits since Visit 2. See **Section 9 Adverse Events (AE) and Serious Adverse Events (SAE)** for AE in more details.

6.1.19 EKG

EKG examinations will be performed at Visit 1 and Visit 5. The evaluation items will include PR, QRS, QT, QTc, and RR intervals. EKG test results within 30 days before Visit 1 are acceptable.

6.1.20 Exit of Study

The patient will be dismissed from the study at Visit 5.

6.2 Randomization & Blinding Process Management

The randomization method will be "Permuted block randomization" stratified by DM status, centralized randomization will be applied. Upon the monitor's request to generate a randomization list, a statistician will review the parameters and write up a Statistical Analysis System (SAS) program. The block size is determined by the QA manager as appropriate to the study design. The randomization list is generated by the QA associate and reviewed by the QA department head using the validated program prepared by statistician with randomization seed revised by the QA associate. The statistician can not have access to the randomization list.

The randomization list (or individual treatment code) for the blind study should be made into five copies and placed in 5 sealed envelopes. One for VCRO system administrator, one for QA associate, third one is for drug packaging purpose and will be destroyed afterwards. The fourth one is to be made into individually sealed envelopes and placed in a large envelope for each site use. The last one is to be made into individually sealed envelopes and placed in a large envelope for Kowa Company Ltd., the marketing company for pitavastatin in Japan.

After screening, if a patient is finally judged to be eligible to participate in this trial (Visit 2, week 0), the investigator will receive a randomization number and a corresponding package number via an Interactive Voice Response (IVR) system. The clinical trial pharmacist/investigator designated personnel prepares and administers the blindly packaged investigational product, which is indistinguishable in all aspects.

In case of any emergency and the information on treatment assignment is needed in order to protect the patient, the investigator can open the double blind code saved in individually sealed envelope for the relevant patient only. Investigator should promptly notify VCRO, preferably before de-code, and record the date and time of unblinding, as well as detailed reasons for unblinding on the patinet's chart, CRF, and the opened envelope.

6.2.1 Visit 1

(Within 14 days before Visit 2)

- Explain the nature of the study and have patients to read and sign an Informed Consent Form
- Screen patients for inclusion/exclusion criteria
- Obtain height

- Obtain demographic characteristics
  - Age
  - Date of birth
  - Gender
  - BMI
  - Primary diagnosis
  - Duration of current disease information
- Obtain general medical history (including smoking history)
- Perform urine pregnancy test for applicable patients only
- Obtain vital signs and body weight
- Obtain concurrent diseases/ status
- Cardiovascular risk factor assessment (Framingham risk score)
- Perform physical examinations
  - General appearance
  - Skin
  - Eyes
  - Ears, nose, throat
  - Head and neck
  - Chest and lungs
  - Abdomen
  - Extremities
  - Lymph nodes
  - Musculoskeletal
  - Neurological
  - Other system
- Perform EKG examinations
- Perform blood lab tests:

  Hematology
  - White blood cells with differential counts
  - Red blood cells
  - Hemoglobin
  - Hematocrit
  - Platelet

Safety evaluation

- CRP
- CK
- AST
- ALT
- GGT

Safety evaluation

- Total bilirubin
- BUN
- Serum creatinine
- LDH

Fasting plasma lipids

- Total cholesterol
- LDL-C
- TG
- HDL-C

➢ Urinalysis

- pH
- RBC
- WBC
- Glucose
- Protein

➢ Fasting insulin level

➢ Fasting plasma glucose

➢ HOMA-IR calculation

➢ HbA1c

➢ Apo A1 and Apo B

➢ Fatty acid

➢ ADMA

➢ Record concomitant medications

6.2.2 **Visit** 2

(Week 0, Day 0)

- Screen patients for inclusion/exclusion criteria (without laboratory tests).
- Perform urine pregnancy test for applicable patients only
- Obtain vital signs and body weight
- Obtain concurrent diseases/ status
- Perform physical examinations
- Assign patient randomization number to eligible patients by IVRS
- Perform laboratory (biochemistry) tests (optional: if the investigator considers it necessary)
  - CRP
  - CK
  - AST
  - ALT
  - GGT
- **Urinalysis (optional: if the investigator considers it necessary)**
- Perform fasting plasma glucose (optional: if the investigator considers it necessary)
- Perform fasting plasma lipid (TC, TG, LDL-C, HDL-C) profile (optional: if the investigator considers it necessary)
- Dispense investigational product (If patients have an additional laboratory tests at Visit 2, study medications will be dispensed after rechecking the laboratory test result is acceptable).
- Record concomitant medications
- Record AE

  Note: Laboratory tests performed in this visit are an optional re-check point for investigators to assure patient's eligibility and safety for this trial.

6.2.3 **Visit** 3

(Week 4, 28 ± 5 days)

- Perform urine pregnancy test for applicable patients only
- Obtain vital signs and body weight
- Obtain concurrent diseases/ status

- Perform laboratory (biochemistry) tests
  - CRP
  - CK
  - AST
  - ALT
  - GGT
- Perform fasting plasma glucose
- Perform fasting plasma lipid (TC, TG, LDL-C, and HDL-C) profile
- Dispense investigational product
- Retrieve unused investigational product
- Record concomitant medications
- Record AE

6.2.4 Visit 4

(Week 8, 56 ± 5 days)

- Perform urine pregnancy test for applicable patients only
- Obtain vital signs and body weight
- Obtain concurrent diseases/ status
- Perform laboratory (biochemistry) tests (optional: if the investigator considers it necessary)
  - CRP
  - CK
  - AST
  - ALT
  - GGT
- Perform fasting plasma glucose (optional: if the investigator considers it necessary)
- Perform fasting plasma lipid (TC, TG, LDL-C, and HDL-C) profile (optional: if the investigator considers it necessary)
- Dispense investigational product
- Retrieve unused investigational product
- Record concomitant medications
- Record AE

6.2.5 Visit 5

(Week 12, 84 ± 5 days)

- Perform urine pregnancy test for applicable patients only
- Obtain vital signs and body weight
- Obtain concurrent diseases/ status
- Perform physical examinations
- Perform EKG examinations
- Perform blood lab tests:

  Hematology
  - White blood cells with differential counts
  - Red blood cells
  - Hemoglobin
  - Hematocrit
  - Platelet

  Safety evaluation
  - CRP
  - CK
  - AST
  - ALT
  - GGT

  Safety evaluation
  - Total bilirubin
  - BUN
  - Serum creatinine
  - LDH

  Fasting plasma lipids
  - Total cholesterol
  - LDL-C
  - TG
  - HDL-C
- Perform Urinalysis

- pH
- RBC
- WBC
- Glucose
- Protein

- ➢ Fasting insulin level
- ➢ Fasting plasma glucose
- ➢ HOMA-IR calculation
- ➢ HbA1c
- ➢ Apo A1 and Apo B
- ➢ Fatty acid
- ➢ ADMA
- ➢ Retrieve unused investigational product
- ➢ Record concomitant medications
- ➢ Record AE
- ➢ Complete exit form
- ➢ Dismiss patient

7. Investigational Product

7.1 Description of Investigational Product and the Comparator Drug

Pitavastatin 2 mg for this study will be supplied by Tai Tien Pharmaceuticals Co., Ltd. Livalo® is a registered trademark of the Kowa Company, Ltd.

1. Generic Name: Pitavastatin
2. Chemical Structure: $C_{50}H_{46}CaF_2N_2O_8$
3. Molecule weight: 880.98 kDa
4. Method of storage: Room temperature between 15℃ and 30℃ (59°F to 86°F). Protect from light.
5. Directions: Slightly light-colored yellow-red scored film-coated tablet ground and encapsulated for this study. Pitavastatin is recommended to take after dinner.

The comparator, atorvastatin 10 mg will be purchased and supplied by Tai Tien Pharmaceuticals Co., Ltd. It is manufactured by Pfizer Inc.

1. Generic Name: Atorvastatin
2. Chemical Structure: $(C_{33}H_{34}FN_2O_5)_2Ca{\cdot}3H_2O$
3. Molecule weight: 1209.42 kDa
4. Method of storage: Room temperature between 20℃ and 25℃ (68°F to 77°F). Protect from light.
5. Directions: Elliptical white film-coated tablet ground and encapsulated for this study. Atorvastatin can be taken at any time of the day with or without food.

The physicochemical properties and the pharmaceutical specifications of both IP for this study are provided in the Investigator's Brochure (IB). The capsule is made of hard gelatin in the length of 20 mm (size 0 capsule). Both pitavastatin and atorvastatin tablets are encapsulated individually in the same type capsule with identical appearance. The excipient was made of lactose.

The sponsor is responsible to ship the pitavastatin and atorvastatin drug to study site and strict inventory control for the drug accountability will be implemented.

7.2 Dosage and Administration

The dosage of pitavastatin and atorvastatin used in this study are 2 mg and 10 mg, respectively. Each patient will be instructed to take 1 encapsulated pitavastatin or comparator drug, encapsulated atorvastatin, orally with about 250 ml water in the evening after dinner in each day. No dose modification plan is anticipated for this study. Any unexpected adverse event should be handled according to the **Section 5.2.3** and **Section 9**.

7.3 Dose Rationale

The study aim is to investigate the efficacy and safety profiles of pitavastatin versus atorvastatin in patients with high risk of hypercholesterolemia. Based on literatures and the clinical study results listed in the IP prescribing information, pitavastatin appeared to be of good effectiveness and is safe in decrease lipid profile for patients with hypercholesterolemia.

The dose range for pitavastatin is 1 to 4 mg orally once daily. The recommended starting dose is 2 mg and the maximum dose is 4mg. Doses of pitavastatin greater than 4 mg once daily were associated with an increased risk for severe myopathy in premarketing clinical studies.

The comparator drug, atorvastatin, has dose range from 10 to 80 mg orally once daily. The recommended starting dose is 10mg once daily.

The dose rationale chose in 2 mg pitavastatin and 10 mg atorvastatin design was based on previous clinical trials (NK-104 study), most of the comparison dose was set at oral dose of 2 to 4 mg pitavastatin once daily for effective treatment result. In additional, we would like to demonstrate the efficacy and safety of 2 mg of pitavastatin versus 10 mg of atorvastatin.

7.4 Treatment Assignment

Patients becoming eligible at Visit 2 will be assigned a random number and each random number corresponds to a treatment group. Two treatment groups are equally distributed in two-arm study design, atorvastatin versus pitavastatin in 1:1 proportion. The random codes will be generated based on permuted block randomization method and stratified by DM status.

Note: Type 2 DM with at least one of the following description: fasting glucose > 126 mg/dL, taking OHA, HbA1c ≥ 6.5%, and random plasma glucose ≥ 200 mg/dL twice in a row according to ADA criteria. Group that did not include any mentioned-above criteria will be stratified to non-DM group.

7.5 Packaging and Labeling

Both of the pitavastatin and atorvastatin will be packaged as encapsulated for oral administration.

Capsules will be placed in a bottle for each dispensation at Visit 2, Visit 3 and Visit 4. All bottles will be labeled for study number, center number, patient identifier (patient No. and patient initials), batch number (lot number), prescription instructions, investigator’s name, storage conditions, manufacturing and expiry dates, sponsor name and address, and the warning clauses of “Clinical trials use only” and “Keep the reach out of children”. The label on the bottle will be made in duplicate with a perforated line to separate the two duplicates. One duplicate is detachable and is to be removed from the bottle at the time of drug dispensation to the patient. This removed label should be placed on the appropriate page of CRF.

The quantity of capsules packaged in the bottles for Visit 2 is 1 pack of 33 capsules,

for Visit 3 and Visit 4, 1 pack of 38 capsules will be prepared for dispensing.

The packaging of the study medication will be different if system like VCRO interactive Voice Response System (VCROIVRS) were to be employed, where each individual bottle will be labeled with a pre-assigned number randomly generated by the computer. A set of assigned numbers corresponding to the number of bottles required for that patient will be given to the site once the eligibility of the patient has been confirmed. The clinical trial pharmacist will then pick up bottles with assigned numbers for Contract Research Coordinator (CRC) to administer the study medicine to the patient.

7.6 Handling and Storage

The IP should be stored at a dry place at temperature and keep away from direct light exposure and excessive humidity. The IP will be shipped to the study site by Tai Tien Pharmaceuticals Co., Ltd. through express delivery with stable temperature service provided all the way.

The study investigator and/or the designated pharmacist will be in charge of the management and dispensation of the IP.

7.7 Product Accountability

The investigational product must not be used for the purposes other than this trial, and the clinical trial pharmacist must record the quantity supplied by the company, the amount used & backlog, and turn-in quantity on the log sheet for drug inventory and drug accountability purposes.

The quantity of investigational products given to the site will be recorded in the CRF and the drug accountability log. The used and unused investigated products will be counted at site and these records will be recorded in the CRF.

The investigator must retain all unused or expired product and accounts of any product accidentally or deliberately destroyed until the study monitor has confirmed accountability data. At the completion of the study, there will be a final reconciliation of drug shipped, drug consumed, and drug remaining. This reconciliation will be logged on the drug reconciliation form, signed and dated. Any discrepancies noted will be investigated, resolved, and documented prior to return or destruction of unused study drug.

7.8 Assessment of Compliance

Patient’s treatment compliance will be assessed by the following formula:

For each visit, the compliance will be calculated:

[(Capsules actually used) ÷ (number of days patients exposed to IP × planned number of capsules per day)]

The number of days patients exposed to IP is calculated as:

[Day of last dose — Day of first dose + 1]

7.9 Treatment for Investigational Product Overdose

Overdose of either pitavastatin or atorvastatin patient will be treated by supportive treatment. According to up to dated clinical studies, there is no known specific treatment in the event of overdose of pitavastatin or atorvastatin. In the event of overdose, the patient should be treated symptomatically and supportive measures instituted as required. Hemodialysis is unlikely to be of benefit due to high protein binding ratio of pitavastatin as well atorvastatin.

Patient or the person caring for the patient should contact the investigator by phone to determine if there is any immediate medical treatment needed. Regardless the decision of the need for immediate hospital medical care for the overdose event, the patient should be arranged to come back to the clinic as soon as possible for evaluation before continuing any further study medication treatment. This post-overdose visit can be an additional visit or a regular study visit. The sponsor's appointed study monitor should be contacted by the investigator of any over-dose report as soon as he/she is aware of the event.

The investigator should consider the following actions to take for the post-overdose visit:

1. Ask the patient to come back to the clinic and observe the overdosed lesions to see if there is any further medical treatment needed for resolving the complications induced by the overdose of study medication.
2. To determine if the patient is suitable to continue the study treatment and when the patient should resume his/her dose of study medication.
3. To determine whether or not the patient be withdrawn from the study due to reasons listed in **Section 5.2.3**.
4. Perform all study procedures required for Visit 5.

Any unfavorable effects caused by the overdose event must be reported as an AE or SAE.

8. Concomitant Treatments

8.1 Permitted Treatments

The investigators should try to minimize the concomitant treatments for the patient during the study. If the concomitant treatments are deemed necessary, the investigators should try to maintain stable dose and therapy type during the study to minimize possible interference to the study endpoint assessments.

Any use of concomitant treatment must be recorded on the CRF.

8.2 Prohibited Treatments

The following medications were identified as having the potential to interfere with the evaluation and interpretation of the results of the study and were therefore excluded.

Patients receiving medications listed below will be excluded from the study:

1. All agents for lowering/modifying plasma lipid levels, including statins, fibric acid derivatives, bile acid sequestrants, cholesterol absorption inhibitors (i.e., ezetimibe), and nicotinic acid > 500 mg/day. Patients on these medications could participate in the study, provided treatment was interrupted at least 4 weeks prior to Visit 1.
2. Oral contraceptive or any systemic steroid hormones (e.g., estrogens, progestins, androgens or glucocorticoids) for any condition, except for continuous administration of estrogen/progesterone replacement therapy which must have been constant for at least the last 3 months prior to study Visit 1. Patients on systemic steroidal treatment could be included in this study trial if the treatment was discontinued at least 4 weeks prior to Visit 1. Steroid hormones administered topically or as inhalers were permitted.
3. Anticoagulants (i.e., warfarin) and antiplatelet drugs, excluded aspirin, ticlopidine and clopidogrel in stable doses.
4. Antiarrhythmics (e.g., adenosine, amiodarone, bretylium, disopyramide, disopyramide, and flecainide, etc)
5. HIV protease inhibitors (e.g., saquinavir, ritonavir, indinavir, nelfnavir, and amprenavir, etc)
6. Immunosuppressants (e.g., azathioprine, cyclosporine, mycophenolate mofetil, sirolimus, and sodium mycophenolate, etc).
7. Macrolide (e.g., erythromycin, clarithromycin, telithromycin and josamycin). Brief systemic or topical courses of these macrolides for sporadic infections/illness may be allowed to carry the study trial.
8. Systemic azole antifungal agents (e.g., itraconazole or ketoconazole)
9. Selective serotonin reuptake inhibitors (SSRIs: citalopram, escitalopram, fluoxetine, fluvoxamine, paroxetine, and sertraline, etc)
10. Tricyclic antidepressant (TCAs: amitriptyline, clomipramine, doxepin,

imipramine, maprotiline, and nefazodone, etc)

11. Antidepressant (i.e., nefazodone)
12. Gonadotropin inhibitor (i.e., danazol)
13. Insulin or insulin analog medications
14. Other inducers of cytochrome P450 3A4 (e.g., rifampin and efavirenz)
15. Digoxin > 0.5 mg/day
16. Others: rosiglitazone, diazepam, cimetidine and omeprazole
17. Natural health foods that may interfere/modify with cholesterol and lipid profile. For example, red yeast rice, EPA/DHA, allicin, oatmeal and lycopene.

More drugs/articles are possible to be incorporated in this list as prohibited materials. There are more drugs with their elimination half-life and natural health foods that may interfere lipid profile listed in **Appendices 13.3** and **13.4**. Please refer to the Appendices for the detailed information. All prohibited medications will be listed in the patient card for any emergency use.

9. Adverse Events (AE) and Serious Adverse Events (SAE)

9.1 Definition of an AE

An AE is any untoward medical occurrence in a patient during the participation of a study, regardless the relationship of the occurrence to the treatment of the IP. An AE can be any unfavorable signs or symptoms or diagnosed new diseases or deterioration of existing chronic or intermittent diseases, significantly unexpected deteriorated condition of the study indication, which is associated to the use of the IP. Abnormal can be considered as aforementioned signs and can be considered as an AE. However, hospital admissions and/or surgical operations prescheduled prior to the participation in this study for a pre-existed illness or disease to be performed during the participation of this study are not to be considered as an AE.

The investigators are responsible for the detection, reporting, and documentation of AE.

9.2 Definition of an SAE

A serious adverse event is an AE that leads to any of the following medical occurrence:

- Results in death
- Is life-threatening, meaning the patient is at risk of death at the time of event.
- Requires hospitalization or prolongation of existing hospitalization. Emergency room visits that do not result in admission to the hospital should not be considered as a serious adverse event of requiring hospitalization or prolongation of existing hospitalization, and such emergency room visits should be evaluated for one of the other serious outcomes instead.
- Results in disability/incapacity.
- Is a congenital anomaly/birth defect.
- Events requiring medical and/or surgical intervention to prevent one of the other outcomes listed in the definition above or, based on medical and/or scientific judgment of investigator, are important and should be considered serious.

9.3 Disease-Related Events

The disease-related event is defined as an event that can be explained by the study indication. The disease-related events and conditions are usually recorded according to the protocol and should not be considered as an AE. For those events that the investigator considers to be more severe than expected, should be reported as AEs or SAEs as appropriate. Considering the nature of the study patients to be late stage cancer patients, the nature course of the disease leading to death or life-threatening conditions are considered as AEs or SAEs with investigator’s opinion to distinguish whether or not the event is a consequence or progression of the pre-existing disease(s).

For all events that result in death or are life-threatening, must be reported SAEs.

9.4 Lack of Efficacy

Lack of efficacy is not to be considered as an AE. The signs and symptoms or clinical sequelae resulting from lack of efficacy will be reported if they fulfill the definitions the AE or SAE.

9.5 Clinical Laboratory Abnormalities

Abnormal laboratory findings and/or assessments that are judged by the investigator as clinically significant will be reported as AEs or SAEs if they meet the definitions set forth. For the conditions that the abnormal laboratory and/or assessment findings are considered as the underlying disease related, and are not unexpectedly worsened during the study, no AE or SAE should be reported.

9.6 Reporting and Recording of AE and SAE

All AEs and SAEs should be documented in the source documents and the relevant CRF and SAE form when applicable. The investigator may be asked to provide photocopies of the medical records for completing the AE or SAE report. The medical records submitted to the relevant parties will be concealed of the patients' names. It is the responsibility of the investigator to report AEs or SAEs by diagnosis terminologies, if possible. When the diagnosis is possible for the reported AE or SAE, no signs and symptoms used to establish that particular diagnosis should be reported.

The investigator will be asked to determine the severity and causality of each AE and SAE based on the investigator's clinical judgment. Adverse event reporting begins from date of consent and ended at the last dose of study drug. The intensity of the AEs was graded as follows:

Mild: The AE was easily tolerated and did not interfere with normal daily activities.

Moderate: The AE caused some interference with daily activities.

Severe: The AE caused all normal daily activities to be completely halted.

The investigator made a judgment regarding whether or not the AE was related to study drug, using the definitions below. The investigator was to evaluate any changes in laboratory values and made a determination as to whether or not the change was clinically important and whether or not the changes were related to study drug. However, even if the investigator felt there was no relationship to the study drug, the AE or clinically significant laboratory abnormality must still have been recorded in the CRF along with the investigator's assessment of relationship as follows:

None: The AE must definitely have been caused by the patient's clinical state, or the study procedure/conditions (i.e. it had no association with the study drug)

Improbable: The temporal association between the AE and the study drug was such that the study drug was not likely to have any reasonable association with AE.

Possible: The AE followed a reasonable temporal sequence from the time of drug

administration, but could have been produced by the patient’s clinical state or the study procedures/conditions.

Probable: The AE followed a reasonable temporal sequence from the time of study drug administration, abated upon discontinuation of the study drug and could not be reasonably explained by the known characteristics of the patient’s clinical state.

Definite: The AE followed a reasonable temporal sequence from the time of study drug administration, abated upon discontinuation of the study drug and reappeared when the study drug is introduced.

It is usually important for the investigators to take information of underlying diseases, concomitant drugs, temporal relationship of the onset of the event to the time of dosing the IP, and re-challenging outcomes, into account when making a causal relation decision.

It is the investigators’ responsibility to follow proactively the outcome of each AE/SAE until resolution or stabilization of the condition or lost of follow-up.

Serious, alarming and/or unusual adverse events must be reported to one of the following individuals within 24 hours of the investigator's knowledge of the event:

POSITION	NAME	AFFILIA-TION	TELEPHONE NUMBER	FAX NUMBER
SAE Contact	~~Mr. Spike Lo~~<br>**Ms. Amanda Wu**<br>or<br>Ms. Yulun Liu	VCRO	886-2-2657-5399	886-2-2657-9678

An Adverse Event Form should be completed for all serious adverse events and forwarded to the sponsor within 24 hours. When new significant information is obtained as well as when outcome of an event is known, the investigator should inform the sponsor. In applicable cases, sponsor may request a letter from the investigator summarizing events related to the case. Investigator should follow patients as far as possible until an outcome to the events is known.

When a SAE is defined as a Suspected Unexpected Serious Adverse Reaction (SUSAR), the nature and severity of which is not consistent with the applicable product information (e.g., investigator’s brochure for an unapproved investigational medicinal product or package insert for an approved medicinal product). The investigator is responsible to communicate details of medical emergencies in trial patients to the Institutional Review Board (IRB). In case of a SUSAR results in death or a life-threatening event, the investigator should notify IRB at the assigned institution via a written document as soon as possible within 7 days of the initial recognition (followed by completed report within 8 days) and the other SUSARs within 15 days. Sponsor is responsible to inform the events to the regulatory authorities with the same time frame.

Under safety information exchange agreement between Tai Tien Pharmaceuticals Co., Ltd and Kowa Company, Ltd, any reportable SAE and serious adverse drug reaction (ADR) will have to report back to the Tai Tien Pharmaceuticals Co. according to

severity of the AEs in the following timeline upon receipt of the information by VCRO: 2 days for fatal or life-threatening SAEs and serious ADRs and 4 days for SAEs and serious ADRs which are not fatal/life-threatening. Tai Tien Pharmaceuticals Co. will have to reporting back to Kowa Company Ltd. on the following day according to the severity of the SAEs and the report timeline mentioned above. ADR is defined as the adverse event occurred was related to the study drug from improbable to definite causality. The SAEs and serious ADRs will be following Council for International Organizations of Medical Sciences-I (CIOMS-I) reporting form for standardized international reporting of individual cases of serious, unexpected adverse drug reactions. As for non-serious AE, including ADR and non-ADR, should be presented in the line-listing in the final clinical study report.
In addition, line-listing report form, as well called subject list, is consists of discontinued subject list, protocol deviation, demographic data, laboratory evaluations, physical examinations and other safety evaluations, adverse event listings, investigational drug administration and visit dates and comments, etc.

10. Statistical Considerations and Statistical Analysis

10.1 Sample Size Calculation

The study population consisted of male and female patients (aged 20 to 74 years) with high risk hypercholesterolemia.

Statistical justification for sample size is primarily based on the study results obtained from Phase III NK-104-301 study (see Reference 18, Study of Pitavastatin 2 mg vs. Atorvastatin 10 mg and Pitavastatin 4 mg vs. Atorvastatin 20 mg (following up-titration) in Patients with Primary Hypercholesterolemia or Combined Dyslipidemia (Protocol No.: NK-104-301). The primary endpoint utilized for this cited study is the same as the one used in this proposed study, namely, the mean percentage change of the 12th week evaluation compared to the baseline on the LDL-C blood levels of the patient. The following contents are the main statistical basis for estimating sample size for this proposed study:

The mean percentage change of the 12th week evaluation compared to the baseline on the LDL-C blood levels of the patients

1. Mean percentage change of the 12th week evaluation compared to the baseline on the LDL-C blood levels of following 2 arms based on NK-104-301 study:
   - ✓ Pitavastatin 2 mg QD
   - ✓ Atorvastatin 10 mg QD
2. Null and alternate hypotheses:

   H_0: μ_1 - $\mu_2 \leq \delta$

   H_1: μ_1 - $\mu_2 > \delta$, δ= -8.0%

   μ_1 and μ_2 denotes mean % change of Pitavastatin 2 mg QD and Atorvastatin 10 mg QD, respectively, from baseline to week 12, estimated as 37.9% (SD=13.97%) and 37.8% (SD=15.60%) in the NK-104-301 study.
3. Type I error rate: 0.05
4. Statistical power: 90%
5. Population for primary analysis in NK-104-301 study: FAS (full analysis population) population, defined as all randomized patients who receive at least one dose of study drug and who had at least one on-treatment lipid assessment. The FAS population is the same as ITT defined in this study for the primary endpoint.
7. Statistical method used to calculate the sample size: Two group Satterthwaite t-test of equal means (unequal variances) by using nQuery Advisor 7.0.
8. Interim analysis: None
9. Treatment group ratio: 1 to 1 for pitavastatin 2 mg QD versus atorvastatin 10 mg QD

Sample size derived from the above mentioned statistical conditions is 72 for each group. The evaluable patients will be determined as ITT patients with at least 1 post-treatment LDL-C measurement.

10.2 Population to be Analyzed

Patients will be categorized into the following populations to meet various study purposes.

Intention-to-treat (ITT) population: the ITT population will comprise all patients who ever received the trial medication.

Per-protocol (PP) population: the PP population is a subset of ITT who satisfy the following conditions.

1. Fulfilling all inclusion and exclusion criteria
2. Dosed with at least 75% of total study medications (= 63 capsules)
3. With primary efficacy measurement
4. Not taking any prohibited medications

Safety evaluations will be performed on ITT population while efficacy analysis will be performed on ITT and PP populations. The conclusion of efficacy of the study will be made according to the results of ITT analysis.

Summary statistics will be provided for all efficacy, safety, and baseline/demographic variables by treatment, center and DM status (2 categories only: diabetic and non-diabetic). For categorical variables, frequency tables including percentages will be presented. For continuous variables, descriptive statistics such as number of available observations, mean, median, standard deviation (SD), inter-quartile range (IQR), minimum, and maximum will be tabulated.

All statistical tests will be two-tailed with α=0.05.

10.3 Background and Demographic Characteristics

Demography and baseline characteristics including gender, age, height, weight and BMI will be listed by treatment group and analyzed between treatments to ensure comparability by using appropriate statistical methods of either ANOVA or other non-parametric tests incorporating treatment and DM status as factors for continuous variables or using appropriate statistical methods of CMH (Cochran-Mantel-Haenszel) test incorporating DM status as stratifications for categorical variables.

10.4 Efficacy Parameters

10.4.1 Primary Endpoint

Mean percentage change of LDL-C will be analyzed by using ANCOVA incorporating treatment, DM status and risk factors defined in inclusion criteria 4 (if appropriate, including condition of existing CHD and other risk factors to be determined in statistical analysis plan (SAP)) as factors and the baseline value as covariate. The proportion of patients achieving a target of lowering LDL-C will be analyzed by using CMH (Cochran-Mantel-Haenszel) test incorporating DM status as stratifications.

The hypothesis on primary endpoint will be

H_0: $\mu_1 - \mu_2 \leq \delta$

H_1: $\mu_1 - \mu_2 > \delta$, δ= -8.0%

μ_1 and μ_2 denotes mean % change of Pitavastatin 2 mg QD and Atorvastatin 10 mg QD, respectively, from baseline to week 12. Treatment group will be declared non-inferiority if the lower limit of the 95% two-sided confidence interval of the adjust means between treatment difference is greater than -8.0%.

Subgroup analysis of primary endpoints will be also performed on DM and non-DM groups, respectively.

10.4.2 Secondary Endpoints

Efficacy

The secondary efficacy endpoints will be:

Lipid efficacy

1. To estimate the proportion of patients achieving LDL-C blood level to < 100 mg/dL at Visit 5.
2. The mean percentage change of LDL-C blood level comparing baseline to week-4 (Visit 3).
3. The mean percentage changes of HDL-C blood level comparing baseline to week-4 (Visit 3) and week-12 (Visit 5).
4. The mean percentage changes of TG blood level comparing baseline to week-4 (Visit 3) and week-12 (Visit 5).
5. The mean percentage changes of non-HDL cholesterol blood level comparing baseline to week-4 (Visit 3) and week-12 (Visit 5) (non-HDL-C = TC – HDL-C).
6. To evaluate the net changes of Apolipoprotein A1 and Apolipoprotein B comparing baseline to Visit 5.

Non-lipid efficacy

1. To evaluate the net changes of fasting plasma glucose from the baseline to the week-4 (Visit 3) and week-12 (Visit 5).
2. To evaluate the net changes of fasting insulin level from the baseline to the week-12 (Visit 5).
3. To evaluate the baseline and Visit 5 insulin resistance by the homeostasis model assessment method (HOMA-IR), in equation of:

$$\text{HOMA-IR} = \frac{\text{fasting insulin level } (\mu\text{U/ml}) \times \text{fasting plasma glucose (mg/dL)}}{405 \text{ (mmol/L)}}$$

4. To evaluate the net change of HbA1c comparing the baseline to the week-12 (Visit 5).

5. To evaluate the net change of free fatty acid comparing the baseline to the week-12 (Visit 5).
6. To evaluate the net change of asymmetric dimethylarginine (ADMA) from the baseline to the week-12 (Visit 5).

Mean percentage change of LDL-C, HDL-C, TG and non-HDL-C will be analyzed by using ANCOVA incorporating treatment, DM status and risk factors defined in inclusion criteria 4 (if appropriate, including condition of existing CHD and other risk factors to be determined in SAP) as factors and the baseline value as covariate. The proportion of patients achieving a target of lowering LDL-C will be analyzed by using CMH (Cochran-Mantel-Haenszel) test incorporating DM status as stratifications.

Changes in Apo A1, Apo B, free plasma glucose, insulin level, HOMA-IR, HbA1c, free fatty acid, ADMA will be analyzed by using ANCOVA incorporating treatment, DM status and risk factors defined in inclusion criteria 4 (if appropriate, to be determined in SAP) as factors and the baseline value as covariate.

Subgroup analysis of all efficacy endpoints will be also performed on DM and non-DM groups, respectively.

<u>Safety</u>

Clinical safety

1. To evaluate the changes in physical examination results.
2. To evaluate the changes in vital signs (Pulse, systolic and diastolic blood pressures).
3. To evaluate the changes in urinalysis, including pH, erythrocyte, leukocyte, glucose, and protein. (Qualitatively)
4. To evaluate the change of EKG results (including PR, QRS, QT, QTc, RR intervals)

Laboratory safety

1. To evaluate the net change of safety profile from baseline to each visit by the follow laboratory tests:

   - White blood cell (WBC) with differential counts
   - Red blood cell (RBC)
   - Hemoglobin (Hb)
   - Hematocrit (Hct)
   - Platelet (PLT)
   - C-reactive protein (CRP)
   - Creatinine kinase (CK)
   - Aspartate Aminotransferase (AST)
   - Alanine Aminotransferase (ALT)
   - γ-glutamyl transpeptidase (GGT)
   - Total bilirubin
   - BUN

- Serum creatinine
- Lactate dehydrogenase (LDH)

2. To evaluate adverse events incidences.

Changes in vital signs, EKG (including PR, QRS, QT, QTc, RR intervals), CBC tests, biochemistry tests will be analyzed by using ANCOVA incorporating treatment and DM status as factors and the baseline value as covariate. Urinalysis will be presented with descriptive statistics to demonstrate the trend of change.

Number of patients with physical exam abnormality at each scheduled visit will be tabulated by body system. EKG examination results will be displayed in descriptive statistics with number of patients with abnormal findings tabulated for each schedule visit.

Adverse event incidents will be summarized descriptively by system organ class and preferred term using the MedDRA and by treatment group. The severity grade, relationship, action taken and outcome will also be analyzed.

10.5 Concomitant Therapy and Medical History

General medical history will be presented in incidence table by system, center, DM status and treatment listed for each patient. Ongoing medical conditions will be presented separately from the cured diseases.

The incidences of using the medications will be computed by center, DM status and treatment.

10.5.1 Compliance/Extend of Exposure

Patients' compliance will be calculated according to the formula described in **Section 7.8 Assessment of Compliance**.

The compliance will be presented descriptively. Days of patients exposing to the study medication will be calculated from the day of the first treatment to the day of last dose. Summary statistics will be provided for the days of exposure.

10.5.2 Multi-Center Study

This study will be conducted in 6 centers over north, central and southern Taiwan. By center descriptive statistics will be presented.

10.5.3 Missing Values

Last observation carry forward (LOCF) will be used for estimation of missing primary endpoint, no missing value estimation technique will be utilized for other variables.

10.5.4 Interim Analysis

Interim analysis will not be performed during the study.

11 Study Administrative Structure

11.1 Informed Consent and Institutional Review

All patients in this research study should be completely informed about the purpose, duration and important procedural details of the study. Informed Consent must be obtained using a written form which has been approved by the appropriate Independent Ethics Committee (IEC) or Institutional Review Board (IRB).

The Informed Consent Form will be understood and signed by each patient prior to enrollment into the study. The investigator will keep the original signed copies of all consent forms in his/her files and will provide a duplicate copy to the patient. A copy of the letter indicating IEC or IRB approval must be provided to the study sponsor prior to the study initiations.

11.2 Confidentiality/Publication of the Study

Any information the study sponsor shares with you regarding this study, including this protocol, is considered proprietary information and should be kept confidential. Each investigator will be required to sign the signature page which affirms he/she has read/understood the obligations of Investigators. Once the study has begun, the investigator will comply with the protocol as written except in instances where are with medical urgency. The sponsor and the IEC/IRB should be notified of these cases as soon as possible. Any other changes on the protocol must be approved by both the sponsor and the IEC/IRB prior to commencement.

The information provided by the study sponsor in this protocol and the associated Clinical Investigator's Brochure (CIB) and the data generated by this clinical study are to be considered as confidential property of the study sponsor. The data and information associated with this study may be used by the study sponsor now and in the future for the purposes of presentation, publication at discretion of the study sponsor or for submission to regulatory agencies. In addition, relative to the release of any proprietary information, the study sponsor reserves the right of prior review of any publication or presentation of data from this study.

In signing this protocol, the investigator agrees to the release of the data from this study and acknowledges the above publication policy.

11.3 Record Keeping

11.3.1 Data Collection

The clinical investigator must maintain detailed records on all study patients. Data for this study will be recorded on Case Report Forms (CRFs) provided by the sponsor. All data should be recorded completely, promptly, and legibly on the CRFs. Corrections should be made in a manner that does not obscure or eliminate the original error, by striking through the original data with one thin line, and all corrections on the CRF should be initialed and dated and reason of correction noted if possible. Each original CRF page should be made available to the sponsor when completed. The investigator should maintain a copy of all completed CRFs in the study files.

11.3.2 Drug Accountability

The principal investigator is to keep a current record of the inventory and dispensing of all test drugs. This record will be made available to the sponsor monitor for the purpose of drug accountability checks. Any significant discrepancy and/or deficiency should be recorded, with an explanation. All supplies to be sent to the investigator must be accounted for and the test material must not be used in any unauthorized situation.

The principal investigator, upon receipt of the clinical supplies, will conduct drug inventory records. The drug inventory record should be returned to the sponsor and be maintained for the investigator's records.

The principal investigator's responsibility should return all unused supplies to the sponsor at the completion of the study.

11.3.3 Record Retention

All records relating to the conduct of this study are to be held by the investigator until advised by the study sponsor that the records may be destroyed.

The investigator will allow representatives of sponsor’s monitoring members (and of the applicable regulatory authorities) to inspect all study records, CRFs, and corresponding portions of the study patient's office and/or hospital medical records at regular intervals across the study. These inspections are for the purpose of verifying the adherence to the protocol, the completeness and accuracy of the data being filled in the CRF, and compliance with applicable regulations.

Sponsor and the investigator agree that the study patient medical records will be maintained in a confidential manner. Study report will not identify any patient by name. These reports may be submitted to the Department of Health of Republic of China and any other applicable health authorities by the sponsor.

12 References

1. Arai H, Hiro T, Kimura T, Morimoto T, Miyauchi K, Nakagawa Y, Yamagishi M, Ozaki Y, Kimura K, Saito S, Yamaguchi T, Daida H, Matsuzaki M. More Intensive Lipid Lowering is Associated with Regression of Coronary Atherosclerosis in Diabetic Patients with Acute Coronary Syndrome. *J Atheroscler Thromb. 2010 Jul 28.* [Epub ahead of print]
2. Yokote K, Saito Y; CHIBA. Influence of statins on glucose tolerance in patients with type 2 diabetes mellitus: subanalysis of the collaborative study on hypercholesterolemia drug intervention and their benefits for atherosclerosis prevention (CHIBA study). *J Atheroscler Thromb. 2009 Jun;16(3):297-8. Epub 2009 Jun 26.*
3. Charlton-Menys V, Betteridge DJ, Colhoun H, Fuller J, France M, Hitman GA, Livingstone SJ, Neil HA, Newman CB, Szarek M, DeMicco DA, Durrington PN. Apolipoproteins, cardiovascular risk and statin response in type 2 diabetes: the Collaborative Atorvastatin Diabetes Study (CARDS). *Diabetologia. 2009 Feb;52(2):218-25. Epub 2008 Oct 30.*
4. Shepherd J, Barter P, Carmena R, Deedwania P, Fruchart JC, Haffner S, Hsia J, Breazna A, LaRosa J, Grundy S, Waters D. Effect of lowering LDL cholesterol substantially below currently recommended levels in patients with coronary heart disease and diabetes: the Treating to New Targets (TNT) study. *Diabetes Care. 2006 Jun;29(6):1220-6.*
5. Tokuno A, Hirano T, Hayashi T, Mori Y, Yamamoto T, Nagashima M, Shiraishi Y, Ito Y, Adachi M. The effects of statin and fibrate on lowering small dense LDL-cholesterol in hyperlipidemic patients with type 2 diabetes. *J Atheroscler Thromb. 2007 Jun;14(3):128-32.*
6. Ballantyne CM, Raichlen JS, Cain VA. Statin therapy alters the relationship between apolipoprotein B and low-density lipoprotein cholesterol and non-high-density lipoprotein cholesterol targets in high-risk patients: the MERCURY II (Measuring Effective Reductions in Cholesterol Using Rosuvastatin) trial. *J Am Coll Cardiol. 2008 Aug 19;52(8):626-32.*
7. Conard S, Bays H, Leiter LA, Bird S, Lin J, Hanson ME, Shah A, Tershakovec AM. Ezetimibe added to atorvastatin compared with doubling the atorvastatin dose in patients at high risk for coronary heart disease with diabetes mellitus, metabolic syndrome or neither. *Diabetes Obes Metab. 2010 Mar;12(3):210-8.*
8. Kawai T, Tokui M, Funae O, Meguro S, Yamada S, Tabata M, Shimada A. Efficacy of pitavastatin, a new HMG-CoA reductase inhibitor, on lipid and glucose metabolism in patients with type 2 diabetes. *Diabetes Care. 2005 Dec;28(12):2980-1.*
9. Bybee KA, Lee JH, O'Keefe JH. Cumulative clinical trial data on atorvastatin for reducing cardiovascular events: the clinical impact of atorvastatin. *Curr Med Res Opin. 2008 Apr;24(4):1217-29. Epub 2008 Mar 20.*
10. Athyros VG, Tziomalos K, Karagiannis A, Mikhailidis DP. Atorvastatin: safety

and tolerability. *Expert Opin Drug Saf. 2010 Jul;9(4):667-74.*

11. David J. Graham, MD, MPH; Judy A. Staffa, PhD; Deborah Shatin, PhD; Susan E. Andrade, ScD; Stephanie D. Schech, MPH; Lois La Grenade, MD, MPH; Jerry H. Gurwitz, MD; K. Arnold Chan, MD, ScD; Michael J. Goodman, PhD; Richard Platt, MD, MSc. Incidence of Hospitalized Rhabdomyolysis in Patients Treated With Lipid-Lowering Drugs. *JAMA. 2004;292:(doi:10.1001/jama.292.21.2585)*
12. Mukhtar RY, Reid J, Reckless JP. Pitavastatin. *Int J Clin Pract. 2005 Feb;59(2):239-52.*
13. Teramoto T, Shimano H, Yokote K, Urashima M. Effects of pitavastatin (LIVALO Tablet) on high density lipoprotein cholesterol (HDL-C) in hypercholesterolemia. *J Atheroscler Thromb. 2009 Oct;16(5):654-61.*
14. Yamakawa T, Takano T, Tanaka S, Kadonosono K, Terauchi Y. Influence of pitavastatin on glucose tolerance in patients with type 2 diabetes mellitus. *J Atheroscler Thromb. 2008 Oct;15(5):269-75.*
15. Motomura T, Okamoto M, Kitamura T, Yamamoto H, Otsuki M, Asanuma N, Takagi M, Kurebayashi S, Hashimoto K, Sumitani S, Saito H, Kouhara H, Nshii K, Nakao M, Koga M, Sato B, Morimoto Y, Kasayama S. Effects of pitavastatin on serum lipids and high sensitivity C-reactive protein in type 2 diabetic patients. *J Atheroscler Thromb. 2009 Oct;16(5):546-52. Epub 2009 Sep 3.*
16. Koshiyama H, Taniguchi A, Tanaka K, Kagimoto S, Fujioka Y, Hirata K, Nakamura Y, Iwakura A, Hara K, Yamamoto T, Kuroe A, Ohya M, Fujimoto S, Hamamoto Y, Honjo S, Ikeda H, Nabe K, Tsuda K, Inagaki N, Seino Y, Kume N Effects of pitavastatin on lipid profiles and high-sensitivity CRP in Japanese patients with hypercholesterolemia: Kansai Investigation of Statin for Hyperlipidemic Intervention in Metabolism and Endocrinology (KISHIMEN) investigatars. J Atheroscler Thromb. 2008 Dec;15(6):345-50. Epub 2008 Dec 11.
17. Kurihara Y, Douzono T, Kawakita K, Nagasaki Y. A large-scale, long-term, prospective post-marketing surveillance of pitavastatin (LIVALO Tablet): LIVALO Effectiveness and Safety (LIVES) study. Jpn Pharmacol. Ther. 36, 709–731 (2008).
18. Study of pitavastatin 2 mg vs. atorvastatin 10 mg and pitavastatin 4 mg vs. atorvastatin 20 mg (following up-titration) in patients with primary hypercholesterolemia or combined dyslipidemia. protocol No.: NK-104-301, Phase III

13 Appendices

13.1 Declaration of Helsinki

World Medical Association Declaration of Helsinki: Recommendations Guiding Physicians in Biomedical Research Involving Human Subjects

Adopted by the 18th World Medical Association (WMA) General Assembly, Helsinki, Finland, 1964 and amended by the WMA General Assembly, Tokyo, Japan in 1975, Venice, Italy in 1983, Hong Kong in 1989, Somerset West, Republic of South Africa, 1996, Edinburgh, Scotland in 2000, added with Note of Clarification on paragraph 29 by the WMA General Assembly, Washington in 2002, added with Note of Clarification on paragraph 30 by the WMA General Assembly, Tokyo 2004, and amended by the WMA General Assembly, Seoul, 2008.

A. Introduction

1. The World Medical Association (WMA) has developed the Declaration of Helsinki as a statement of ethical principles for medical research involving human subjects, including research on identifiable human material and data.

   The Declaration is intended to be read as a whole and each of its constituent paragraphs should not be applied without consideration of all other relevant paragraphs.

2. Although the Declaration is addressed primarily to physicians, the WMA encourages other participants in medical research involving human subjects to adopt these principles.

3. It is the duty of the physician to promote and safeguard the health of subjects, including those who are involved in medical research. The physician's knowledge and conscience are dedicated to the fulfilment of this duty.

4. The Declaration of Geneva of the WMA binds the physician with the words, “The health of my subject will be my first consideration,” and the International Code of Medical Ethics declares that, “A physician shall act in the subject's best interest when providing medical care.”

5. Medical progress is based on research that ultimately must include studies involving human subjects. Populations that are underrepresented in medical research should be provided appropriate access to participation in research.

6. In medical research involving human subjects, the well-being of the individual research subject must take precedence over all other interests.

7. The primary purpose of medical research involving human subjects is to understand the causes, development and effects of diseases and improve preventive, diagnostic and therapeutic interventions (methods, procedures and treatments). Even the best current interventions must be evaluated continually

through research for their safety, effectiveness, efficiency, accessibility and quality.

8. In medical practice and in medical research, most interventions involve risks and burdens.

9. Medical research is subject to ethical standards that promote respect for all human subjects and protect their health and rights. Some research populations are particularly vulnerable and need special protection. These include those who cannot give or refuse consent for themselves and those who may be vulnerable to coercion or undue influence.

10. Physicians should consider the ethical, legal and regulatory norms and standards for research involving human subjects in their own countries as well as applicable international norms and standards. No national or international ethical, legal or regulatory requirement should reduce or eliminate any of the protections for research subjects set forth in this Declaration.

B. PRINCIPLES FOR ALL MEDICAL RESEARCH

11. It is the duty of physicians who participate in medical research to protect the life, health, dignity, integrity, right to self-determination, privacy, and confidentiality of personal information of research subjects.

12. Medical research involving human subjects must conform to generally accepted scientific principles, be based on a thorough knowledge of the scientific literature, other relevant sources of information, and adequate laboratory and, as appropriate, animal experimentation. The welfare of animals used for research must be respected.

13. Appropriate caution must be exercised in the conduct of medical research that may harm the environment.

14. The design and performance of each research study involving human subjects must be clearly described in a research protocol. The protocol should contain a statement of the ethical considerations involved and should indicate how the principles in this Declaration have been addressed. The protocol should include information regarding funding, sponsors, institutional affiliations, other potential conflicts of interest, incentives for subjects and provisions for treating and/or compensating subjects who are harmed as a consequence of participation in the research study. The protocol should describe arrangements for post-study access by study subjects to interventions identified as beneficial in the study or access to other appropriate care or benefits.

15. The research protocol must be submitted for consideration, comment, guidance and approval to a research ethics committee before the study begins. This committee must be independent of the researcher, the sponsor and any other undue influence. It must take into consideration the laws and regulations of the country or countries in which the research is to be performed as well as applicable international norms and standards but these must not be allowed to reduce or eliminate any of the protections for research subjects set forth in this Declaration. The committee must have the right to monitor ongoing studies.

The researcher must provide monitoring information to the committee, especially information about any serious adverse events. No change to the protocol may be made without consideration and approval by the committee.

16. Medical research involving human subjects must be conducted only by individuals with the appropriate scientific training and qualifications. Research on subjects or healthy volunteers requires the supervision of a competent and appropriately qualified physician or other health care professional. The responsibility for the protection of research subjects must always rest with the physician or other health care professional and never the research subjects, even though they have given consent.
17. Medical research involving a disadvantaged or vulnerable population or community is only justified if the research is responsive to the health needs and priorities of this population or community and if there is a reasonable likelihood that this population or community stands to benefit from the results of the research.
18. Every medical research study involving human subjects must be preceded by careful assessment of predictable risks and burdens to the individuals and communities involved in the research in comparison with foreseeable benefits to them and to other individuals or communities affected by the condition under investigation.
19. Every clinical trial must be registered in a publicly accessible database before recruitment of the first subject.
20. Physicians may not participate in a research study involving human subjects unless they are confident that the risks involved have been adequately assessed and can be satisfactorily managed. Physicians must immediately stop a study when the risks are found to outweigh the potential benefits or when there is conclusive proof of positive and beneficial results.
21. Medical research involving human subjects may only be conducted if the importance of the objective outweighs the inherent risks and burdens to the research subjects.
22. Participation by competent individuals as subjects in medical research must be voluntary. Although it may be appropriate to consult family members or community leaders, no competent individual may be enrolled in a research study unless he or she freely agrees.
23. Every precaution must be taken to protect the privacy of research subjects and the confidentiality of their personal information and to minimize the impact of the study on their physical, mental and social integrity.
24. In medical research involving competent human subjects, each potential subject must be adequately informed of the aims, methods, sources of funding, any possible conflicts of interest, institutional affiliations of the researcher, the anticipated benefits and potential risks of the study and the discomfort it may entail, and any other relevant aspects of the study. The potential subject must be informed of the right to refuse to participate in the study or to withdraw

consent to participate at any time without reprisal. Special attention should be given to the specific information needs of individual potential subjects as well as to the methods used to deliver the information. After ensuring that the potential subject has understood the information, the physician or another appropriately qualified individual must then seek the potential subject's freely-given informed consent, preferably in writing. If the consent cannot be expressed in writing, the non-written consent must be formally documented and witnessed.

25. For medical research using identifiable human material or data, physicians must normally seek consent for the collection, analysis, storage and/or reuse. There may be situations where consent would be impossible or impractical to obtain for such research or would pose a threat to the validity of the research. In such situations the research may be done only after consideration and approval of a research ethics committee.

26. When seeking informed consent for participation in a research study the physician should be particularly cautious if the potential subject is in a dependent relationship with the physician or may consent under duress. In such situations the informed consent should be sought by an appropriately qualified individual who is completely independent of this relationship.

27. For a potential research subject who is incompetent, the physician must seek informed consent from the legally authorized representative. These individuals must not be included in a research study that has no likelihood of benefit for them unless it is intended to promote the health of the population represented by the potential subject, the research cannot instead be performed with competent persons, and the research entails only minimal risk and minimal burden.

28. When a potential research subject who is deemed incompetent is able to give assent to decisions about participation in research, the physician must seek that assent in addition to the consent of the legally authorized representative. The potential subject's dissent should be respected.

29. Research involving subjects who are physically or mentally incapable of giving consent, for example, unconscious subjects, may be done only if the physical or mental condition that prevents giving informed consent is a necessary characteristic of the research population. In such circumstances the physician should seek informed consent from the legally authorized representative. If no such representative is available and if the research cannot be delayed, the study may proceed without informed consent provided that the specific reasons for involving subjects with a condition that renders them unable to give informed consent have been stated in the research protocol and the study has been approved by a research ethics committee. Consent to remain in the research should be obtained as soon as possible from the subject or a legally authorized representative.

30. Authors, editors and publishers all have ethical obligations with regard to the publication of the results of research. Authors have a duty to make publicly

available the results of their research on human subjects and are accountable for the completeness and accuracy of their reports. They should adhere to accepted guidelines for ethical reporting. Negative and inconclusive as well as positive results should be published or otherwise made publicly available. Sources of funding, institutional affiliations and conflicts of interest should be declared in the publication. Reports of research not in accordance with the principles of this Declaration should not be accepted for publication.

C. ADDITIONAL PRINCIPLES FOR MEDICAL RESEARCH COMBINED WITH

MEDICAL CARE

31. The physician may combine medical research with medical care only to the extent that the research is justified by its potential preventive, diagnostic or therapeutic value and if the physician has good reason to believe that participation in the research study will not adversely affect the health of the subjects who serve as research subjects.

32. The benefits, risks, burdens and effectiveness of a new intervention must be tested against those of the best current proven intervention, except in the following circumstances:

    • The use of placebo, or no treatment, is acceptable in studies where no current proven intervention exists; or

    • Where for compelling and scientifically sound methodological reasons the use of placebo is necessary to determine the efficacy or safety of an intervention and the subjects who receive placebo or no treatment will not be subject to any risk of serious or irreversible harm. Extreme care must be taken to avoid abuse of this option.

33. At the conclusion of the study, subjects entered into the study are entitled to be informed about the outcome of the study and to share any benefits that result from it, for example, access to interventions identified as beneficial in the study or to other appropriate care or benefits.

34. The physician must fully inform the subject which aspects of the care are related to the research. The refusal of a subject to participate in a study or the subject's decision to withdraw from the study must never interfere with the subject-physician relationship.

35. In the treatment of a subject, where proven interventions do not exist or have been ineffective, the physician, after seeking expert advice, with informed consent from the subject or a legally authorized representative, may use an unproven intervention if in the physician's judgement it offers hope of saving life, re-establishing health or alleviating suffering. Where possible, this intervention should be made the object of research, designed to evaluate its safety and efficacy. In all cases, new information should be recorded and, where appropriate, made publicly available.

13.2 ATP III Guidelines

National Cholesterol Education Program

ATP III Guidelines At-A-Glance Quick Desk Reference

Step 1 **Determine lipoprotein levels–obtain complete lipoprotein profile after 9- to 12-hour fast.**

ATP III Classification of LDL, Total, and HDL Cholesterol (mg/dL)

**LDL Cholesterol – Primary Target of Therapy**	
<100	Optimal
100-129	Near optimal/above optimal
130-159	Borderline high
160-189	High
≥190	Very high
**Total Cholesterol**	
<200	Desirable
200-239	Borderline high
≥240	High
**HDL Cholesterol**	
<40	Low
≥60	High

Step 2 **Identify presence of clinical atherosclerotic disease that confers high risk for coronary heart disease (CHD) events (CHD risk equivalent):**

- Clinical CHD
- Symptomatic carotid artery disease
- Peripheral arterial disease
- Abdominal aortic aneurysm.

Step 3 **Determine presence of major risk factors (other than LDL):**

Major Risk Factors (Exclusive of LDL Cholesterol) That Modify LDL Goals

Cigarette smoking
Hypertension (BP ≥140/90 mmHg or on antihypertensive medication)
Low HDL cholesterol (<40 mg/dL)*
Family history of premature CHD (CHD in male first degree relative <55 years; CHD in female first degree relative <65 years)
Age (men ≥45 years; women ≥55 years)

** HDL cholesterol ≥60 mg/dL counts as a "negative" risk factor; its presence removes one risk factor from the total count.*

- Note: in ATP III, diabetes is regarded as a CHD risk equivalent.

High Blood Cholesterol

NATIONAL INSTITUTES OF HEALTH
NATIONAL HEART, LUNG, AND BLOOD INSTITUTE

4
Step 4

If 2+ risk factors (other than LDL) are present without CHD or CHD risk equivalent, assess 10-year (short-term) CHD risk (see Framingham tables).
Three levels of 10-year risk:

- >20% — CHD risk equivalent
- 10-20%
- <10%

5
Step 5

Determine risk category:

- Establish LDL goal of therapy
- Determine need for therapeutic lifestyle changes (TLC)
- Determine level for drug consideration

LDL Cholesterol Goals and Cutpoints for Therapeutic Lifestyle Changes (TLC) and Drug Therapy in Different Risk Categories.

Risk Category	LDL Goal	LDL Level at Which to Initiate Therapeutic Lifestyle Changes (TLC)	LDL Level at Which to Consider Drug Therapy
CHD or CHD Risk Equivalents (10-year risk >20%)	<100 mg/dL	≥100 mg/dL	≥130 mg/dL (100-129 mg/dL: drug optional)*
2+ Risk Factors (10-year risk ≤20%)	<130 mg/dL	≥130 mg/dL	10-year risk 10-20%: ≥130 mg/dL<br>10-year risk <10%: ≥160 mg/dL
0-1 Risk Factor†	<160 mg/dL	≥160 mg/dL	≥190 mg/dL (160-189 mg/dL: LDL-lowering drug optional)

* *Some authorities recommend use of LDL-lowering drugs in this category if an LDL cholesterol <100 mg/dL cannot be achieved by therapeutic lifestyle changes. Others prefer use of drugs that primarily modify triglycerides and HDL, e.g., nicotinic acid or fibrate. Clinical judgment also may call for deferring drug therapy in this subcategory.*
† *Almost all people with 0-1 risk factor have a 10-year risk <10%, thus 10-year risk assessment in people with 0-1 risk factor is not necessary.*

6
Step 6

Initiate therapeutic lifestyle changes (TLC) if LDL is above goal.

TLC Features

- TLC Diet:
  - Saturated fat <7% of calories, cholesterol <200 mg/day
  - Consider increased viscous (soluble) fiber (10-25 g/day) and plant stanols/sterols (2g/day) as therapeutic options to enhance LDL lowering
- Weight management
- Increased physical activity.

7

Step 7

Consider adding drug therapy if LDL exceeds levels shown in Step 5 table:

- Consider drug simultaneously with TLC for CHD and CHD equivalents
- Consider adding drug to TLC after 3 months for other risk categories.

Drugs Affecting Lipoprotein Metabolism

Drug Class	Agents and Daily Doses	Lipid/Lipoprotein Effects	Side Effects	Contraindications
HMG CoA reductase inhibitors (statins)	Lovastatin (20-80 mg)<br>Pravastatin (20-40 mg)<br>Simvastatin (20-80 mg)<br>Fluvastatin (20-80 mg)<br>Atorvastatin (10-80 mg)<br>Cerivastatin (0.4-0.8 mg)	LDL ↓18-55%<br>HDL ↑5-15%<br>TG ↓7-30%	Myopathy<br>Increased liver enzymes	Absolute:<br>• Active or chronic liver disease<br>Relative:<br>• Concomitant use of certain drugs*
Bile acid sequestrants	Cholestyramine (4-16 g)<br>Colestipol (5-20 g)<br>Colesevelam (2.6-3.8 g)	LDL ↓15-30%<br>HDL ↑3-5%<br>TG No change or increase	Gastrointestinal distress<br>Constipation<br>Decreased absorption of other drugs	Absolute:<br>• dysbeta-lipoproteinemia<br>• TG >400 mg/dL<br>Relative:<br>• TG >200 mg/dL
Nicotinic acid	Immediate release (crystalline) nicotinic acid (1.5-3 gm), extended release nicotinic acid (Niaspan®) (1-2 g), sustained release nicotinic acid (1-2 g)	LDL ↓5-25%<br>HDL ↑15-35%<br>TG ↓20-50%	Flushing<br>Hyperglycemia<br>Hyperuricemia (or gout)<br>Upper GI distress<br>Hepatotoxicity	Absolute:<br>• Chronic liver disease<br>• Severe gout<br>Relative:<br>• Diabetes<br>• Hyperuricemia<br>• Peptic ulcer disease
Fibric acids	Gemfibrozil (600 mg BID)<br>Fenofibrate (200 mg)<br>Clofibrate (1000 mg BID)	LDL ↓5-20%<br>*(may be increased in patients with high TG)*<br>HDL ↑10-20%<br>TG ↓20-50%	Dyspepsia<br>Gallstones<br>Myopathy	Absolute:<br>• Severe renal disease<br>• Severe hepatic disease

* *Cyclosporine, macrolide antibiotics, various anti-fungal agents, and cytochrome P-450 inhibitors (fibrates and niacin should be used with appropriate caution).*

8

Step 8

Identify metabolic syndrome and treat, if present, after 3 months of TLC.

Clinical Identification of the Metabolic Syndrome – Any 3 of the Following:

Risk Factor	Defining Level
Abdominal obesity*	Waist circumference†
Men	>102 cm (>40 in)
Women	>88 cm (>35 in)
Triglycerides	≥150 mg/dL
HDL cholesterol	
Men	<40 mg/dL
Women	<50 mg/dL
Blood pressure	≥130/≥85 mmHg
Fasting glucose	≥110 mg/dL

* *Overweight and obesity are associated with insulin resistance and the metabolic syndrome. However, the presence of abdominal obesity is more highly correlated with the metabolic risk factors than is an elevated body mass index (BMI). Therefore, the simple measure of waist circumference is recommended to identify the body weight component of the metabolic syndrome.*

† *Some male patients can develop multiple metabolic risk factors when the waist circumference is only marginally increased, e.g., 94-102 cm (37-39 in). Such patients may have a strong genetic contribution to insulin resistance. They should benefit from changes in life habits, similarly to men with categorical increases in waist circumference.*

Treatment of the metabolic syndrome

- Treat underlying causes (overweight/obesity and physical inactivity):
  - Intensify weight management
  - Increase physical activity.
- Treat lipid and non-lipid risk factors if they persist despite these lifestyle therapies:
  - Treat hypertension
  - Use aspirin for CHD patients to reduce prothrombotic state
  - Treat elevated triglycerides and/or low HDL (as shown in Step 9).

9

Step 9

Treat elevated triglycerides.

ATP III Classification of Serum Triglycerides (mg/dL)

<150	Normal
150-199	Borderline high
200-499	High
≥500	Very high

Treatment of elevated triglycerides (≥150 mg/dL)

- Primary aim of therapy is to reach LDL goal
- Intensify weight management
- Increase physical activity
- If triglycerides are ≥200 mg/dL after LDL goal is reached, set secondary goal for non-HDL cholesterol (total – HDL) 30 mg/dL higher than LDL goal.

Comparison of LDL Cholesterol and Non-HDL Cholesterol Goals for Three Risk Categories

Risk Category	LDL Goal (mg/dL)	Non-HDL Goal (mg/dL)
CHD and CHD Risk Equivalent (10-year risk for CHD >20%)	<100	<130
Multiple (2+) Risk Factors and 10-year risk ≤20%	<130	<160
0-1 Risk Factor	<160	<190

If triglycerides 200-499 mg/dL after LDL goal is reached, consider adding drug if needed to reach non-HDL goal:

- intensify therapy with LDL-lowering drug, or
- add nicotinic acid or fibrate to further lower VLDL.

If triglycerides ≥500 mg/dL, first lower triglycerides to prevent pancreatitis:

- very low-fat diet (≤15% of calories from fat)
- weight management and physical activity
- fibrate or nicotinic acid
- when triglycerides <500 mg/dL, turn to LDL-lowering therapy.

Treatment of low HDL cholesterol (<40 mg/dL)

- First reach LDL goal, then:
- Intensify weight management and increase physical activity
- If triglycerides 200-499 mg/dL, achieve non-HDL goal
- If triglycerides <200 mg/dL (isolated low HDL) in CHD or CHD equivalent consider nicotinic acid or fibrate.

Men

Estimate of 10-Year Risk for Men

(Framingham Point Scores)

Age	Points
20-34	-9
35-39	-4
40-44	0
45-49	3
50-54	6
55-59	8
60-64	10
65-69	11
70-74	12
75-79	13

Total Cholesterol	Points				
	Age 20-39	Age 40-49	Age 50-59	Age 60-69	Age 70-79
<160	0	0	0	0	0
160-199	4	3	2	1	0
200-239	7	5	3	1	0
240-279	9	6	4	2	1
≥280	11	8	5	3	1

	Points				
	Age 20-39	Age 40-49	Age 50-59	Age 60-69	Age 70-79
Nonsmoker	0	0	0	0	0
Smoker	8	5	3	1	1

HDL (mg/dL)	Points
≥60	-1
50-59	0
40-49	1
<40	2

Systolic BP (mmHg)	If Untreated	If Treated
<120	0	0
120-129	0	1
130-139	1	2
140-159	1	2
≥160	2	3

Point Total	10-Year Risk %
<0	< 1
0	1
1	1
2	1
3	1
4	1
5	2
6	2
7	3
8	4
9	5
10	6
11	8
12	10
13	12
14	16
15	20
16	25
≥17	≥ 30

10-Year risk _____%

Women

Estimate of 10-Year Risk for Women

(Framingham Point Scores)

Age	Points
20-34	-7
35-39	-3
40-44	0
45-49	3
50-54	6
55-59	8
60-64	10
65-69	12
70-74	14
75-79	16

Total Cholesterol	Points				
	Age 20-39	Age 40-49	Age 50-59	Age 60-69	Age 70-79
<160	0	0	0	0	0
160-199	4	3	2	1	1
200-239	8	6	4	2	1
240-279	11	8	5	3	2
≥280	13	10	7	4	2

	Points				
	Age 20-39	Age 40-49	Age 50-59	Age 60-69	Age 70-79
Nonsmoker	0	0	0	0	0
Smoker	9	7	4	2	1

HDL (mg/dL)	Points
≥60	-1
50-59	0
40-49	1
<40	2

Systolic BP (mmHg)	If Untreated	If Treated
<120	0	0
120-129	1	3
130-139	2	4
140-159	3	5
≥160	4	6

Point Total	10-Year Risk %
< 9	< 1
9	1
10	1
11	1
12	1
13	2
14	2
15	3
16	4
17	5
18	6
19	8
20	11
21	14
22	17
23	22
24	27
≥25	≥ 30

10-Year risk _____%

U.S. DEPARTMENT OF HEALTH AND HUMAN SERVICES
Public Health Service
National Institutes of Health
National Heart, Lung, and Blood Institute

NIH Publication No. 01-3305
May 2001

13.3 Medications May Interfere with IP

Most of the drugs listed below are having $t_{1/2}$×10 (days) elimination period within 4 weeks, except Amiodarone. Therefore the wash out period was defined as 4-week before Visit 2.

Drug	t1/2 (hours)	t1/2×10 (hours)	t1/2×10 (days)
**Statins**			
Atorvastatin	20 ~ 30	200 ~ 300	8.33 ~ 12.50
Fluvastatin	2 ~ 3	20 ~ 30	0.83 ~ 1.25
Pravastatin	1.1 ~ 1.7	11 ~ 17	0.46 ~ 0.71
Rosuvastatin	1.5 ~ 2.0	15 ~ 17	0.63 ~ 0.71
Simvastatin	19	190	7.92
**Fibrates**			
Clofibrate	12 ~ 35	120 ~ 350	5.00 ~ 14.58
Fenofibrate	20	200	8.33
Gemfribrozil	1.3 ~ 1.5	13 ~ 15	0.54 ~ 0.63
**Cholesterol absorption inhibitor**			
Ezetimibe	22	220	9.17
**Niacins**			
Nicotinic acid	0.75	7.5	0.31
Niceritnol	8	80	3.33
**Steroid hormones**			
Dexamethasone	4	40	1.67
Fludrocortisone	3.5	35	1.46
Hydrocortisone	1.5 ~ 2	15 ~ 20	0.63 ~ 0.83
Methylprednisolone	3.5	35	1.46
Prednisolone	3.5	35	1.46
Triamcinolone	2 ~ 5	20 ~ 50	0.83 ~ 2.08
Estrogen	4 ~ 18.5	40 ~ 185	1.67 ~ 7.71
Progesterone	35 ~ 55	350 ~ 550	15 ~ 23
Testosterone	2 ~ 4	20 ~ 40	0.83 ~ 1.67
**Anticoagulant**			
Warfarin	0.5 ~ 3	5 ~ 30	0.21 ~ 1.25
**Antiarrhythmics**			
Adenosine	10 seconds	100 seconds	1.67 minutes
Amiodarone	2.5 ~ 10 days	25 ~ 100 days	0.83 ~ 3.33 months
Bretylium tosylate	4 ~ 17	40 ~ 170	1.67 ~ 7.08
Disopyramide	4 ~ 10	40 ~ 100	1.67 ~ 4.17
Flecainide acetate	7 ~ 22	70 ~ 220	2.92 ~ 9.17

Drug	t1/2 (hours)	t1/2×10 (hours)	t1/2×10 (days)
**Immunosuppressants**			
Azathioprine	3	30	1.25
Cyclosporine	19 ~ 27	190 ~ 270	7.92 ~ 11.25
Daclizumab	20	200	8.33
Mycophenolate Mofetil	11	110	4.58
Sirolimus	62	620	7.71
Sodium mycophenolate	11.7 ~ 15.7	117 ~ 157	4.88 ~ 6.54
**Macrolides**			
Erythromycin	1 ~ 2	10-20	0.42 ~ 0.83
Clarithromycin	7	70	2.9
Telithromycin	10	100	4.2
Josamycin	1.13	11.3	0.47
**SSRIs**			
Citalopram HBr	36	360	15.00
Escitalopram	25	250	10.42
Fluoxetine	2 ~ 3 days	20 ~ 30 days	0.67 ~ 1.00 months
Fluvoxamine Maleate	15	150	6.25
Paroxetine HCl	16 ~ 24	160 ~ 240	6.67 ~ 10.00
Sertraline HCl	26	260	10.83
**TCAs**			
Amitriptyline	20 ~ 40	200 ~ 400	8.33 ~ 16.67
Clomipramine	20 ~ 30	200 ~ 300	8.33 ~ 12.50
Doxepin HCl	6 ~ 8	60 ~ 80	2.50 ~ 3.33
Imipramine	8 ~ 16	80 ~ 160	3.33 ~ 6.67
Maprotiline HCl	51	510	21.25
**Antidepressant**			
Nefazodone	3.5	35	1.46
**Gonadotropin inhibitor**			
Danazol	4.5	45	1.88
**Others**			
Azole antifungals	30	300	12.50
Rosiglitazone	3 ~ 7	30 ~ 70	1.25 ~ 2.92
Rifampin	3	30	1.25
Efavirenz	52 ~ 76	520 ~ 760	21.67 ~ 31.67
Digoxin	34 ~ 44	340 ~ 440	14.17 ~ 18.33
Diazepam	20 ~ 50	200 ~ 500	8.33 ~ 20.83
Cimetidine	2	20	0.83
Omeprazole	0.5 ~ 1.5	5 ~ 15	0.21 ~ 0.63

13.4 Natural Health Food Products May Interfere with IP

Natural health food/ingredients may interfere with lipid profile.

Component that may reduce lipid profile	Recommended Daily Intake (RDI)
EPA+DHA	500 mg
Monacolin K	9.6 ~ 15 mg
Allicin	5000 ~ 6000 μg
Immunoglobulin G	N.A.
β-glucan	3 ~ 6.9 g
Chitosan	N.A.
Vitamin P	In ratio of Vitamin C : Vitamin P = 5 : 1
Oleic acid、Linoleic acid、α-Linoleic acid	30 ~ 45 g
Tomato juice (lycopene)	30 ~ 35 ml
Sugar Cane Policosanol	5 ~ 10 mg
Soy protein	25 g
Catechin	1.8 g
Inulin	NA
Medium-chain triglyceride	15 ~ 20 g
L-arginine	15 g
Water-solubility diet fiber	20 mg
Phytosterol	2 ~ 3 g
EPA+DHA	500 mg
Monacolin K	9.6 ~ 15 mg
