# Supplementary material for: Pitavastatin and Atorvastatin Double-Blind Randomized ComPArative Study among HiGh-Risk Patients, Including ThOse with Type 2 Diabetes Mellitus, in Taiwan (PAPAGO-T Study)
Source: PLoS One. 2013 Oct 1;8(10):e76298. doi: 10.1371/journal.pone.0076298 (PMC3788128; doi:10.1371/journal.pone.0076298)
Supplement: Protocol S2 — Trial Registration. (PDF) [file pone.0076298.s003.pdf]

ClinicalTrials.gov
A service of the U.S. National Institutes of Health

Efficacy and Safety Study of Pitavastatin and Atorvastatin in High Risk Hypercholesterolemic Patients

This study is not yet open for participant recruitment.
Verified on June 2011 by Tai Tien Pharmaceuticals Co., Ltd.

First Received on June 29, 2011. Last Updated on June 30, 2011 History of Changes

**Sponsor:**	Tai Tien Pharmaceuticals Co., Ltd.
**Information provided by:**	Tai Tien Pharmaceuticals Co., Ltd.
**ClinicalTrials.gov Identifier:**	NCT01386853

Purpose

This is a 12-week, randomized, multicenter, double-blind, active-controlled, non-inferiority study (TATPITA20101005) to compare the efficacy and safety of **pitavastatin** (**Livalo**®) and atorvastatin (Lipitor®) in high risk hypercholesterolemic patients.

Condition	Intervention	Phase
Hyperlipidemia	Drug: **Pitavastatin**<br>Drug: Atorvastatin	Phase III

Study Type: Interventional
Study Design: Allocation: Randomized
Endpoint Classification: Safety/Efficacy Study
Intervention Model: Parallel Assignment
Masking: Double Blind (Subject, Caregiver, Investigator, Outcomes Assessor)
Primary Purpose: Treatment

Official Title: A 12-week, Randomized, Multicenter, Double-blind, Active-controlled, Non-inferiority Study to Compare the Efficacy and Safety of **Pitavastatin** and Atorvastatin in High Risk Hypercholesterolemic Patients

Resource links provided by NLM:

Drug Information available for: Atorvastatin Atorvastatin calcium Pitavastatin NK 104

U.S. FDA Resources

Further study details as provided by Tai Tien Pharmaceuticals Co., Ltd.:

Primary Outcome Measures:

- The change of LDL-C [Time Frame: Baseline to 12 weeks] [Designated as safety issue: No]

Secondary Outcome Measures:

- The proportion of patients achieving LDL-C < 100 mg/dL; the changes of HDL-C, TG, non-HDL-C, Apo A1 and Apo B, fasting plasma glucose, fasting insulin level, insulin resistance by the HOMA-IR, HbA1c, free fatty acid, and ADMA [Time Frame: Baseline to 4 weeks and 12 weeks] [Designated as safety issue: No]

Estimated Enrollment: 200
Study Start Date: July 2011
Estimated Study Completion Date: July 2012

Estimated Primary Completion Date: March 2012 (Final data collection date for primary outcome measure)

Arms	Assigned Interventions
**Pitavastatin**: Experimental<br>Intervention: Drug: **Pitavastatin**	Drug: **Pitavastatin**<br>2 mg QD<br>Other Name: **Livalo**
Atorvastatin: Active Comparator<br>Intervention: Drug: Atorvastatin	Drug: Atorvastatin<br>10 mg QD<br>Other Name: Lipitor

Eligibility

Ages Eligible for Study: 20 Years to 75 Years
Genders Eligible for Study: Both
Accepts Healthy Volunteers: No

Criteria

Inclusion Criteria:

- Patient aged ≥ 20 years old and < 75 years old.
- Patient who was eligible and able to participate in the study and accepts to enter the study by signing written informed consent.
- Patient with fasting LDL-C > 100 mg/dL. The concentration of LDL-C is obtained from laboratory examination.
- Patient with at least one of the following description (NCEP ATP III guideline).
- Female patient with child-bearing potential must take reliable contraception method(s) during the participation of the study.

Exclusion Criteria:

- Patient who has participated in other investigational studies within 3 months.
- Patient took medication and natural health foods known to alter blood lipid profiles within 4 weeks.
- Patient is taking any medication or food that is prohibited by the study.
- Patient taking Amiodarone will be excluded from this study (due to long half life of this medication).
- Patient is diagnosed with type 1 DM or has been using insulin/insulin analog medication.
- Patient with a history of multiple drug allergies or with a known allergy to HMG-CoA reductase inhibitors.
- Patient with TG > 400 mg/dL.
- Excessive obesity defined as BMI above 35 kg/m2.
- Cerebral vascular disease (including cerebrovascular hemorrhage or ischemia, transient ischemic attack) diagnosed within 3 months.
- Myocardial infarction, heart failure (NYHA class III or IV), gross cardiac enlargement (cardiothoracic ratio > 0.5), significant heart block or cardiac arrhythmias within 3 months; history of uncontrolled complex ventricular arrhythmias, uncontrolled atrial fibrillation/flutter or uncontrolled supraventricular tachycardia, pacemaker or implantable cardiac device were not eligible for this study.
- Patient with advanced renal disorder (Serum creatinine levels ≥ 2 mg/dL and BUN ≥ 25 mg/dL).
- Patient with advanced hepatic disorder (AST or ALT level ≥ 100 IU/L)
- Patient with CK level > 5 × ULRR at any time point between Visit 1 and Visit 2.
- Patient with poorly controlled diabetes mellitus (HbA1c > 9.0%) or patient with severe hypertension (> 180 mmHg for systolic or > 120 mmHg in diastolic blood pressure).
- Patient with hypothyroidism, hereditary muscular disorders, family history of the above or history of drug-induced myopathy.
- Patient has significant alcohol consumption (> 65 mL pure alcohol) within 48 hours before Visit 2.
- Any major surgery within 3 months prior to Visit 2.
- Female patient who is lactating, being pregnant or plans to become pregnant.
- Patient with conditions judged by the investigator as unsuitable for the study.

Contacts and Locations

Please refer to this study by its ClinicalTrials.gov identifier: NCT01386853

Contacts

Contact: Jasmine Chao +886-27423012 jasmine@tanabe.com.tw

Locations

Taiwan

Changhua Christian Hospital
Chang-hua, **Taiwan**

National Cheng Kung University Hospital
Tainan, **Taiwan**

Chang Gung Memorial Hospital-LinKou
Taipei, **Taiwan**

Tri-Service General Hospital
Taipei, **Taiwan**

National Taiwan University Hospital
Taipei, **Taiwan**

Taipei Veterans General Hospital
Taipei, **Taiwan**

Sponsors and Collaborators

Tai Tien Pharmaceuticals Co., Ltd.

Investigators

Principal Investigator: Jaw-Wen Chen, M.D. Ph.D. Taipei Veterans General Hospital, Taipei, Taiwan

More Information

No publications provided

Responsible Party: Manager, Development Department
ClinicalTrials.gov Identifier: NCT01386853 History of Changes
Other Study ID Numbers: TATPITA20101005
Study First Received: June 29, 2011
Last Updated: June 30, 2011
Health Authority: Taiwan : Food and Drug Administration

Keywords provided by Tai Tien Pharmaceuticals Co., Ltd.:
Hyperlipidemia
Pitavastatin
Atorvastatin

Additional relevant MeSH terms:
Pitavastatin
Hyperlipidemias
Dyslipidemias
Lipid Metabolism Disorders
Metabolic Diseases
Atorvastatin
Hydroxymethylglutaryl-CoA Reductase Inhibitors
Anticholesteremic Agents
Hypolipidemic Agents
Antimetabolites
Molecular Mechanisms of Pharmacological Action
Pharmacologic Actions
Enzyme Inhibitors
Lipid Regulating Agents
Therapeutic Uses

ClinicalTrials.gov processed this record on August 23, 2011

Contact Help Desk
Lister Hill National Center for Biomedical Communications, U.S. National Library of Medicine,
U.S. National Institutes of Health, U.S. Department of Health & Human Services,
USA.gov, Copyright, Privacy, Accessibility, Freedom of Information Act
